# PolyGR-containing aggregates link with pathology and clinical features of Alzheimer’s disease

**DOI:** 10.1007/s00401-025-02954-8

**Published:** 2025-11-08

**Authors:** Huong T. Phuong, Rodrigo F. Tomas, Cemal Akmese, Ana Mijares, Isabella M. Gerstin, Shu Guo, Logan R. Bell, Ross Ellwood, Svitlana Yegorova, Stefani K. Ng, Grace Massey, Jennifer Phillips, Alexandra Melloni, Olga Pletnikova, XiangYang Lou, H. Brent Clark, Juan C. Troncoso, Bradley T. Hyman, Stefan Prokop, Laura P. W. Ranum, Lien Nguyen

**Affiliations:** 1 Center for NeuroGenetics, College of Medicine, University of Florida, Gainesville, USA; 2 Department of Molecular Genetics & Microbiology, College of Medicine, University of Florida, Gainesville, USA; 3 Department of Pathology, Immunology and Laboratory Medicine, College of Medicine, University of Florida, Gainesville, USA; 4 Center for Translation Research in Neurodegenerative Disease, College of Medicine, University of Florida, Gainesville, USA; 5 MassGeneral Institute for Neurodegenerative Disease, Massachusetts General Hospital, Boston, USA; 6 Department of Pathology, The Johns Hopkins University School of Medicine, Baltimore, USA; 7 Department of Biostatistics, University of Florida, Gainesville, USA; 8 Department of Laboratory Medicine and Pathology, University of Minnesota, Minneapolis, USA; 9 McKnight Brain Institute, University of Florida, Gainesville, USA; 10 Department of Neurology, College of Medicine, University of Florida, Gainesville, USA; 11 Norman Fixel Institute for Neurological Disease, University of Florida, Gainesville, USA; 12 Genetics Institute, University of Florida, Gainesville, USA; 13 Present Address: Department of Pathology and Anatomical Sciences, Jacobs School of Medicine and Biomedical Sciences, University at Buffalo the State University of New York, Buffalo, USA

**Keywords:** Polymeric glycine–arginine, Protein aggregates, Aβ plaques, pTau, AD non-genetic factors, Repeat expansion variants

## Abstract

Alzheimer’s disease is the most common form of dementia; however, its molecular mechanisms are not fully understood. We recently identified polymeric glycine–arginine-containing (polyGR+) aggregates as a novel type of proteinopathy in AD autopsy brains. Here, we performed a comprehensive analysis to study if polyGR+ aggregates are associated with AD neuropathological changes (ADNC) and clinical features of AD cases. We show polyGR+ aggregates are detected in ~ 60% of AD postmortem brains from three AD cohorts but not age-similar controls or disease controls with primary age-related tauopathy (PART). A subtype of polyGR+ aggregates with a clustered-punctate morphology that is positive for the markers of dystrophic neurites is associated with earlier onset and shortened survival in AD cases. Increased levels of Aβ plaques and phosphorylated tau (pTau) tangles are detected in the hippocampus of AD autopsy brains with high levels of polyGR+ aggregates compared to AD autopsy brains with minimal polyGR+ staining. In addition to ADNC, a subset of polyGR+ aggregates coexists with limbic-predominant age-related TDP-43 encephalopathy neuropathological changes (LATE-NC) or Lewy body pathology (LBP). Hippocampal polyGR+ aggregate levels are ~ 3.8- and ~ 3.71-fold higher in late-onset AD cases who experienced stroke or high blood pressure, respectively. In SH-SY5Y cells, hydrogen peroxide treatment which mimics oxidative stress leads to increased levels of polyGR+ proteins produced by the *CASP8* GGGAGA repeat expansion, which was recently shown to associate with increased AD risk. In addition, we show the accumulation of pTau induced by *CASP8* polyGR+ protein aggregates is elevated upon hydrogen peroxide treatment. In summary, our results demonstrate polyGR+ aggregates are a frequent and understudied type of proteinopathy in AD autopsy brains and that polyGR proteinopathy is associated with ADNC.

**Supplementary Information:**

The online version contains supplementary material available at 10.1007/s00401-025-02954-8.

## Introduction

Alzheimer’s disease (AD), the most common form of dementia, accounts for ~ 60–80% of total dementia cases [6, 24]. There were ~ 6.9 million AD cases reported in the US in 2024 and this number is predicted to double by 2060 [1]. AD is characterized by progressive memory loss and AD neuropathological changes (ADNC) including the accumulation of extracellular beta-amyloid (Aβ) plaques and intracellular neurofibrillary tangles (NFTs) composed of hyperphosphorylated tau proteins (pTau) in patient autopsy brains [60, 96]. Additional neuropathological signatures of AD brains include neuronal and synaptic loss, microgliosis, astrogliosis, cerebral amyloid angiopathy, white matter rarefaction, granulovacuolar degeneration, α-synuclein (α-syn) proteinopathy, and limbic-predominant age-related TDP-43 encephalopathy (LATE) characterized by phosphorylated TDP-43 inclusions [26, 28, 32, 47, 61, 62, 71, 91]. The clinical diagnosis of AD is based on a combination of memory and cognitive assessments and evidence of Aβ and pTau deposition through examination of biomarkers in bio-fluids (e.g., blood and cerebrospinal fluid) or imaging techniques [24].

Duplicated amyloid beta precursor protein (*APP*) and rare mutations in *APP*, presenilin 1 (*PSEN1*) and/or presenilin 2 (*PSEN2*) explain ~ 10% of early-onset AD [13]. These genetic abnormalities were shown to increase Aβ production and the Aβ_42_/Aβ_40_ ratio [53, 94, 115]. In contrast, our understanding of the genetic and molecular causes of Aβ and pTau accumulation in sporadic late-onset AD cases (LOAD), which accounts for ~ 95% of the total AD cases, remains limited [3, 8, 38, 98, 114]. Familial studies, genome-wide association and epidemiological studies on LOAD cases have identified more than 80 risk genes or loci (e.g., *APOE4*, *TREM2*, and *ABCA7*) and non-genetic risk factors (e.g., brain injury, high blood pressure, high cholesterol, depression or anxiety, heart disease, diabetes, and cancer) [18, 23, 72, 80, 97, 104]. A subset of these factors is thought to alter *APP* processing, which can lead to the accumulation of Aβ species**.** Multiple studies have shown that the accumulation of Aβ can alter protein kinases involved in tau phosphorylation, resulting in changes in pTau levels and pTau accumulation [8, 29, 82, 114]. The build-up of pTau tangles could lead to a positive feedback loop that worsens Aβ pathology. Consistent with this hypothesis, depletion of tau was shown to protect against amyloid toxicity [8, 53, 84]. Interestingly, accumulation of pTau is also found in other diseases including frontotemporal dementia (FTD), Parkinson’s disease (PD), Huntington’s disease (HD), and various types of spinocerebellar ataxia [15, 27, 30, 76, 83, 113, 116]. Taken together, these data demonstrate a complex interplay of the two classic neuropathological hallmarks of AD and suggest other factors may contribute to the formation of these neuropathologies and the pathogenesis of the disease.

Our previous study of a cohort of 80 AD and 20 control autopsy brains showed that polymeric glycine–arginine-containing (polyGR+) protein aggregates in the hippocampus were detected in 45/80 AD autopsy brains, and polyGR+ aggregate levels were associated with increased pTau levels [68]. In 2011, repeat expansion mutations were shown to express repeat-associated non-AUG (RAN) proteins in all reading frames without the need for an AUG or AUG-like codon [118]. Later polyGR RAN proteins were shown to be expressed from the *C9orf72* G4C2•G2C4 repeat expansion mutation and accumulate as aggregates in the brain and spinal cord of amyotrophic lateral sclerosis and frontotemporal dementia (C9-ALS/FTD) patients [5, 65, 120]. Among the six different C9-RAN dipeptide proteins produced by the *C9orf72* G4C2•G2C4 repeat expansion mutation, polyGR is one of the most toxic protein species and has been shown to cause cell death and disease-relevant phenotypes in model systems [19, 21, 50, 56, 64, 117].

In AD autopsy brains, Nguyen et al., 2025 showed a subset of polyGR+ aggregates is caused by an interrupted GGGAGA repeat expansion within a SINE/VNTR/Alu (SVA) retrotransposable element in intron 8 of the caspase-8 (*CASP8-*GGGAGA^EXP^) [68]*.* The *CASP8-*GGGAGA^EXP^ was shown to produce polyGR+ proteins that accumulate in *CASP8-*GGGAGA^EXP^ (+) AD autopsy brains. A specific interrupted sequence variant of the *CASP8-*GGGAGA^EXP^, which is associated with a ~ 2.2-fold increase in AD risk, produced higher levels of polyGR+ proteins in transfected cells compared with a common *CASP8-*GGGAGA^EXP^ variant [68]. Due to sequence interruptions within the repeat tract, the interrupted *CASP8-*GGGAGA^EXP^ is predicted to produce hybrid GR repeat proteins that contain interspersed stretches of polyGR, polyRE, and polyGE. Interestingly, expression of synthetic *CASP8-*GGGAGA^EXP^ minigenes increased pTau levels in transfected SH-SY5Y cells [68].

Here, we aim to address the following questions: 1) Are polyGR+ aggregates consistently found in a larger cohort of AD autopsy brains? 2) Do polyGR+ aggregate levels associate with other known AD neuropathological changes or other comorbidities in AD cases? 3) What is the pathological contribution of polyGR+ aggregates to AD? To address these questions, we performed immunohistochemistry (IHC) staining on hippocampal sections of autopsy brains from three expanded AD and three expanded control cohorts with a total combined number of 156 AD cases, 26 age-similar controls, and 13 disease controls to study the association of polyGR+ aggregates with Aβ and pTau pathologies as well as the clinical features of AD patients. Cellular experiments were also performed to further study the toxic effects of polyGR+ proteins expressed by the *CASP8-*GGGAGA^EXP^. Our results show that polyGR+ aggregate pathology is strongly associated with known AD neuropathological hallmarks, non-genetic factors of AD, and altered tau phosphorylation.

## Materials and methods

### Patient samples

**Fig. 1:**
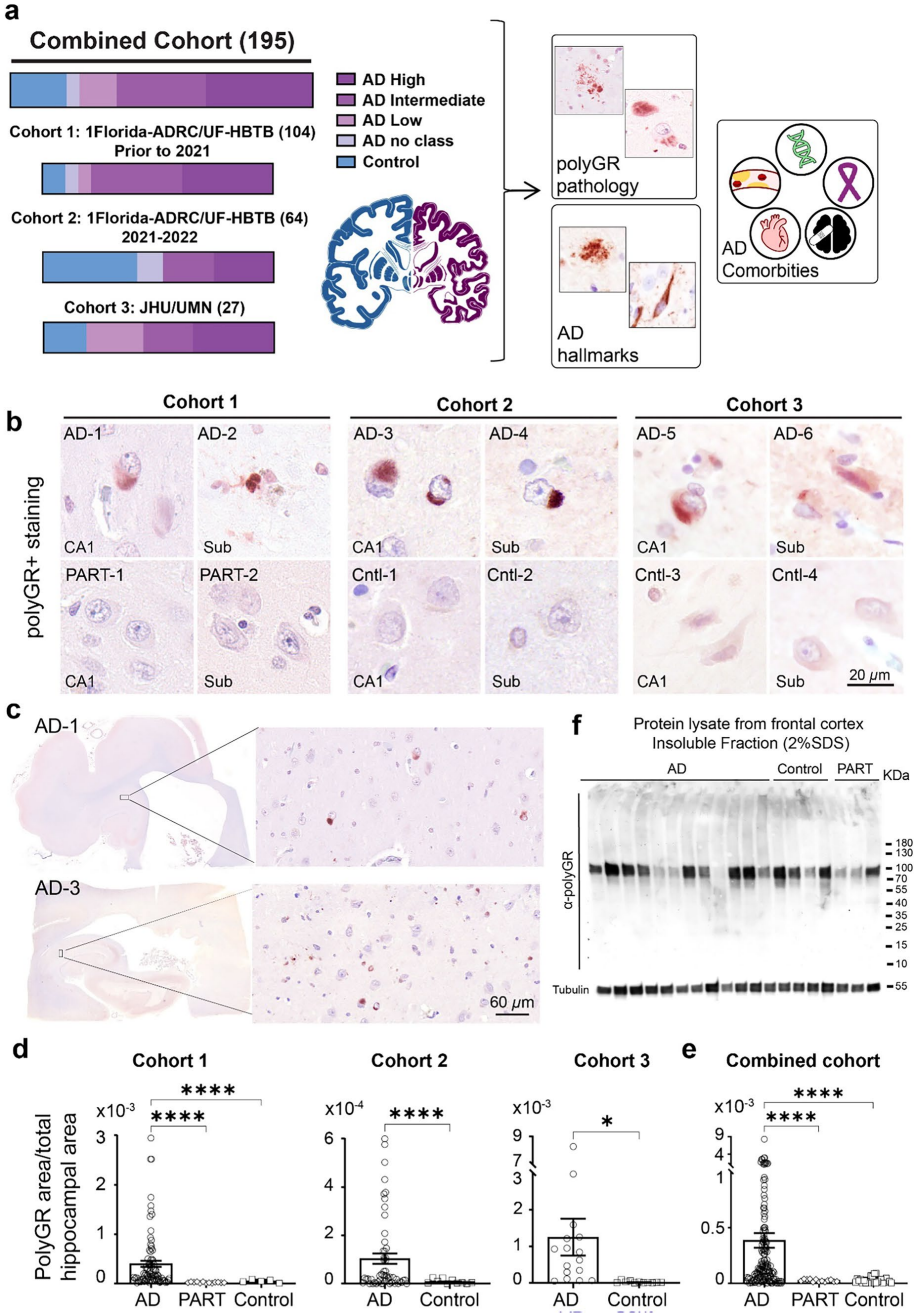
PolyGR+ aggregates are frequently detected in the hippocampus in autopsy brains from multiple cohorts of AD. **a** A schematic diagram showing the three independent cohorts of AD and control cases and experimental workflow. (Left) Cohort 1: 1Florida Alzheimer’s Disease Research Center (ADRC)/ UF Neuromedicine Human brain and tissue bank (UF-HBTB) includes 86 AD, 13 Primary Age-Related Tauopathy (PART) and 5 control cases collected prior to 2021. Cohort 2: 1Florida-ADRC/UF-HBTB includes 54 AD and 10 control cases collected during 2021 to 2022. Cohort 3: -JHU/UMN includes 16 AD and 11 control cases from John Hopkins University (JHU) and the University of Minnesota (UMN). (Right) Accumulation of polyGR+ aggregates and its association with AD neuropathological changes and comorbidities (e.g., cancer, heart disease, brain injuries, high blood pressure, and genetic risk factors) are examined (polyGR antibody: H3148, Aβ antibody: Ab5, pTau antibody: AT8). **b** Example of polyGR+ aggregates staining in hippocampus of AD, PART, and Control autopsy brains from three cohorts (Sub: Subiculum, CA: Cornu Ammonis). **c** Widefield and zoomed-in images showing polyGR+ aggregate staining in the hippocampus of AD autopsy brains. **d**, **e** Quantification of polyGR+ aggregate staining in the hippocampal regions from AD autopsy brains from the three independent cohorts (**d**) and the combined cohort (**e**) compared to control cases. **f** Image of Western blotting using α-polyGR antibody and denatured proteins from insoluble protein fractions extracted from frozen frontal cortex of AD (*n* = 12), Control (*n* = 4) and PART (*n* = 3) cases. Data represent mean ± SEM. Statistical analyses were performed using one-way ANOVA with Brown–Forsythe test (**d**, cohort 1 and **e**) and unpaired two-tailed Welch’s *t* test (**d**, cohort 2 and 3). Sex and gender were also taken into consideration with an ANCOVA analysis. *p < 0.05, ****p < 0.0001

Brain tissue samples were independently collected by the Johns Hopkins brain bank, the University of Minnesota brain bank, the 1Florida Alzheimer’s Disease Research Center (ADRC) and the UF Neuromedicine Human Brain and Tissue Bank (UF-HBTB) (Fig. 1a and Table 1). Postmortem brains were immersion fixed in formalin and individual brain regions were subsequently embedded in paraffin wax. The tissue samples were sectioned using a Leica RM2235 microtome with a thickness of ~ 7 µm and put on microscope glass slide. The samples in this study were collected in accordance with the Declaration of Helsinki. Written, informed consent was obtained from participants or relevant parties at the time of enrollment. The information of AD and Control cohorts including age of onset, age at death, disease duration, postmortem interval (PMI), gender, and medical historical records (e.g., TBI, stroke, high blood pressure, high cholesterol, anxiety, and some other diseases) was provided. The inclusion and exclusion criteria for AD cases, controls and PART cases were based on the clinical information on cognitive performance and the pathological assessment of autopsy brains. AD cases were defined as those with memory loss and confirmed ADNC at autopsy. Control individuals were defined as those without memory loss and up to low ADNC at autopsy (Braak stage ≤ 2, Thal phase ≤ 2). PART cases were defined as low to intermediate NFT pathology (Braak stage from I-IV) with no or low Aβ pathology (Thal phase from 0 to 2). The inclusion and exclusion criteria for the comorbidities including TBI, stroke, high blood pressure, high cholesterol, anxiety, and other medical conditions were based on diagnosis information in the medical historical reports. Frozen tissue samples from AD and control cases were collected at the Massachusetts Alzheimer’s Disease Research Center, and genomic DNA samples were extracted from these frozen tissue samples to clone *CASP8*-GGGAGA^EXP^ minigenes (p-AD-R1 and p-C-Var plasmids) as described in Nguyen et al., *PNAS* paper [68]. The study was approved by institutional review boards of the University of Florida, the Johns Hopkins University, the University of Minnesota, and the Massachusetts General Hospital.

**Table 1:** Demographic information and pathological assessment of AD and control cases in our study

	Combined 195	Control/PART 39	AD Low 21	AD Intermediate 48	AD High 80	AD 7
Disease classification						
Age of onset (S.D)	69.2 ± 10.9	**–**	69.9 ± 12.3	68.4 ± 9.3	69.7 ± 11.5	69.7 ± 18.0
Early-onset of AD (EOAD)	46	**–**	6	15	22	3
Late-onset of AD (LOAD)	97	**–**	14	29	51	3
Age of death (S.D)	77.3 ± 10.7	73.0 ± 12.8	75.8 ± 8.2	77.6 ± 10.6	79.7 ± 10.1	73.1 ± 12.2
Median postmortem Interval (h)	14.6	12	48	12	15	8
Duration						
< 5 years	20	**–**	8	6	5	1
> 5 years	77	**–**	8	27	40	2
Family history						
Yes	66	4	8	19	34	1
No	73	7	12	18	34	2
Sex						
Female	95	16	12	20	44	3
Male	100	23	9	28	36	4
Pathological assessment						
Braak stage (*n* = 165)						
0	3	3	**–**	**–**	**–**	**–**
1	9	6	3	**–**	**–**	**–**
2	14	8	2	2	2	**–**
3	11	1	2	6	**–**	2
4	45	**–**	10	16	19	**–**
5	37	**–**	3	7	26	1
6	46	**–**	1	13	29	3
Thal phase (*n* = 157)						
0	10	10	**–**	**–**	**–**	**–**
1	14	6	7	**–**	**–**	1
2	7	1	4	**–**	1	1
3	19	**–**	5	10	4	**–**
4	26	**–**	3	10	13	**–**
5	81	**–**	2	22	55	2
CERAD score (*n* = 166)						
None	33	18	13	**–**	**–**	2
Sparse	10	**–**	2	6	2	**–**
Moderate	18	**–**	1	13	4	**–**
Frequent	105	**–**	5	26	70	4
LATE (TDP**-**43) (*n* = 131)						
0	98	**–**	17	30	48	3
1	8	**–**	**–**	1	7	**–**
2	24	**–**	1	9	13	1
3	1	**–**	**–**	**–**	1	**–**

*PART* primary age-related tauopathy, *EOAD* early-onset AD, *LOAD* late-onset AD, *Braak stage* quantifying neurofibrillary tangle pathology in the brain; *Thal phase* quantifying amyloid plaques in the brain, *CERAD score* (Consortium to Establish a Registry for Alzheimer’s disease) assessing neuritic plaques in the brain, *LATE (TDP-43)* (limbic-predominate age-related TDP-43 encephalopathy)

### Immunohistochemical (IHC) staining

Seven micrometer tissue slides were deparaffinized at a 60 °C incubator in ~ 30 min and then cooled down to room temperature (RT). The slides were deparaffinized by xylene twice for 10 min, followed by rehydration through a decreasing graded ethanol series  (100% → 100% → 95% → 80%, 5 min each), and then stopped by rinsing with tap water. Antigen retrieval was carried out using a steamer (~ 100 °C) for 30 min with incubated slides in 10 mM citrate buffer (pH 6.0). After antigen retrieval, the slides were cooled to RT over 1–2 h before being washed in running tap water for 10 min. The slides were then incubated in ≥ 95% formic acid for 5 min followed by washing in running tap water for 10 min. To block endogenous peroxidase activity, slides were incubated in 3% H_2_O_2_ (freshly prepared in 1xPBS) for 15 min, then washed in running tap water for 15 min. To block nonspecific binding and excessive background, slides were incubated for 30 min with Background Sniper (BS), a serum-free block solution (Biocare Medical. SKU# BS966MM). The primary antibody was prepared in 10% BS (in water) and applied on the top of the slides and incubated overnight at 4 °C. During the second day, the slides were incubated at RT for an hour. The primary antibody was washed out of the samples with 1xPBS three times. The samples were then incubated with the secondary antibody, HRP-conjugated anti-rabbit or anti-mouse IgG antibody (Vector Labs, Ref# PI-1000-1 or PI-2000-1, 1:1000), for 1 hr at RT. After four washes with 1XPBS, the slides were developed with NovaRed HRP (Vector laboratories, SK-4800). Development time is optimized for specific tissue regions and antibodies. The slides were then washed in running tap water for 10 min, counterstained with hematoxylin for 1–2 min (modified Harris, Sigma-Aldrich) and washed again with running tap water for 10 min. Afterward, the slides were dehydrated in an increasing graded ethanol series (80% → 95% → 100% → 100%) and xylene. Finally, the slides were mounted with Cytoseal 60 (LOT 18006) and covered with glass slips for visualization.

#### Antibodies

The primary antibodies used in the IHC staining include rabbit polyclonal antibody α-polyGR (H3148, 1:5000 with IHC single staining, 1:1000 with double staining), mouse monoclonal antibody α-Aβ (Ab5, 1:5000, Thermo Fisher Scientific, Cat# MA5-48043), mouse monoclonal antibody α-p-Tau (AT8, 1:2000, Thermo Fisher Scientific, Cat# MN1020), and mouse polyclonal antibody pTDP-43 ([Ser409/410], 1:1000, Cosmo Bio, Cat# CAC-TIP-PTD-M01). The secondary antibodies consisted of HRP-conjugated anti-mouse IgG (Vector Labs, Ref# PI-2000-1, 1:1000) or anti-rabbit IgG (Vector Labs, Ref# PI-1000-1) according to the host–primary antibody.

### Double IHC staining of polyGR with cell type markers (NeuN, GFAP, and Iba1), Aβ plaque, pTau, pTDP-43, or α-syn

The IHC protocol was similar to above unless stated otherwise. During the first day, BLOAXALL Blocking Solution (Vector Laboratories, SK-4805) was utilized to inactivate endogenous peroxidase, pseudoperoxidase, and alkaline phosphatase. PolyGR (H3148, 1:5000) was co-stained with either NeuN mouse monoclonal antibody (1:2000, Sigma-Aldrich, Cat# MAB377, clone A60), GFAP mouse monoclonal antibody (1:2000, Thermo Fisher Scientific, Cat# 14-9892-82, GA5), Iba1 mouse monoclonal antibody (1:2000, Thermo Fisher Scientific, Cat# MA5-27726, GT10312), α-Aβ (1:5000, Thermo Fisher Scientific, Cat# MA5-48043, Ab5), pTau-1:5000 (AT8), pTDP-43 (1:20,000, Cosmo Bio USA, Cat# CAC-TIP-PTD-M01, Clone 11-9), or α-syn (1:20,000, 94-3A10)*.* The second day of staining was similar unless stated otherwise. Following the first three rounds of 1xPBS washes, the VectorLabs ImmPRESS duet Duet Double Staining Polymer (HRP—Anti-Rabbit IgG-brown, AP—Anti-Mouse IgG-magenta) (MP-7714-15) kit was used to develop the co-stains following the manual. Briefly, the slides were incubated with ImmPRESS Duet Reagent for 20 min and then washed three times with 1XPBS. ImmPACT DAB EqV Substrate was added to the slides to develop HRP-conjugated secondary antibodies. Slides were washed in running water for 5 min. ImmPACT Vector Red Substrate was added to the slides to develop AP-conjugated antibodies. Slides were washed in running water for 5 min and counterstained with hematoxylin for 1–2 min (modified Harris, Sigma-Aldrich) and then washed again with running tap water for 10 min. The slides were dehydrated in an increasing graded ethanol series (80% → 95% → 100% → 100%) and xylene. Finally, the slides were mounted by Cytoseal 60 (LOT 18006) and covered with glass slips for visualization.

### Double immunofluorescence (IF) staining of polyGR with pTDP-43

The experiment was carried out similarly to the above IHC protocol with some modifications following: on the first day of IHC staining, the slides were incubated with a mixture of primary antibodies (rabbit α-polyGR, H3148, 1:1000, and mouse α-pTDP-43, Ser409/410, 1:1000). On the second day, the slides were incubated with a mixture of secondary antibodies Alexa Fluor 555-conjugated goat anti-rabbit IgG (1:1000, ThermoFisher Scientific, Cat# A11008) and Cy5-conjugated AffiniPure goat anti-mouse IgG (Jackson ImmunoResearch, Cat# 115-175-146). After washing out the secondary antibodies, the slides were soaked with 0.1% Sudan Black (prepared in 70% ethanol). The slides were then washed once with PBS-Tween20 (0.5% in 1xPBS) and three times with PBS before being mounted with DAPI counterstain (Vector Labs, Cat# H-1800) for visualization.

### Cell culture

SH-SY5Y cells were cultured in F12 DMEM supplemented with 10% FBS and incubated at 37 °C with 5% CO_2_. Following the manufacturer’s protocol, plasmid transfections were performed using lipofectamine 2000 (Invitrogen). Briefly, cells were transfected with either 2 μg of *CASP8*-GGGAGA^EXP^ expressing plasmids (p-AD-R1 or p-C-Var) or empty vector control plasmids (pcDNA3.1-3xstop) using Lipofectamine^TM^ 2000 Transfection Reagent (Thermo Fisher Scientific, Cat# 11668019). Cells were fixed 24-h post-transfection for subsequent staining.

### Fluorescence in situ hybridization to detect *CASP8*-GGGAGA^EXP^ RNA [r(GGGAGA^EXP^)]

Approximately, 175,000 SH-SY5Y cells were seeded on glass coverslips in 12-well plates 24 h prior to transfection. SH-SY5Y cells were fixed 24 h later in 4% paraformaldehyde (PFA) (Electron Microscopy Sciences, Cat# 15710) in 1xPBS for 20 min and permeabilized with 0.5% Triton X-100 (Fisher, Cat# 9002-93-1) for 20 min. After washing with 1xPBS, the cells were blocked with hybridization buffer (50% formamide [Sigma-Aldrich, Cat# 47671], 2xSSC [Invitrogen, Cat# 15557-044], 0.1% BSA [Fisher, Cat# BP9703100], 10% Dextran-Sulfate [Sigma-Aldrich, Cat# D6001]) for 1 h at 42 °C. The (TCTCCC)_4_-Cy5 FISH probe was refolded at 95 °C for 5 min and cooled to RT over 30 min. The refolded FISH probe was then added to the hybridization buffer to achieve a final probe concentration of 0.05 µM. This complete hybridization buffer was applied to the cells and incubated overnight at 42 °C. Subsequently, the cells were sequentially washed at 44 °C with 50% formamide/2x SSC and 2xSSC, followed by a 1xPBS wash at room temperature. Finally, the cells were mounted with DAPI counterstain (Vector Labs, Cat# H-1800) and stored at 4 °C until imaging under the LSM 880 confocal microscope (Zeiss).

### Immunofluorescence (IF)

For IF experiments, after staining the coverslips were mounted with medium containing DAPI (ThermoFisher Scientific, P36935). The IF images were obtained using Confocal Microscopy LMS 880 (Zeiss).

*Effects of CASP8*-*GGGAGA*^*EXP*^ *polyGR on phosphorylation of tau (p-tau) protein in SH-SY5Y.* SH-SY5Y cells were seeded and transfected as described above. Following 24 h after transfection, cells were fixed with 4% PFA in PBS for 20 min at room temperature (RT) and permeabilized in 0.5% triton X-100 in 1xPBS for 20 min at RT. Three 5-min washes with 1xPBS were done after fixation and permeabilization. The cells were blocked in 1% normal goat serum (NGS) in PBS for 1 h at RT. After blocking, the cells were incubated with the primary antibody rabbit α-polyGR (H3148, 1:1000) and mouse α-pTau (AT8, 1:1000, ThermoFisher Scientific, Cat# MN1020) in blocking buffer at 4 °C overnight. The next day after three 1xPBS washes (5 min each), cells were incubated with secondary antibodies Alexa Fluor 555-conjugated goat anti-rabbit IgG (1:1000, ThermoFisher Scientific, Cat# A11008) and Cy5-conjugated AffiniPure goat anti-mouse IgG (Jackson ImmunoResearch, Cat# 115-175-146) to detect polyGR and p-tau respectively.

*Effects of hydrogen peroxide (*H_2_O_2_*) on levels of CASP8 RAN proteins and p-tau.* SH-SY5Y cells were seeded as described above. Cells were transfected for 12 h prior to treatment with 50 μM of hydrogen peroxide (H_2_O_2_). After an additional 12 h of incubation, cells were fixed and permeabilized as described above. Cells were incubated with the rat monoclonal α-polyGR (1:1000, ThermoFisher Scientific, Cat# A-21094) and mouse α-pTau (AT8, 1:1000, ThermoFisher Scientific, Cat# MN1020). The secondary antibodies were Alexa Fluor 488-conjugated goat anti-rat IgG (1:1000, ThermoFisher Scientific, Cat# A11006-) and Cy5-conjugated AffiniPure goat anti-mouse IgG (1:1,000, Jackson ImmunoResearch Cat# 115-175-146).

*CASP8*^*EXP*^* RNA transcript levels in hydrogen peroxide-treated SH-SY5Y.* Approximately 225,000 SH-SY5Y cells per well were seeded in 6-well plates 24 h prior to transfection. Cells were transfected with either 5 μg of *CASP8*-GGGAGA^EXP^ plasmids (AD-R1 or C-Var) or pcDNA3.1-3xstop plasmids using Lipofectamine^TM^ 2000 Transfection Reagent. 12 h following transfection, SH-SY5Y cells received either media or 50 μM of hydrogen peroxide. RNA extraction was performed using TRIzol (Invitrogen) following the manufacturer’s protocol. DNA contamination was eliminated by treating the RNA samples with TURBO^TM^ DNase (Invitrogen, cat# AM1907) followed by inactivation with an additional TRIzol RNA extraction. Total RNA was reverse-transcribed using SuperScript III RT kit (Invitrogen) and random-hexamer primers (Applied Biosystems). Quantitative RT-PCR was performed using the PowerSYBR system (Applied Biosystems) using specific primers (Table S3) and the AB Step One Plus Real-Time PCR system following the manufacturer’s protocol and the PCR program described above. For 3xFlag-CASP8-RE-3T transcript levels: F-3’UTR-pcDNA3.1 and R-3’UTR-pcDNA3.1, for GAPDH: GAPDH-F2 and GAPDH-R2.

### Hybridization chain reaction fluorescence in situ hybridization (HCR-FISH) for the *CASP8*-GGGAGA^EXP^

The experiment was carried out similarly to the above IHC protocol with some modifications. Following antigen retrieval, slides were washed with 2xSSC [ThermoFisher, Cat# 15557044], and blocked with 30% probe hybridization buffer (30% formamide [Sigma-Aldrich, Cat# 47671], 5xSSC [ThermoFisher, Cat# 15557044], 10% Dextran-Sulfate [Sigma-Aldrich, Cat# D6001], 9 mM Citrate Buffer (pH6), 50 mg/mL Heparin (Sigma Cat. # H3393), 1xDenhardt’s solution (Life Technologies Cat. # 750018), 0.1% Tween20 (BioRad Cat. # 161-0781) for 30 min at 37 °C. The *CASP8*-GGGAGA probe 1 and 2 were refolded at 95 °C for 90 s and added to the 30% probe hybridization buffer at a final concentration of 0.004 µM. This complete hybridization buffer was applied to the tissue and incubated overnight at 37 °C. Subsequently, the tissues were sequentially washed at 37 °C with 30% probe wash buffer (30% formamide, 5xSSC, 9 mM Citrate Buffer, 0.1% Tween20, 50 mg/mL Heparin) followed by 2 washes of 5xSSCT (5xSSCT and 0.1% Tween20) at room temperature. The tissue was incubated with amplification buffer (5xSSCT, 0.1% Tween20, 10% Dextran-Sulfate) at RT for 30 min. The initiator complex I1 and I2 were refolded at 95 °C for 90 s and cooled at room temperature for 30 min in the dark. The initiator complex was added to the amplification buffer at a final concentration of 18 pmol. The slides were left in the dark overnight at RT. Finally, the cells were washed with 5xSSCT 4 times. 0.1% Sudan black was used to reduce background staining in the tissue for 2 min and mounted with DAPI counterstain (Vector Labs, Cat# H-1800) and stored at 4 °C until imaging under the LSM 880 confocal microscope (Zeiss).

### Genotyping AD-risk SNPs

Information of nine AD-associated single nucleotide polymorphisms (SNPs) used in the study is summarized in Table 2. Genotyping was assessed on 126 AD samples by TaqMan genotyping SNP assay (Thermo Fisher, cat# 4331349). The PCR reaction included SNP probes, TaqMan genotyping master mix (Thermo Fisher, cat# 4371355) and genomic DNA. The SNP PCRs were performed and monitored by a real-time PCR instrument (BioRad, CFX opus 384). The major and minor alleles of each SNP were detected based on qualification of VIC and FAM fluorophore signals. For *APOE* genotyping, we used two SNPs, the rs429358 that links with *APOE4* and the rs7412 that links with *APOE2*. The *APOE* genotypes are detected based on quantification of fluorophore signal of either rs429358 or rs7412 or both (Table S1).

**Table 2:** List of AD-associated single nucleotide polymorphisms (SNPs) included in our study and TaqMan SNP genotyping assays

Database SNPs	Major → minor allele	Chromosome	Location (GRCh38)	Gene	Assay ID
rs429358	T→C	chr19	19:44908684	*APOE*	C_3084793_20
rs7412	C→T	chr19	19:44908822	*APOE*	C_904973_10
rs75932628	C→T	chr6	6:41161514	*TREM2*	C_100657057_10
rs11218343	T→C	chr11	11:121564878	*SORL1*	C_31696474_10
rs5848	C→T	chr17	17:44352876	*GRN*	C_7452046_20
rs744373	A→G	chr2	2:127137039	*BIN1*	C_1042213_10
rs3173615	C→G	chr7	7:12229791	*TMEM106B*	C_27465458_10
rs3851179	T→C	chr11	11:86157598	*PICALM*	C_8748810_10
rs115550680	A→G	Chr19	19: 1050421	*ABCA7*	C_153371549_10

### Western blot

Postmortem brain lysate from AD, control and PART samples was collected in a 1.5 mL Eppendorf tube (~ 40 mg) and homogenized in 400 µl of RIPA buffer (G Biosciences, Cat# 786-489) supplemented with proteinase inhibitor cocktail (TargetMol, Cat# C0001) and phosphatase inhibitor cocktails I (TargetMol, Cat# C0002) and II (TargetMol, Cat# C0003). Approximately 20 μg of total protein was loaded per well on a precast gel (4%–15% Tris–Glycine extended, Criterion, Cat# 5671085) and transferred to a nitrocellulose membrane (Amersham). After overnight blocking at 4 °C with 5% skim milk in PBS buffer containing 0.05% Tween20 (PBS-T), the membrane was washed with PBS-T, three times, 5 min each, and then probed with primary antibody rabbit α-polyGR (H3148, 1:5000) overnight at 4 °C. The following day, the membrane was incubated at RT for 1 h. After four washes with PBS-T (5 min/each wash), the membrane was incubated with secondary ECL anti-rabbit IgG (Amersham, Cat# NA934V) for 1 h at RT. PolyGR and secondary antibody complex were detected using western lightning enhanced chemiluminescence reagent plus-ECL (Cat# 101677-048) on ProSignal ECL blotting film (Cat# 30-507L).

### Statistical analysis

For whole slide IHC (QuPath), including polyGR, beta-amyloid, and pTau (AT8) staining in the CA, Dentate gyrus, and subiculum subfields of the hippocampal section, samples were quantified using whole scanned images and QuPath version 0.2.3 following a protocol modified from Courtney et al. [85]. Images were analyzed by batch-run using GR_staining_measurement.groovy, pTau_quantification.groovy, polyGR_AT8_Staining_transfected_cells.groovy, and r(GGGAGA)_Staining_transfected_cells.groovy (see attached scripts) to quantify polyGR, beta-amyloid, pTau and, r(GGGAGA) staining, respectively. Analysis of individual images was double-checked by two researchers.

Graphs and plots of IHC staining were generated using GraphPad Prism. For the datasets consisting of three or more groups, data analyses were performed using one-way ANOVA with post hoc Sidak multiple comparison tests. For the datasets with two groups, data analysis was performed using a two-tailed unpaired Welch’s t test. Pearson correlation coefficients were calculated to generate the correlation matrix heatmap for polyGR, Aβ, and pTau. Summary data are presented as mean ± standard error of the mean (SEM). Two-tailed *p* ≤ 0.05 is considered statistically significant. Significance in figures is indicated as follows: ns (not significant *p* > 0.05), ∗ (*p* ≤ 0.05); ∗∗ (*p* ≤ 0.01); ∗∗∗ (*p* ≤ 0.001); ∗∗∗∗ (*p* ≤ 0.0001). To account for sex and gender effects, ANCOVA (equivalent to regression analysis here) and partial correlation analyses were also performed, in addition to the analyses mentioned above. Analysis details and sample sizes (n values) are included in the figure legends. Statistical analyses were implemented using GraphPad Prism (v 10.3.1 for Windows; GraphPad Software, Boston, Massachusetts, US) and R software (v.3; R Development Core Team, Vienna, Austria).

For molecular analysis, all quantification and statistical analyses were performed using at least three biological replicates per experiment. The number of biological replicates was included in the figure legends.

*Quantification of pTau levels in SH-SY5Y cells transfected with plasmids expressing the CASP8-GGGAGA*^*EXP*^*.* Cells were imaged with Zeiss LSM800 confocal microscopy with a 0.0169 μm^2^ pixel resolution. Images were imported into QuPath (Version 0.5.1). A rectangular annotation was added to the full image. Cells with polyGR fluorescence exceeding a set threshold were characterized as positive cells using the built-in Positive-cell detection feature (Analyze > Cell detection > Positive Cell detection). The positive-cell detection channel was set to the DAPI channel with a threshold intensity set to 10 with a sigma adjusted to 1.3 μm and 1 μm cell expansion. Cells with pixel values in the polyGR channel exceeding 20.0 were classified as polyGR-positive cells. The average AT8 fluorescence was automatically measured in every cell. The measurements were exported as a.csv file. The exported values were aggregated and normalized to the average AT8 fluorescence in negative cells by replicate. The plots were generated using GraphPad Prism 10.3.1. Statistical analysis was performed using an unpaired two-tailed Welch’s *t* test.

### Quantification of pTau and polyGR in hydrogen peroxide-treated SH-SY5Y cells expressing* CASP8*-GGGAGA^EXP^

Images obtained with the confocal microscopy LSM800 were imported into QuPath Version 0.51. An annotation plane was added to each image. The number of cells per image was obtained with the use of QuPath’s Cell detection function (analyze > cell detection > cell detection). To minimize an overestimation of cells per slide, the following changes were made to the cell detection parameters: the detection channel was set to DAPI, the sigma was set to 2.5 μM, and the intensity threshold was set to 25. To determine the area with α-polyGR staining, the thresholds were applied using QuPath’s pixel classifier function (classify > pixel classifier > create thresholder). Within the thresholder parameters, the resolution was set to Full (0.13 μM/px), the channel was set to the appropriate fluorescence (594), and a Gaussian prefilter was applied. The threshold to detect polyGR fluorescence was established by screening images from SH-SY5Y cells transfected with *CASP8*-GGGAGA^EXP^ or empty control plasmids. Once the most optimal threshold was established, the parameters were run on all the images, and the data were exported as a.csv file. This was repeated for AT8 (pTau) signals. The exported values were aggregated and normalized to the number of cells per image. The plot was generated using GraphPad Prism 10.3.1. Statistical analysis was performed using one-way ANOVA with Tukey’s multiple comparisons.

### Positive-cell detection for r(GGGGAGA)^*EXP*^*expression in SH-SY5Y cells transfected with CASP8-GGGAGA*^*EXP*^*plasmids*

Cells were imaged with Zeiss LSM800 confocal microscopy with a 0.0169 μm^2^ pixel resolution. Images were imported into QuPath (Version 0.5.1). A rectangular annotation was added to the full image. Cells with polyGR fluorescence exceeding a set threshold were characterized as positive cells using the built-in positive-cell detection feature (analyze > cell detection > positive-cell detection). The positive-cell detection channel was set to the DAPI channel with a threshold intensity set to 10 with a sigma adjusted to 1.3 μm and 3 μm cell expansion. Cells with pixel values in the polyGR channel exceeding 8.0 were classified as positive cells.

## Results

### PolyGR+ aggregate staining is frequently found in multiple cohorts of AD brains

Nguyen et al., previously showed hippocampal polyGR+ aggregates accumulate in 45/80 AD autopsy brains but not in 20 age-similar control cases [68]. Here, we examined polyGR+ aggregates in three expanded independent AD autopsy cohorts to better characterize polyGR pathology in AD. The total number of autopsy brains examined (*n* = 195) includes 156 AD cases, 26 controls, and 13 disease controls with primary age-related tauopathy (PART). Control autopsy brains are defined as free of ADNCs (Braak stage ≤ 2, Thal phase ≤ 2). In addition, we included 13 PART disease control cases, which carry low pTau/NFT stage (Braak stage from I–IV) with no or low Aβ accumulation levels and deposition (Thal phase from 0 to 2). Brain samples were collected at multiple centers and at different time periods (Fig. 1a). Cohort 1 was collected at the 1Florida Alzheimer’s Disease Research Center (ADRC) and UF Neuromedicine Human Brain and Tissue Bank (UF-HBTB) (Cohort 1: 1Florida-ADRC/UF-HBTB; *n* = 104) prior to 2021, which includes 86 AD cases, 5 controls, and 13 PART cases. Cohort 2: 1Florida-ADRC/UF-HBTB (*n* = 64), consists of 54 AD and 10 control cases collected at the 1Florida-ADRC and the UF-HBTB during 2021–2022. Cohort 3: JHU/UMN (*n* = 27) was collected at John Hopkins University’s and the University of Minnesota’s Brain Centers and includes 16 AD and 11 control cases.

In line with Nguyen et al., 2025 [68], IHC staining using a previously developed polyGR antibody [120] shows that polyGR+ aggregates are frequently detected in hippocampal sections from AD autopsy brains from all three cohorts (Fig. 1b and c). In contrast, no similar staining was detected in controls (0/26) or PART (0/13) cases. In AD autopsy brains, polyGR+ aggregates are abundantly found in the cornu ammonis (CA), dentate gyrus (DG), and subiculum (Sub) regions of the hippocampus (Fig. 1b, c and Fig. S1). Quantification of polyGR+ aggregate staining on whole hippocampal sections from AD, control, and PART cases using QuPath shows that polyGR+ aggregate levels are increased in AD autopsy brains compared to controls and PART cases in all three cohorts and the combined cohort (Fig. 1d and e). The *p* values for the polyGR+ aggregate levels comparison between AD vs control and PART cases are 6.07 × 10^−8^ for Cohort 1: 1Florida-ADRC/UF-HBTB, 5.72 × 10^−5^ for Cohort 2: 1Florida-ADRC/UF-HBTB, 0.0279 for Cohort 3: JHU/UMN and 2.22 × 10^−7^ for the combined cohort (Fig. 1d and e). While no similar polyGR+ staining was detected in hippocampal sections from controls or PART cases, some background signal in these cases was detected using automated QuPath analyses. The number of AD cases that show higher levels of polyGR+ aggregate staining compared to the highest signal detected in the control and PART group is 63/86 (~ 73%) for Cohort 1, 28/54 (~ 52%) for Cohort 2, 13/16 (~ 81%) for Cohort 3, and 94/156 (~ 60%) for the combined cohort. In addition, we performed α-polyGR IHC staining in medulla oblongata, pons, cerebellum, as well as occipital cortex and frontal cortex sections from a subset of AD, control, and PART brains. In contrast to the frequent and profound polyGR+ aggregate staining in AD hippocampi, small polyGR+ puncta are occasionally found in the pons, cerebellum, occipital and frontal cortex of AD autopsy brains. No comparable staining was detected in those brain regions from control or PART cases (Fig. S2-6). Western blot using the α-polyGR antibody [120] and denatured proteins from soluble and 2% SDS protein fractions extracted from frozen frontal cortex tissue did not detect a clear difference in protein signal between AD, control or PART cases (Fig. 1f and Fig. S7).

In summary, we show that polyGR+ aggregates accumulate in three independent cohorts and are a frequent type of proteinopathy found in the hippocampus of ~ 60% AD autopsy brains. In contrast, polyGR+ aggregates were not found in disease controls with PART, who have neurofibrillary pTau tangles but no or low Aβ plaques found in AD.

### Specific accumulation patterns of polyGR+ aggregates are associated with age of onset and survival of AD patients

**Fig. 2:**
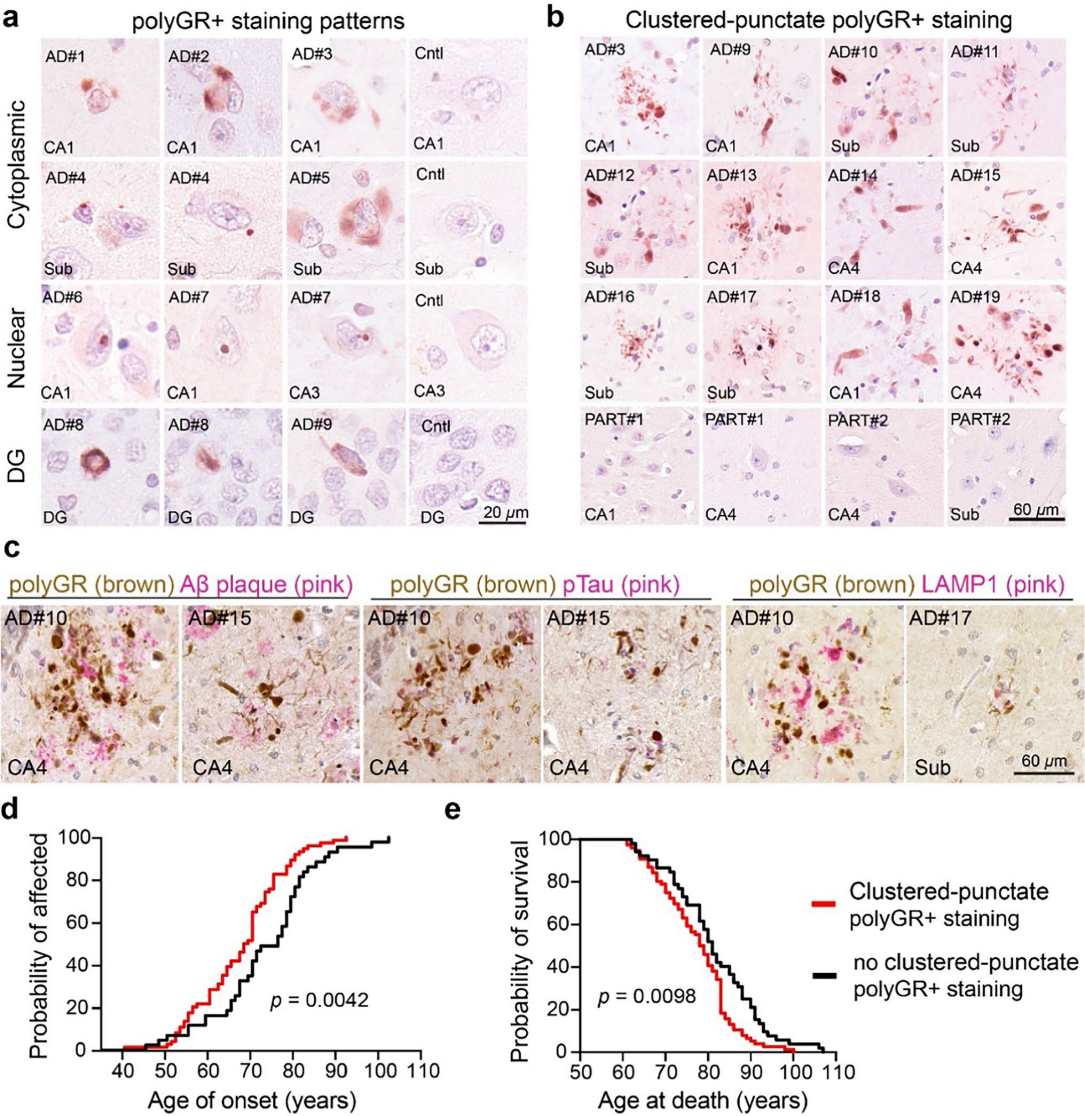
Distinct accumulation pattern of polyGR+ staining is associated with age of onset and age at death of AD cases. **a** Immunohistochemistry (IHC) staining of polyGR+ aggregates (red) in hippocampal sections (Sub: Subiculum, CA: Cornu Ammonis, DG: Dentate Gyrus) from AD and control autopsy brains. Examples of cytoplasmic polyGR aggregates (rows 1 and 2), nuclear polyGR+ staining (row 3), DG polyGR+ staining (row 4). **b** Clustered-punctate polyGR+ aggregates were present in AD autopsy brains and no similar polyGR+ staining was detected in PART cases. **c** IHC co-staining of polyGR aggregates (brown) with either Aβ plaque (pink), pTau(pink), or LAMP1 (pink) in AD hippocampal sections, showing clustered-punctate polyGR+ aggregates are positive for the markers of dystrophic neurites. **d** Comparison of age of onset of AD cases with (red, *n* = 74) or without (black, *n* = 43) clustered-punctate polyGR+ staining. **e** Comparison of age at death of AD cases with (red, *n* = 79) or without (black, *n* = 52) clustered-punctate polyGR+ staining. **d**, **e** Statistical analysis was performed using Kaplan–Meier survival curve with Log-rank (Mantel–Cox) test

Further analyses of hippocampal polyGR+ aggregates in AD autopsy brains show four distinct staining patterns: (1) cytoplasmic polyGR (~ 88%) detected in all hippocampal subfields; (2) nuclear puncta polyGR+ staining (~ 4%) in the CA region; (3) polyGR+ staining in the DG region (~ 24%); (4) and polyGR+ aggregates with a clustered-punctate morphology (~ 59%) in the CA and subiculum regions. Examples of these polyGR+ staining patterns are shown in Fig. 2a–b and Fig. S8. In contrast to *C9orf72* ALS/FTD cases, polyGR+ aggregates are typically more frequent in AD autopsy brains (Fig. S9). Among the polyGR+ aggregate patterns, cytoplasmic polyGR+ staining is the most abundant and found across all hippocampal sub-regions. Other patterns of polyGR+ aggregates are found in autopsy brains from a smaller subset of AD cases. These polyGR+ aggregates can accumulate as large or small single aggregates in NeuN, Iba1, or GFAP-positive cells in CA and subiculum sub-regions of the hippocampus in AD autopsy brains (Fig. S10–12). In addition, co-staining shows the close-proximity regions of polyGR+ aggregates with a clustered-punctate morphology are positive for Aβ plaque, pTau, and LAMP1, which highlight dystrophic neurites (Figs. 2c and S13–15).

Next, we studied a subset of AD cases with extensive clinical data available from the combined cohort to better understand the relationship between polyGR+ aggregates and disease features (Table 1). The total levels of polyGR+ aggregates in hippocampal regions from AD autopsy brains showed no association with sex, age of onset, disease duration, or age of death (Fig. S16). However, disease onset of AD cases with clustered-punctate polyGR+ aggregates (*n* = 74) occurred significantly earlier with the mean age of onset of 68.5 years versus 76 years in AD cases without clustered-punctate polyGR+ staining (*n* = 43) (*p* = 0.0042) (Fig. 2d). In addition, the mean age at death of AD cases with clustered-punctate polyGR+ staining was earlier (78 years) (*n* = 79) compared with AD cases with other patterns of polyGR+ staining (81 years) (*n* = 52) (*p* = 0.0098) (Fig. 2e).

In summary, these results showed that polyGR+ aggregates accumulate in at least four distinct patterns in the hippocampus from AD autopsy brains. Patients with clustered-punctate polyGR+ staining that co-exist with the markers of dystrophic neurites had earlier ages of onset and ages of death than AD cases with other polyGR+ staining patterns. These data suggest that clustered-punctate polyGR+ aggregates are more toxic than other polyGR+ aggregate patterns.

### PolyGR+ aggregate levels are strongly associated with increased ADNC

Postmortem assessment of the severity of ADNC is based on the presence and distribution of Aβ plaques and pTau tangles [38, 39, 43, 82, 105]. Next, we studied whether polyGR+ aggregates correlate with these AD neuropathological hallmarks by performing IHC staining using sequential hippocampal sections from AD autopsy brains and antibodies targeting Aβ plaques (Ab5) or pTau (AT8, Ser202/Thr205) [9, 11, 54].

**Fig. 3:**
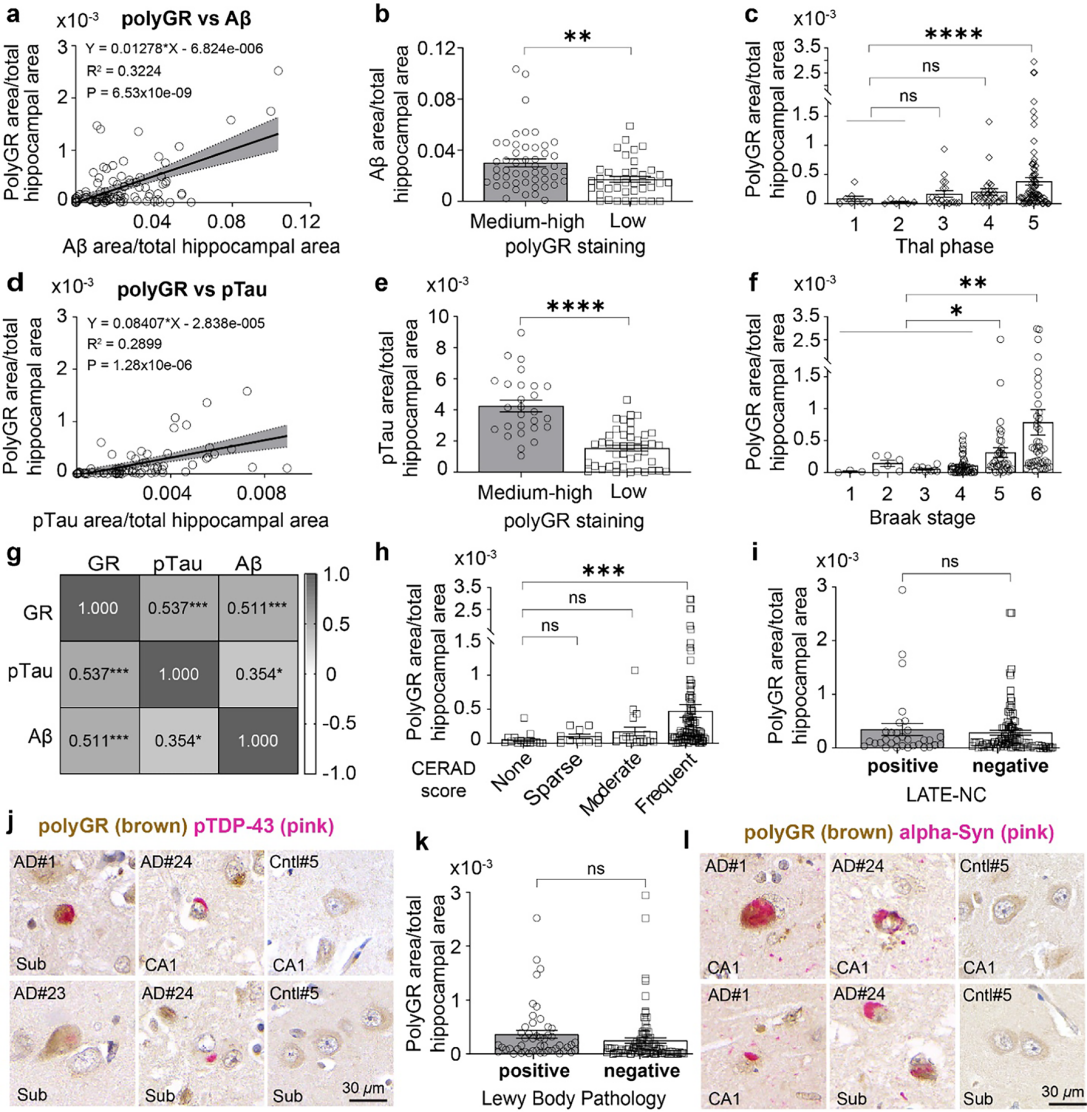
Levels of polyGR+ aggregates are correlated with neuropathological changes (Aβ and pTau) in AD autopsy brains. **a** Simple linear regression plot of polyGR+ aggregate and Aβ plaque levels in hippocampal (HC) sections of AD autopsy brains (*n* = 89), (95% CI [0.008572, 0.01656]). **b** Comparison of Aβ plaque levels in the HC of AD cases with medium-to-high polyGR (*n* = 51) vs those with lower polyGR aggregate levels (*n* = 38). **c** Plot of polyGR+ aggregate levels in AD cases with different Thal phases, phases 1 and 2: *n* = 14, phase 3: *n* = 19, phase 4: *n* = 26, phase 5: *n* = 81. **d** Simple linear regression plot of polyGR+ aggregate and pTau levels in the HC regions of AD autopsy brains (*n* = 71), (95% CI [0.05273, 0.1157]). **e** Comparison of pTau staining in the HC of AD cases with medium-to-high polyGR (*n* = 28) vs those with lower polyGR aggregate levels (*n* = 43). **f** Plot of polyGR+ staining levels in AD cases with different Braak stages, stages 1, 2, and 3: *n* = 19, stage 4: *n* = 45, stage 5: *n* = 37, stage 6: *n* = 46. **g** Heatmap showing correlation of polyGR, Aβ, and pTau levels in a sub-cohort of 43 AD cases that have data for all three stainings available. Heatmap matrix was generated by computing Pearson correlation coefficients. **h** Plot of polyGR+ aggregate levels in AD autopsy brains with different CERAD scores (none: *n* = 15, sparse: *n* = 10, moderate: *n* = 18, frequent: *n* = 105). **i** polyGR+ aggregate levels in AD brains with and without LATE-NC characterized by phosphorylated TDP-43 (pTDP-43) pathology (positive: *n* = 32, negative: *n* = 98). pTDP-43 was detected using an antibody against phosphorylated TDP-43 at S409/410. **j** IHC double staining showing LATE-NC (pTDP-43 inclusion) and polyGR+ aggregates co-exist in the same cells (AD#1 and AD#23, left panels) or are present in different cells (AD#24, middle panels) (Sub: Subiculum, CA: Cornu Ammonis). **k** PolyGR+ aggregate levels in AD autopsy brains with and without Lewy body pathology (LBP) (positive: *n* = 49, negative: *n* = 91). **l** IHC double staining showing LBP (α-syn inclusion) and polyGR+ aggregates co-exist in the same cells (AD#1—upper left panel and AD#24—both middle panels) or are present in different cells (AD#1—lower right panel and AD#24—lower middle panel). Data represent mean ± SEM. Statistical analyses were performed using unpaired two-tailed Welch’s *t* test (**b**, **e**, **i**, **k**) and one-way ANOVA with Brown–Forsythe test (**c**, **f**, **h**). Sex and gender were also taken into consideration with an ANCOVA analysis. ns *p* > 0.05, *p < 0.05, **p < 0.01, ***p < 0.001, ****p < 0.0001

Quantification and correlation analyses show polyGR+ aggregate levels are significantly associated with increased levels of Aβ plaques in the hippocampus of AD autopsy brains (*r* = 0.568, *R*^2^ = 0.3224, *p* = 6.53 × 10^−9^) (Fig. 3a). Using QuPath analyses, we established a threshold of polyGR+ staining levels that classifies the AD groups into minimal-to-low polyGR+ (low, polyGR area/total hippocampal area < 0.00010371) and medium-to-high polyGR+ AD cases (medium–high, polyGR area/total hippocampal area ≥ 0. 00010371). Aβ plaque levels are ~ 1.76-fold higher (*p* = 0.0013) in hippocampal regions from AD cases that have medium–high levels of polyGR+ staining compared to AD cases with low levels of polyGR+ staining (Fig. 3b). The spread of Aβ plaques starting in the neocortex to other regions in AD brains is categorized into five different Thal phases [100]. Our data show that hippocampal polyGR+ aggregate levels are significantly increased in AD autopsy brains with Thal V phase compared with those with pre-clinical Thal phases (phases 1, 2) (*p* = 0.000039) (Fig. 3c).

Similar to Aβ plaques and consistent with our previous findings [68], polyGR+ aggregate levels are positively associated with pTau [Ser202/Thr205] staining in hippocampal regions of AD autopsy brains (*r* = 0.538, *R*^2^ = 0.2899, *p* = 1.28 × 10^−6^) (Fig. 3d). Levels of pTau are ~ 2.76-fold higher in medium–high polyGR+ AD cases compared to low polyGR+ AD cases (*p* = 1.19 × 10^−7^) (Fig. 3e). We next examined if the deposition of neurofibrillary pTau tangles in different regions of AD brains, which is scored using six different Braak stages [9, 11], is associated with polyGR+ aggregate levels in the hippocampus. AD autopsy brains with Braak stages V and VI show increased hippocampal polyGR+ staining compared with those with lower Braak stages (I–IV) (*p* = 0.019 and 0.0024, respectively) (Fig. 3f).

To further understand the association of hippocampal polyGR+ aggregates with Aβ plaque and pTau levels, we performed a correlation analysis on 43 AD cases that have staining data available for all three pathological markers. The matrix heatmap shows polyGR+ staining is associated with increased staining of Aβ plaques (*r* = 0.511, *p* = 0.0005, 95% Cl (0.24939, 0.70368)) and pTau (*r* = 0.537, *p* = 0.0002, 95% Cl (0.2824, 0.72117)) at levels that are stronger than the correlation of Aβ with pTau (*r* = 0.354, *p* = 0.0198, 95% Cl (0.060293, 0.59162)) (Fig. 3g and Fig. S17). We next studied if polyGR+ aggregate levels correlate with the presence of neuritic plaques. Measured by a score derived from the Consortium to Establish a Registry for Alzheimer’s (CERAD), neuritic plaques are a subset of Aβ plaques surrounded by dystrophic neurites, pTau, and reactive gliosis [41, 42]. CERAD scores have been shown to be a better predictor of dementia progression compared to all forms of extracellular Aβ plaques [73, 92, 109]. Quantification shows a positive trend in polyGR+ aggregate levels in AD cases with CERAD scoring neuritic plaques from “none” to “sparse” to “moderate” and “frequent”, in which AD cases with CERAD scores of “frequent” showed significantly increased polyGR+ aggregate levels compared to those without neuritic plaques (CERAD scores = none) (*p* = 1.13 × 10^−4^) (Fig. 3h).

Increased phosphorylation and mislocalization of TAR DNA-binding protein 43 (TDP-43) are detected in a group of neurodegenerative diseases including ALS, FTD, HD, and AD [44, 58, 62, 69, 77, 99]. Limbic-predominant age-related TDP-43 encephalopathy neuropathological changes (LATE-NC) are characterized as a type of TDP-43 pathology in aging brains [45, 67]. C9-RAN polyGR proteins were shown to induce TDP-43 pathology in C9-ALS/FTD models [17, 86, 87, 95]. Thus, we studied if hippocampal polyGR+ aggregates are associated with LATE-NC in AD autopsy brains. We did not detect differences in the levels of hippocampal polyGR+ aggregates between AD cases with and without LATE-NC (Fig. 3i). IHC double staining shows polyGR+ aggregates and TDP-43 inclusions co-exist in the hippocampal sub-regions of a subset of AD autopsy brains (Fig. 3j and S18). In addition, double immunofluorescence staining shows polyGR+ aggregates and phosphorylated TDP-43 inclusions can be found in the same cells or different cells in hippocampal sections from AD autopsy brains with LATE-NC (Fig. S19).

In addition to LATE-NC characterized by TDP-43 pathology, Lewy bodies (LB) consisting of the deposition of misfolded α-synuclein (α-syn) have been implicated in a group of neurodegenerative diseases (synucleinopathies) including PD and Lewy body dementia (LBD) [12, 59]. Lewy bodies have been shown to co-exist with ADNC in AD autopsy brains [4, 37]. There is no difference in hippocampal polyGR+ aggregate levels between AD cases with and without the presence of Lewy body pathology (LBP) in our AD cohort (Fig. 3k). Interestingly, double IHC staining shows polyGR+ aggregates and α-syn inclusions can be co-found in a subset of cells in the hippocampus of AD autopsy brains with LDP (Figs. 3l and S20).

In summary, our results show that polyGR+ aggregates are strongly associated with increased levels of Aβ plaques and pTau tangles in the hippocampus of AD autopsy brains. Higher polyGR+ staining is found in AD autopsy brains with higher levels of Aβ and pTau spreading and increased frequency of neuritic plaques. In addition, polyGR+ aggregates co-exist with other neuropathological changes including LATE-NC and LDP in the hippocampus of a subset of AD autopsy brains.

### Association of hippocampal polyGR+ aggregate levels with known AD-risk genes

**Fig. 4:**
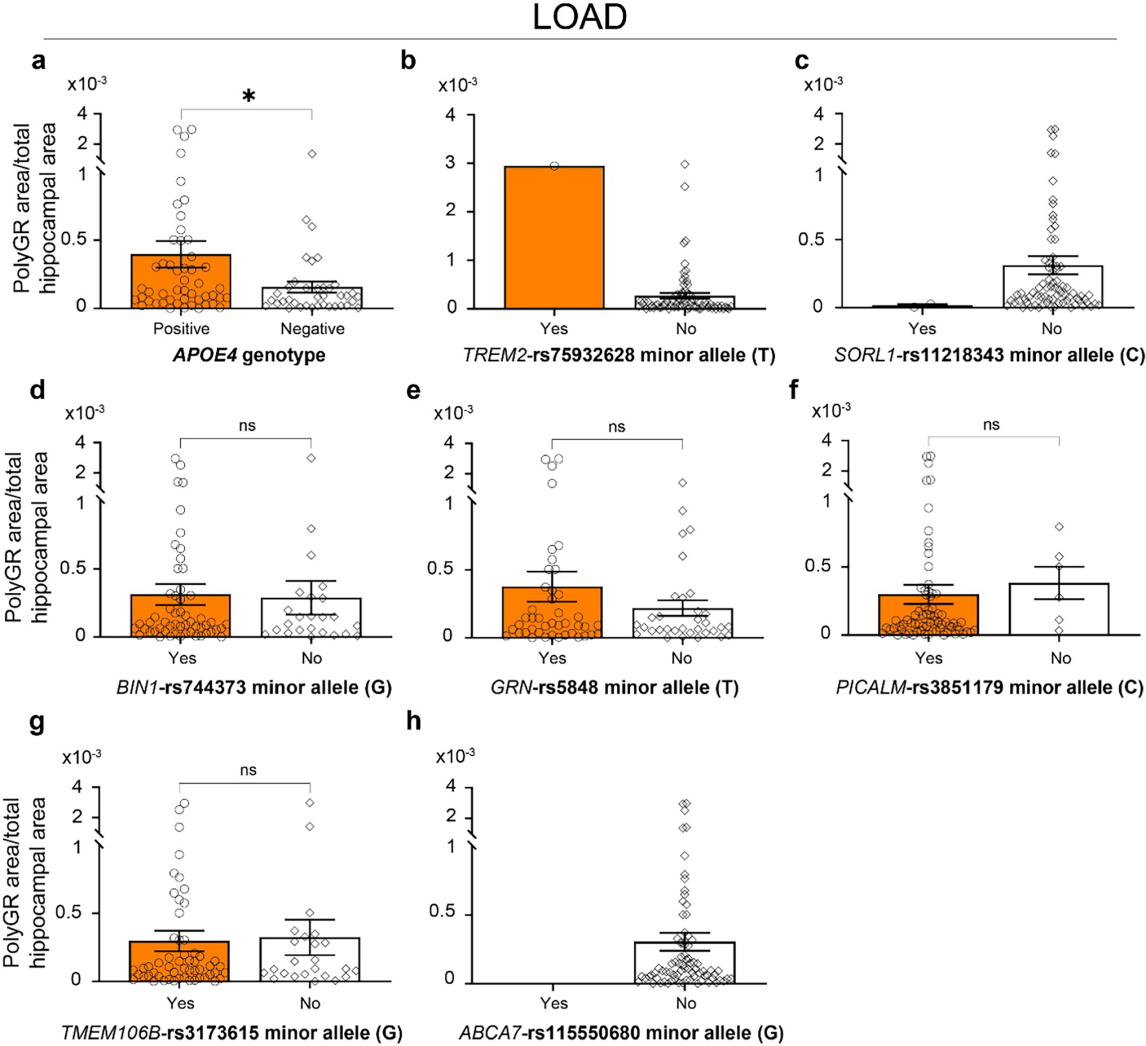
Association of polyGR+ aggregate levels with known AD-associated single nucleotide polymorphisms (SNPs). PolyGR+ aggregate levels in the hippocampus of LOAD cases with and without **a** *APOE4* allele(s) (positive *n* = 50, negative *n* = 38), **b** minor T allele of *TREM2*-rs75932628 (Yes = 1, No = 75), **c** minor C allele of *SORL1*-rs11218343 (Yes = 2, No = 77), **d** minor G allele of *BIN1*-rs744373 (Yes = 55, No = 24), **e** minor T allele of *GRN*-rs5848 (Yes = 43, No = 33), **f** minor C allele of *PICALM*-rs3851179 (Yes = 73, No = 6), **g** minor G allele of *TMEM106B*-rs3173615 (Yes = 55, No = 24), and **h** minor G allele of *ABCA7*-rs115550680 (Yes = 0, No = 79). Statistical analyses were performed using unpaired two-tailed Welch’s *t* test. Data represent mean ± SEM. ns p > 0.05, *p < 0.05

To study whether polyGR+ aggregates are associated with known AD-risk loci, we next performed genotype experiments for nine known AD-associated single nucleotide polymorphisms (SNPs), including rs429358 (*APOE4*) and rs7412 (*APOE2*), rs744373 (*BIN1)*, rs5848 (*GRN*), rs3851179 (*PICALM*), rs11218343 (*SORL1*), rs3173615 (*TMEM106B*), rs115550680 (*ABCA7*) and rs75932628 (*TREM2*) (summarized in Table 2 and Table S1). Previous studies showed that the T alleles of rs7412 encoding for *APOE2*, the C allele of *SORL1*-rs11218343 and the G allele of *TMEM106B-*rs3173615 were linked with a decreased risk of developing AD [48, 93]. In contrast, the other six minor alleles of rs429358 (C) encoding for *APOE4*, rs744373 (G) on *BIN1*, rs5848 (T) on *GRN*, rs3851179 (C) on *PICALM*, rs115550680 (G) on *ABCA7* and rs75932628 (T) on *TREM2* are associated with an increased risk of developing AD [25, 34, 51, 78, 81, 101, 106]. Our data show a ~ 2.5-fold increase (*p* = 0.025) in polyGR+ aggregate staining in the hippocampus of LOAD cases carrying *APOE4* allele(s) (Fig. 4a). This difference is not observed in EOAD cases positive for the *APOE4* allele (Fig. S21). We did not detect an association between polyGR+ aggregate levels and *APOE* alleles in the combined cohort of both EOAD and LOAD cases (Fig. S22). In addition, we also detected a LOAD case carrying a risk allele of *TREM2* with relatively high levels of hippocampal polyGR+ aggregates (Fig. 4b). In contrast, two LOAD cases that carry protective alleles of *SORL1* showed remarkably lower levels of hippocampal polyGR+ aggregates (Fig. 4c). No difference in polyGR aggregate levels was detected in the hippocampus of AD cases with *BIN*-rs7444373, *GRN*-rs5848, *PICALM*-rs3851179, or *TMEM106B*-rs3173615 when compared to the negative carriers (Fig. 4c–g), and no rare mutation of *ABCA7*-rs115550680 minor allele (G) was detected in our cohort. In summary, these results suggest that polyGR+ staining in the hippocampus of AD autopsy brains is positively associated with AD-risk *APOE4* alleles.

### PolyGR+ aggregate levels are increased in autopsy brains from late-onset AD cases with a history of stroke or high blood pressure

Epidemiological studies have identified non-genetic risk factors that may contribute to AD and disease pathology [70, 72, 108], so we examined whether polyGR+ aggregates correlate with non-genetic AD-risk factors or comorbidities. For cases with clinical information available, approximately 17.7% (22/124), 8.1% (10/124), and 63.7% (79/124) of AD cases in our cohort have a history of traumatic brain injury (TBI), stroke, and high blood pressure (HBP), respectively. The inclusion criteria were gathered from clinical reports, and the information is summarized in Table 3 and Table S2.

**Table 3:** Information of comorbidities of AD cases

Comorbidities	Combined	Control/PART	AD low	AD intermediate	AD high	AD
Traumatic brain injury (TBI)	22	–	2	8	12	0
Stroke	10	–	2	1	7	0
Concussion	8	–	0	3	4	1
Transient ischemic attack (TIA)	10	–	2	3	5	0
Stroke or TIA (Unclear)	13	–	5	2	4	0
No TBI/stroke/TIA	71	–	10	23	35	3
High blood pressure (HBP)						
Yes	79	–	12	20	32	2
No	45	–	4	10	12	2

*TBI* traumatic brain injury clinical information reports severe head injury, *TIA* transient ischemic attack. Comorbidity information was collected based on clinical assessment and medical record

**Fig. 5:**
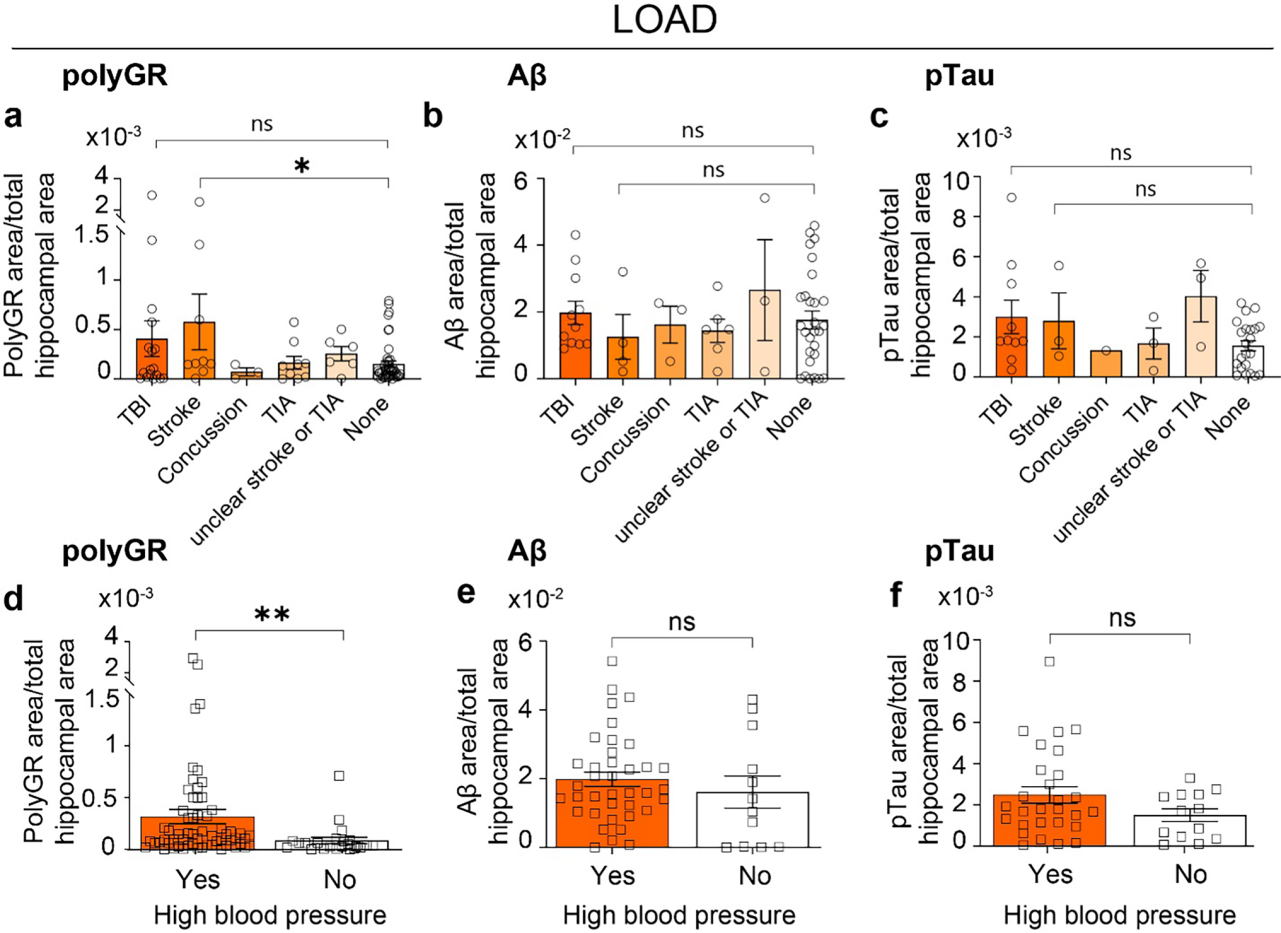
Hippocampal polyGR+ aggregate levels are increased in late-onset AD (LOAD) cases with stroke or high blood pressure. **a**–**c** Plots showing levels of hippocampal polyGR+ aggregates **a** (*n* = 84), Aβ plaques **b** (*n* = 50), and pTau **c** (*n* = 69) in LOAD cases with or without a history of TBI, stroke, concussion, or transient ischemic attack (TIA). Statistical analyses were performed using ordinary one-way ANOVA with the Sidak test. **d**–**f** Plots showing levels of hippocampal polyGR+ aggregates **d** (*n* = 84), Aβ plaques **e** (*n* = 50), and pTau **f** (*n* = 41) in LOAD cases with or without a history of high blood pressure. Statistical analyses were performed using an unpaired two-tailed Welch’s *t* test. Sex and gender were also taken into consideration with an ANCOVA analysis. Data represent mean ± SEM. ns *p* > 0.05, *p < 0.05, **p < 0.01

Analysis of AD cases with or without a history of brain injuries, including TBI, stroke, concussion, TIA and unclear stroke, or TIA, showed no differences in polyGR+ aggregate, Aβ, or pTau levels in hippocampal regions of early-onset AD (EAOD) autopsy brains (Fig. S23a–c). In contrast, levels of hippocampal polyGR+ aggregates were ~ 3.8-fold higher in late-onset AD (LOAD) cases with a history of stroke (*p* = 0.022) compared to cases with no brain injury history (Fig. 5a). Further analysis that considers age at death as a possible confounding variable shows that age at death did not contribute to the difference in polyGR+ aggregate levels between AD cases with a history of stroke and those without a history of brain injuries (Fig. S24a). In a subset of LOAD cases with a history of brain injuries and available data for pTau, or Aβ plaque staining, no differences in Aβ plaque or pTau levels were detected between AD cases with or without brain injuries (Fig. 5b and c). To further study the correlation of stroke and polyGR+ aggregate staining, we performed polyGR IHC staining on three brain regions with characterized infarcts from two AD cases from the 1Florida-ADRC/UF-HBTB brain bank. PolyGR+ staining is detected in the examined brain regions with infarcts (Fig. S25).

In addition, our results show a ~ 3.71-fold increase in hippocampal polyGR+ staining in LOAD cases with high blood pressure (HBP) when compared to cases without HBP (*p* = 0.0031) (Fig. 5d). In contrast, we did not detect a difference in polyGR+ aggregate, Aβ, or pTau levels in the hippocampus of autopsy brains from early-onset AD (EAOD) autopsy brains with or without HBP (Fig. S23d, e, and f). No difference in age at death between LOAD cases with or without HBP (Fig. S24b) demonstrates that the increased levels of hippocampal polyGR+ aggregates in LOAD cases with HBP are not linked with age. In contrast, there is no difference in hippocampal Aβ plaque or pTau levels between LOAD cases with or without a history of HBP (Fig. 5e and f). No association of high cholesterol, heart disease, heart attacks, depression/anxiety, cancer diabetes, joint diseases, cerebral amyloid angiopathy (CAA), a family history of dementia, or loss of consciousness was observed with levels of polyGR+ aggregate staining in the hippocampus of AD autopsy brains in our cohort (Fig. S26).

In summary, our data show that increased levels of polyGR+ aggregates in the hippocampus of AD autopsy brains are found in patients with a history of stroke or high blood pressure.

### Oxidative stress increases polyGR+ protein levels and polyGR-related pTau accumulation

**Fig. 6:**
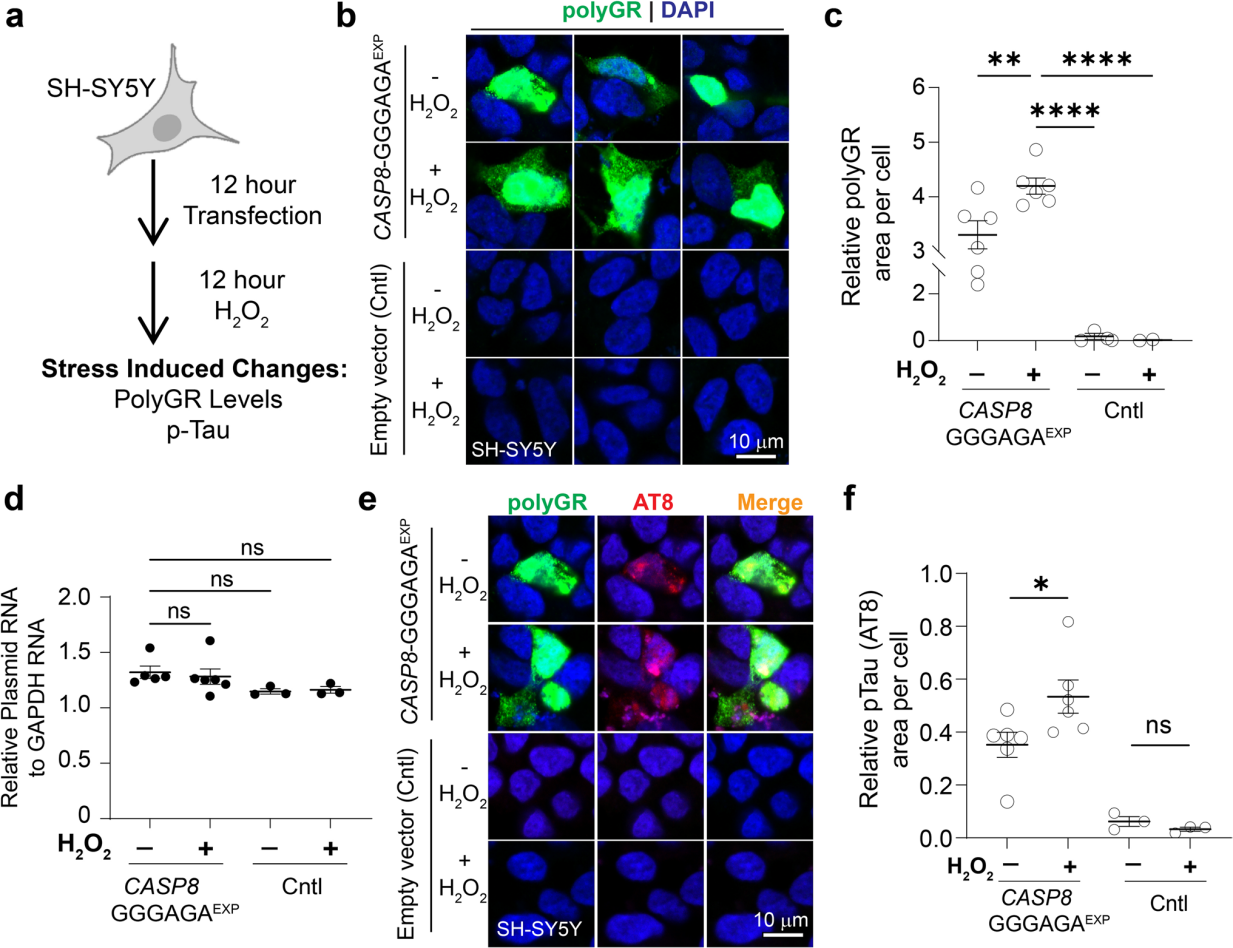
Oxidative stress increases polyGR+ protein levels and polyGR-related pTau accumulation. **a** Schematic diagram showing an experimental workflow to study the effects of stress induced by hydrogen peroxide (H_2_O_2_) on polyGR+ and pTau levels in transfected SH-SY5Y cells. **b** Representative immunofluorescence images of polyGR+ protein staining in SH-SY5Y cells transfected with *CASP8*-GGGAGA^EXP^ expressing plasmids (p-AD-R1 or p–C-Var shown in Fig. 6A Nguyen et al., *PNAS*) or empty vector control plasmids and then treated with or without hydrogen peroxide (H_2_O_2_, 50 µM). **c** Quantification of polyGR+ protein levels in SH-SY5Y cells transfected with *CASP8*-GGGAGA^EXP^ and empty vector control plasmids (Cntl), with or without H_2_O_2_ treatment. **d** Plot showing plasmid RNA levels normalized to GAPDH in transfected SH-SY5Y cells with or without H_2_O_2_ treatment. **e** Representative images of polyGR and pTau (AT8, S202/T205) staining in SH-SY5Y cells that were transfected with *CASP8*-GGGAGA^EXP^ or empty vector control (Cntl) plasmids and then treated or not treated with H_2_O_2_. **f** Quantification of pTau levels in SH-SY5Y cells that were transfected with *CASP8*-GGGAGA^EXP^ or empty vector control plasmids and then were treated or not treated with H_2_O_2_. Statistical analyses were performed using one-way ANOVA with Tukey’s multiple comparisons (**c**, **d**) or using unpaired two-tailed Welch’s *t* test (**f**). Data represent mean ± SEM. ns p > 0.05, *p < 0.05, **p < 0.01, ***p < 0.001, ****p < 0.0001

The correlation of polyGR+ aggregate levels and history of stroke or HBP in AD patients suggests that oxidative stress induced by stroke [2, 46, 75] and HBP [7, 36] may favor the expression and accumulation of polyGR proteins in AD brains. We previously showed that a subset of polyGR+ proteins that accumulate in AD autopsy brains is caused by the GGGAGA repeat expansion within an SVA element in *CASP8* (*CASP8*-GGGAGA^EXP^) using C-terminal antibodies specific for the *CASP8* polyGR+ proteins [68]. Consistently, hybridization chain reaction fluorescence in situ hybridization (HCR-FISH) detects *CASP8*-GGGAGA^EXP^ transcript staining in the hippocampus from *CASP8*-GGGAGA^EXP^ (+) and polyGR+ AD autopsy brains but not AD brains that are positive for polyGR+ aggregates and negative for the *CASP8*-GGGAGA^EXP^ (Fig. S27). The integrated stress response (ISR) was previously shown to increase levels of polymeric RAN proteins in several repeat expansion disorders [14, 31, 66, 107, 119]. These studies combined with our stroke and HBP data suggested to us that the expression of GGGAGA-encoded polyGR+ protein levels increases with oxidative stress. To test this hypothesis, SH-SY5Y cells transfected with *CASP8*-GGGAGA^EXP^ minigenes (p-AD-R1 and p-C-Var plasmids in Nguyen et al. [68]) or empty vector control plasmids were treated with or without hydrogen peroxide (H_2_O_2_), which induces reactive oxygen species to mimic cellular oxidative stress (Fig. 6a) [14, 79, 111]. PolyGR+ protein signal in H_2_O_2_-treated and untreated cells was quantified using QuPath. H_2_O_2_ treatment resulted in a ~ 27% increase in polyGR+ protein levels in *CASP8*-GGGAGA^EXP^ plasmids transfected SH-SY5Y cells compared to untreated cells (*p* = 0.0097) (Figs. 6b, c, and S28). No similar polyGR+ staining was detected in SH-SY5Y cells transfected with empty vector control plasmids with and without H_2_O_2_ treatment (Figs. 6b, c, and S28). In addition, we did not detect differences in plasmid RNA levels between the untreated and H_2_O_2_-treated groups (Fig. 6d).

Next, we tested if oxidative stress induced by H_2_O_2_ also increases tau phosphorylation induced by polyGR+ proteins. Consistent with our previous study [68], pTau [S202/T205] levels are increased in SH-SY5Y cells transfected with *CASP8*-GGGAGA^EXP^ plasmids (p-AD-R1 and p-C-Var constructs, Fig. 6A in Nguyen et al. [68]) compared to empty vector control plasmids (Fig. S29). Cells transfected with the *CASP8*-GGGAGA^EXP^ plasmids showed a ~ 51% increase in pTau [S202/T205] when treated with H_2_O_2_ compared to untreated cells (*p* = 0.033) (Figs. 6e, f, and S30a). In contrast, H_2_O_2_ treatment did not increase pTau [S202/T205] levels in SH-SHY5 cells transfected with empty vector control plasmids (Figs. 6e, f, and S30b).

In summary, these data combined with the increased levels of polyGR+ aggregates in patients with stroke or high blood pressure (Fig. 5) suggest a model in which oxidative stress increases polyGR+ aggregate levels including polyGR+ proteins expressed from the *CASP8* repeat expansion mutation, and that increased levels of polyGR+ subsequently lead to pTau pathology.

## Discussion

We show that polyGR+ aggregates are frequently detected in the hippocampal regions of three enlarged cohorts of AD autopsy brains, but not in age-similar controls or PART cases, which have pTau but not or minimal Aβ pathology. PolyGR+ aggregates accumulate in at least four patterns, including cytoplasmic, DG staining, nuclear staining, and clustered-punctate patterns, which are distinct from polyGR staining in C9-ALS/FTD autopsy brain tissue. Interestingly, clustered-punctate polyGR+ aggregates are positive for the markers of dystrophic neurites and are associated with earlier age of onset and shortened lifespan in AD cases. In the hippocampus, increased levels of polyGR+ aggregates are detected in AD autopsy brains with increased levels of Aβ plaques and pTau [S202/T205]. We also show hippocampal polyGR+ aggregates are associated with increased deposition of Aβ plaques and pTau lesions and frequency of neuritic plaques in AD brains measured by Thal phases, Braak stages, and CERAD scores, respectively. In addition, an increase in polyGR+ aggregate levels is detected in autopsy brain tissue from AD individuals who experienced stroke or high blood pressure (HBP). To further study the possible effects of oxidative stress on molecular changes linked with stroke or HBP on polyGR+ protein levels, we tested the effects of oxidative stress on cellular models of polyGR+ proteins expressed by *CASP8*-GGGAGA repeat expansion variants, which were previously shown to produce polyGR-rich proteins in AD autopsy brains [68]. Oxidative stress induced by H_2_O_2_ treatment, which mimics molecular changes linked with stroke or high blood pressure [2, 7, 36, 46, 75], increases levels of *CASP8* polyGR+ proteins in transfected SH-SY5Y cells. In addition, we show that the accumulation of *CASP8* polyGR+ proteins but not H_2_O_2_ treatment alone leads to increased pTau. Taken together, these results suggest a molecular connection between the accumulation of polyGR+ aggregates, stress induced by non-genetic AD-risk factors, and the risk of developing AD.

The accumulation of polyGR proteins was first described in autopsy tissue samples from C9-ALS/FTD patients [5, 65, 120]. Among six different dipeptide RAN proteins expressed by *C9orf72* G4C2•C4G2 repeat expansions, polyGR aggregates were shown to correlate with neurodegeneration and pathological subtypes in C9-ALD/FTD patient brains [88]. In AD autopsy brains, we detect at least four different accumulation patterns of polyGR+ aggregates and show that polyGR+ staining with clustered-punctate morphology correlates with earlier onset and decreased survival of AD patients. These results suggest that different patterns of polyGR+ protein accumulation have different levels of toxicity, which could contribute to disease in distinct manners. It is possible that clustered-punctate polyGR+ aggregates co-aggregate with other proteins, which could be more immunogenic and have increased toxicity compared to other forms of polyGR+ aggregates. Future studies will be needed to understand whether the formation of clustered-punctate polyGR+ proteins is caused by specific microenvironment changes within those AD brains and/or due to distinct biochemical properties of polyGR+ protein species expressed from different repetitive sequences in patient genomes [35, 68] and/or specific cellular subtypes [110, 112].

We did not detect a clear difference in protein signal using the previously developed α-polyGR antibody [120], western blots and denatured proteins from soluble and 2% SDS protein fractions extracted from frozen frontal cortex tissue from a subset of AD, control, and PART cases. However, our data suggest there could be stronger signal above the range of molecular weights larger than 100 KDa for the 2% SDS protein fractions from AD cases. It has been described that antibodies could have different levels of performance across different immunoassays [103]. The α-polyGR antibody was originally shown to detect epitope tagged polyGR proteins expressed by IF and protein plotting in transfected cells and specific perinuclear aggregates in autopsy tissue from C9-ALS/FTD cases but not controls [120]. Consistently, we detected polyGR+ staining in autopsy brains from C9-ALS/FTD and AD cases but not controls. These data combined with our western blot results suggest that the α-polyGR antibody [120] works well with immunohistochemical staining but not in western blotting for detecting denatured polyGR+ proteins in protein lysate from brain autopsy tissue samples under our testing conditions. It is also possible that denaturation conditions in western blotting experiments altered target epitopes and exposed unwanted epitopes, which contributed to the signal we detected. In addition, various polyGR+ protein species could be produced by different genetic loci and translation start points, which could make the detection of polyGR+ proteins on western blot more challenging. To the best of our knowledge, to date, there was only one report of the detection of polymeric RAN proteins from protein lysates extracted from patient brain tissue using Western blotting [102]. In the future, the development of new α-polyGR antibodies that work in various immunoassays could facilitate research to better understand the accumulation of polyGR+ proteins in AD and open an opportunity for biomarker development.

While AD is characterized by the accumulation of Aβ plaques and neurofibrillary tangles composed of pTau proteins, the underlying causes and downstream consequences of these neuropathologies in most AD cases are still poorly understood. We show levels of polyGR+ aggregates are strongly associated with increased levels of Aβ plaques and pTau [S202/T205] in AD hippocampal regions. In addition, higher polyGR+ aggregate levels in the hippocampus are detected in AD autopsy brains with increased spreading of Aβ and pTau and elevated levels of neuritic plaques, which are established indicators of disease severity [10, 33, 57]. The strong association of polyGR+ aggregates with disease hallmarks suggests that polyGR pathology could play a role in the development of classic ADNC and disease progression. It would be interesting in future studies to investigate if polyGR+ proteins are present in patient bio-fluids that could serve as biomarkers of disease.

Aβ species have been shown to accumulate in AD brains as early as ~ 20–25 years prior to the first clinical symptoms manifest in AD patients [22]. We show polyGR+ aggregates are strongly associated with Aβ plaque staining in the hippocampal regions of AD brains. The accumulation of Aβ species may create a stressed environment that favors the production and/or aggregation of polyGR+ proteins over time, which could lead to a positive feedback loop that worsens Aβ pathology and/or induces toxic forms of Aβ plaques. Future work is needed to study if polyGR+ proteins expressed from the *CASP8*-GGGAGA^EXP^ and other genetic sources alter APP processing and Tau processing and phosphorylation or contribute to the accumulation of Aβ and pTau species in other disease models.

Among the tested brain regions, we find polyGR+ aggregates profoundly accumulate in the hippocampus of AD autopsy brains. In contrast, polyGR+ aggregates appear rarely to occasionally in the AD frontal cortex, pons, occipital, and cerebellum under our testing conditions. These results suggest region or cell type-specific effects of polyGR+ aggregate accumulation. Disease models that allow longitudinal studies would provide insights into the accumulation of polyGR+ aggregates in different brain regions and their effects on disease progress. In addition, it is possible that polyGR-containing neurons in the brain regions outside of the hippocampus are prone to degenerate early and are missed in the end-stage tissue or polyGR+ aggregates in those regions are more challenging to detect due to a different tissue background.

We did not detect differences in polyGR+ staining levels in the hippocampus of AD cases with or without LATE-NC or LBP. Since the LATE-NC associated with LATE is a common disease in 80+ year-old elderly individuals [44], and the median age of death of our AD cohort is 77.3 years, it would be interesting in the future to study if polyGR+ aggregates and LATE-NC show any association in older AD cohorts. The accumulation of polyGR proteins was previously shown to induce TDP-43 pathology in C9-ALS/FTD models [17, 86, 95]. In our cohort, TDP-43 inclusions were detected in a small subset of polyGR+ cells in the hippocampus of AD cases. In AD autopsy brains, polyGR+ aggregates show overlapping and distinct morphologies compared to polyGR aggregates in C9-ALS/FTD brains. PolyGR+ proteins that accumulate in AD brains may have distinct biochemical properties compared to C9-ALS/FTD RAN polyGR proteins. Supporting this hypothesis, a subset of polyGR+ aggregates in AD autopsy brains is produced by the *CASP8*-GGGAGA^EXP^, which is predicted to produce hybrid RAN proteins containing stretches of polyGR, polyRE, and polyGE. It is also possible that polyGR+ protein species produced by different genetic mutations could have differential effects on LATE-NC. In addition, polyGR+ aggregates and α-syn inclusions were found in the same cells in a subset of hippocampal neurons from AD brains. Future studies could also expand this analysis to a larger cohort of AD cases, which would facilitate studies to assess if polyGR and LBP pathologies are associated and/or if interactions between these two proteins influence disease features.

Association studies of polyGR+ aggregates with known AD genetic loci in our cohort showed higher levels of hippocampal polyGR+ aggregates in LOAD autopsy brains carrying *APOE4* alleles but not *BIN1*, *PICALM*, *TMEM106B*, or *GRN* risk SNPs. These results suggest an interaction of *APOE4* alleles and polyGR+ protein accumulation and/or their links with disease severity. Although AD-risk alleles of *TREM2* are rarely found in our cohort, we detected one LOAD case with *TREM2* with a high level of polyGR+ aggregates. In contrast, polyGR+ aggregate levels in two LOAD cases that carry protective alleles of *SORL1* are remarkably low compared to other AD cases. Combined, these results suggest future studies with a larger cohort of AD to study the association of polyGR+ aggregates with known AD-associated loci.

Our previous and current studies show that the expression of polyGR+ protein species produced by the *CASP8*-GGGAGA^EXP^ leads to increased pTau [S202/T205] in polyGR+ cells [68]. Oxidative stress induced by H_2_O_2_ treatment favors the accumulation of *CASP8* polyGR+ proteins and pTau increased by *CASP8* polyGR+ proteins. These results are consistent with the previous findings on the effects of stress conditions on the accumulation of polymeric RAN proteins in other repeat expansion disorders [14, 31, 66, 107, 119]. In addition, these results suggest that oxidative stress exacerbates molecular changes caused by the *CASP8*-GGGAGA^EXP^ including the accumulation of polyGR+ proteins and pTau induced by polyGR+ proteins, which could in turn contribute to these pathologies in AD.

Stroke [40, 49, 104] and high blood pressure [52, 85, 97] have been reported as non-genetic risk factors for dementia and AD. Interestingly, our results show hippocampal polyGR+ aggregate levels are increased in AD cases that have experienced a stroke when compared to cases with no reported history of brain injuries. High blood pressure can affect brain functions in several ways, including reducing blood flow to the brain, causing arteriolosclerosis, and increasing the risk of stroke, all of which can lead to brain damage and ultimately result in cognitive decline [16, 74]. Hippocampal polyGR+ aggregate levels are also increased in AD cases with high blood pressure. This striking increase in polyGR+ aggregate levels may be due to the conditions that favor the accumulation of polyGR+ proteins. Consistent with this hypothesis, we show that oxidative stress, which can be induced by stroke or high blood pressure [2, 7, 36, 46, 75], causes increased levels of *CASP8* polyGR+ proteins in transfected cells. In the future, it will be important to extend the analysis of brain injuries and high blood pressure and other genetic and non-genetic factors in relation to polyGR+ aggregates using additional AD cohorts that include extensive clinical data. Additional studies using animal models and larger cohorts of AD cases are also warranted to better understand the impact of brain injuries or HBP on the accumulation of polyGR+ proteins and their consequences on learning and memory.

In conclusion, our results demonstrate that polyGR+ aggregates are an important proteinopathy, which is strongly associated with the severity of ADNC and correlates with *APOE4* alleles, a history of stroke or high blood pressure in AD patients. In vitro studies show oxidative stress favors the accumulation of polyGR+ proteins, which in turn increases the levels of pTau in cells. Taken together, these results suggest a molecular link between brain injuries, high blood pressure, and polyGR+ RAN proteins and increased AD risk.

## Supplementary Information

Below is the link to the electronic supplementary material.

Supplementary file1 (DOCX 15832 KB)

Supplementary file2 (DOCX 15669 KB)

Supplementary file3 (DOCX 5198 KB)
