# Supplementary material for: PolyGR-containing aggregates link with pathology and clinical features of Alzheimer’s disease
Source: Acta Neuropathol. 2025 Nov 8;150(1):49. doi: 10.1007/s00401-025-02954-8 (PMC12598690; doi:10.1007/s00401-025-02954-8)
Supplement: Supplementary file 1 — Supplementary file1 (DOCX 15832 KB) [file 401_2025_2954_MOESM1_ESM.docx]

**polyGR-containing aggregates link with pathology and clinical features of Alzheimer’s disease**

Huong T. Phuong^1, 2 #^, Rodrigo F. Tomas^1, 2 #^, Cemal Akmese^1^, Ana Mijares^1, 2^, Isabella M. Gerstin^1, 2^, Shu Guo^1, 2^, Logan R. Bell^1, 2^, Ross Ellwood^1, 2^, Svitlana Yegorova^1, 2^, Stefani K. Ng^1^, Grace Massey^1^, Jennifer Phillips^3, 4^, Alexandra Melloni^5^, Olga Pletnikova^6^, XiangYang Lou^7^, H. Brent Clark^8^, Juan C. Troncoso^6^, Bradley Hyman^5^, Stefan Prokop^3, 4, 9, 10, 11^, Laura P. W. Ranum^1, 2, 9, 10, 11, 12^, Lien Nguyen^1, 2, 9, 11, 12^*

^1^Center for NeuroGenetics, College of Medicine, University of Florida; ^2^Department of Molecular Genetics & Microbiology, College of Medicine; University of Florida; ^3^Department of Pathology, Immunology and Laboratory Medicine, College of Medicine, University of Florida; ^4^Center for Translation Research in Neurodegenerative Disease, College of Medicine University of Florida; ^5^MassGeneral Institute for Neurodegenerative Disease, Massachusetts General Hospital; ^6^Department of Pathology, The Johns Hopkins University School of Medicine; ^7^Department of Biostatistics, University of Florida; ^8^Department of Laboratory Medicine and Pathology, University of Minnesota; ^9^McKnight Brain Institute, University of Florida; ^10^Department of Neurology, College of Medicine, University of Florida; ^11^Norman Fixel Institute for Neurological Disease, University of Florida; ^12^Genetics Institute, University of Florida.

^#^ Authors contribute equally to this work

* Corresponding author: Lien Nguyen (lien.nguyen@ufl.edu)

**Supplemental figures and tables**

**α-polyGR+ staining in Hippocampus**

**
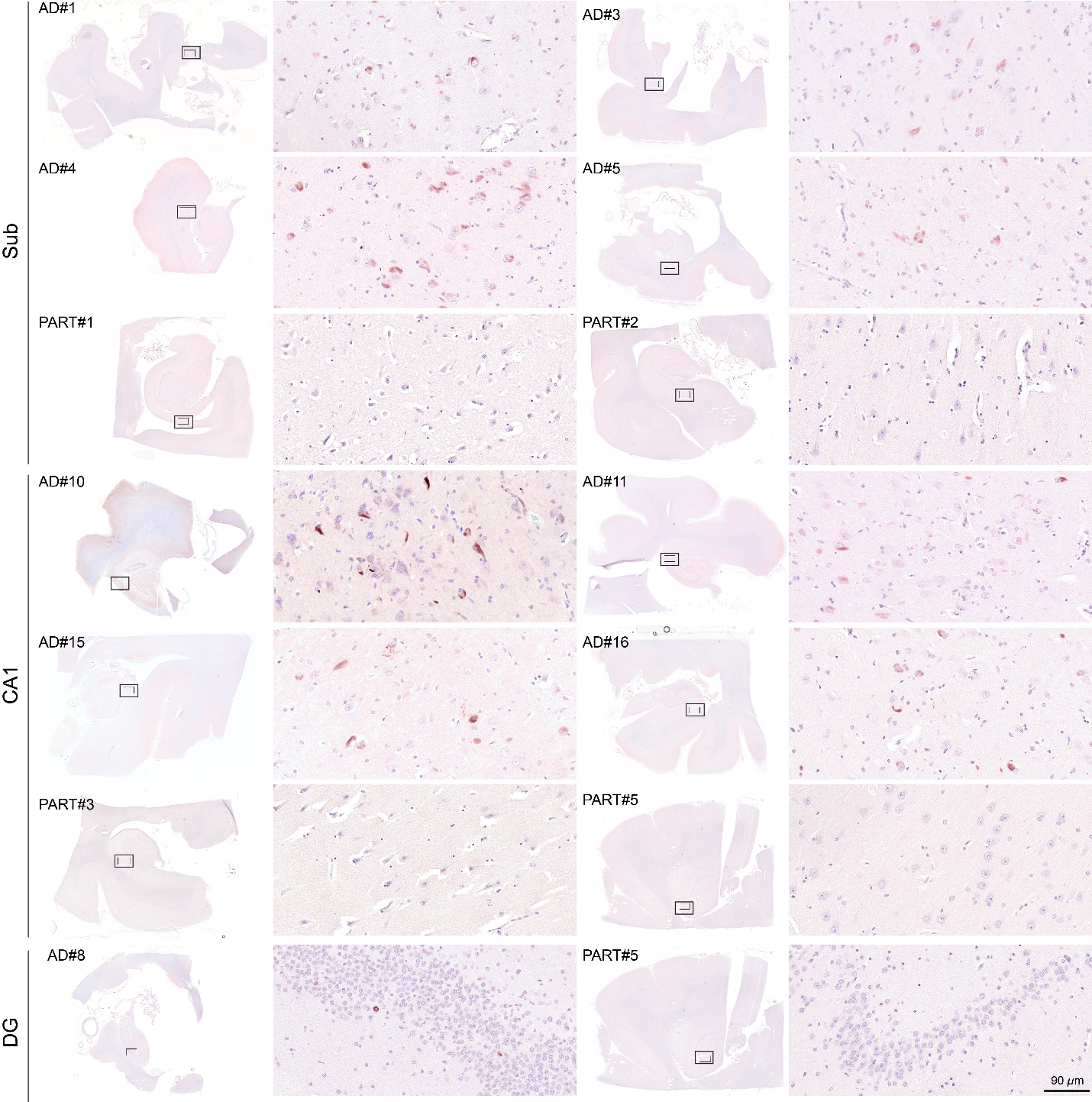
**

**Figure S1. Widefield and zoomed-in immunohistochemical (IHC) images of polyGR+ staining in hippocampal regions from AD and control autopsy brains.** PolyGR+ staining is red. PART: Primary Age-Related Tauopathy, Sub: Subiculum, CA: Cornu Ammonis and DG: Dentate Gyrus

**α-polyGR+ staining in Occipital Cortex**


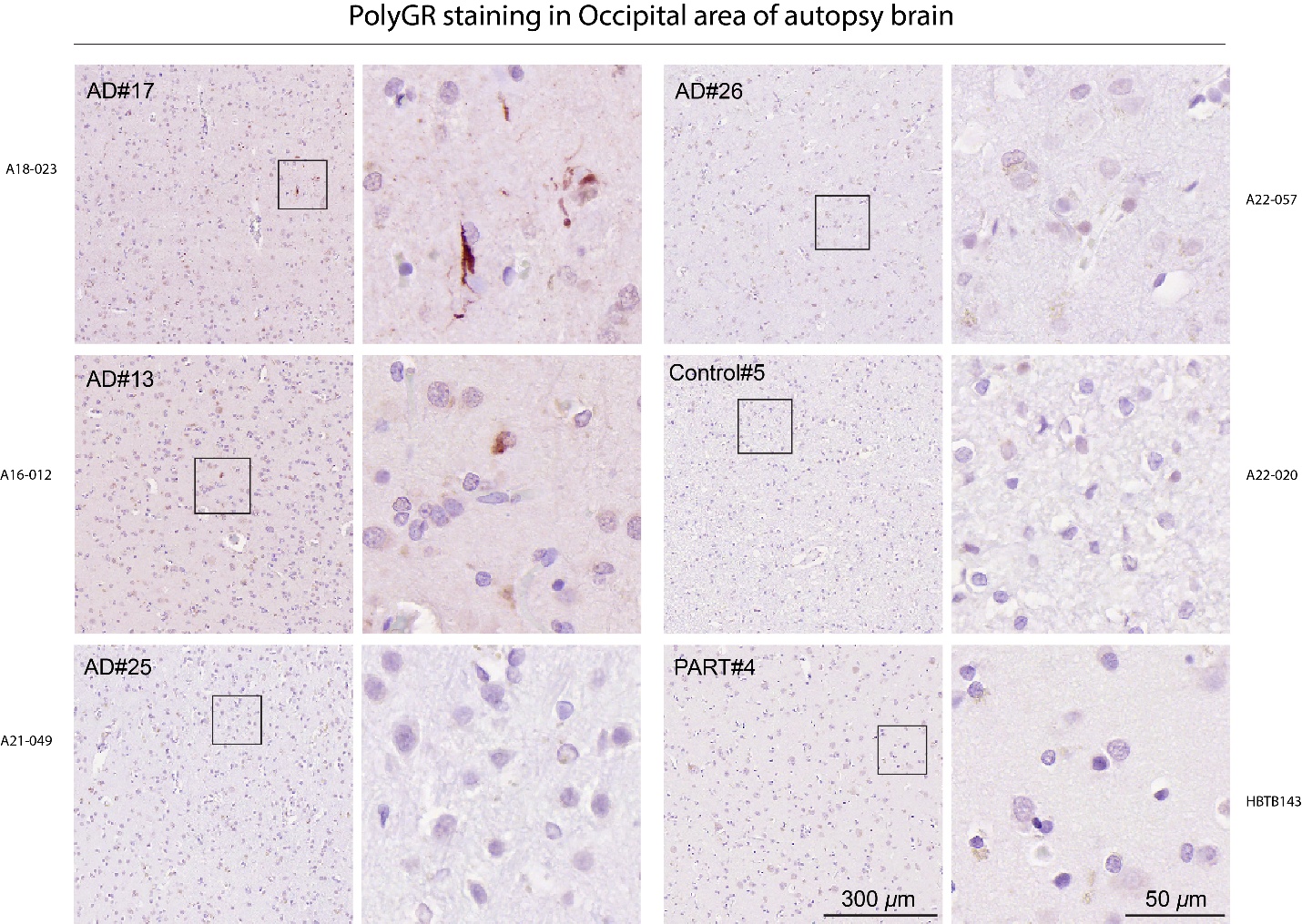


**Figure S2. Widefield and zoomed-in IHC images of polyGR+ in staining in the occipital cortex from AD and control autopsy brains.** PolyGR+ staining (red) was detected by using the same IHC staining protocol in hippocampus. The right side of each case is zoomed out from squared area. PART: Primary Age-Related Tauopathy.

**α-polyGR+ staining in Medulla oblongata**


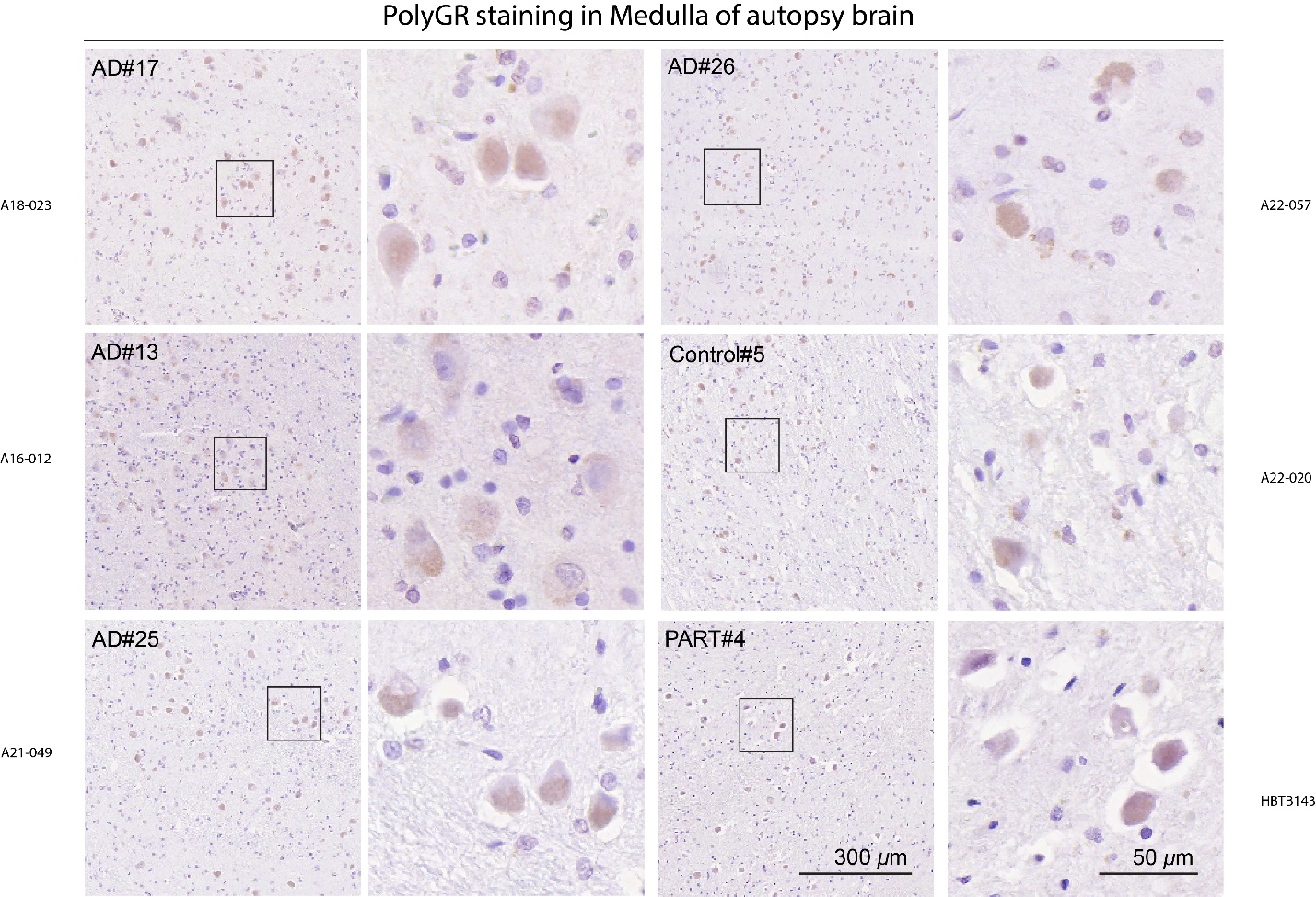


**Figure S3. Widefield and zoomed-in IHC images of polyGR+ staining in medulla oblongata sections from AD and control autopsy brains.** PolyGR+ staining was detected by using the same IHC staining protocol in hippocampus. The results show polyGR+ staining was not detected in medulla from AD autopsy brains under testing conditions. The right side of each case is zoomed out from squared area. PART: Primary Age-Related Tauopathy.

**α-polyGR+ staining in Pons**


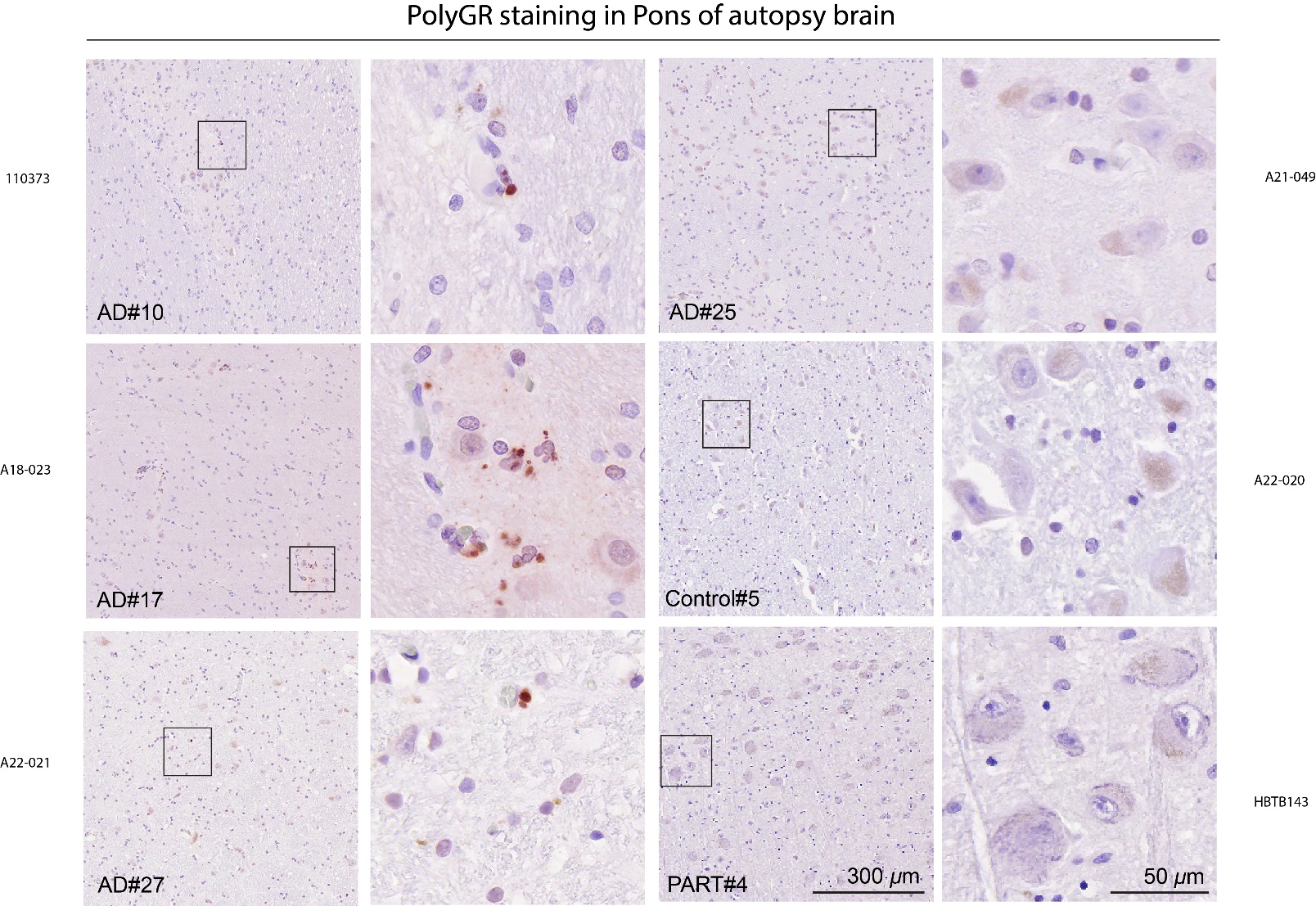


**Figure S4. Widefield and zoomed in IHC images of polyGR+ staining in the pons regions from AD and control autopsy brains.** PolyGR+ staining (red) was detected by using the same IHC staining protocol in hippocampus. The right side of each case is zoomed out from squared area. PART: Primary Age-Related Tauopathy.

**α-polyGR+ staining in Cerebellum**


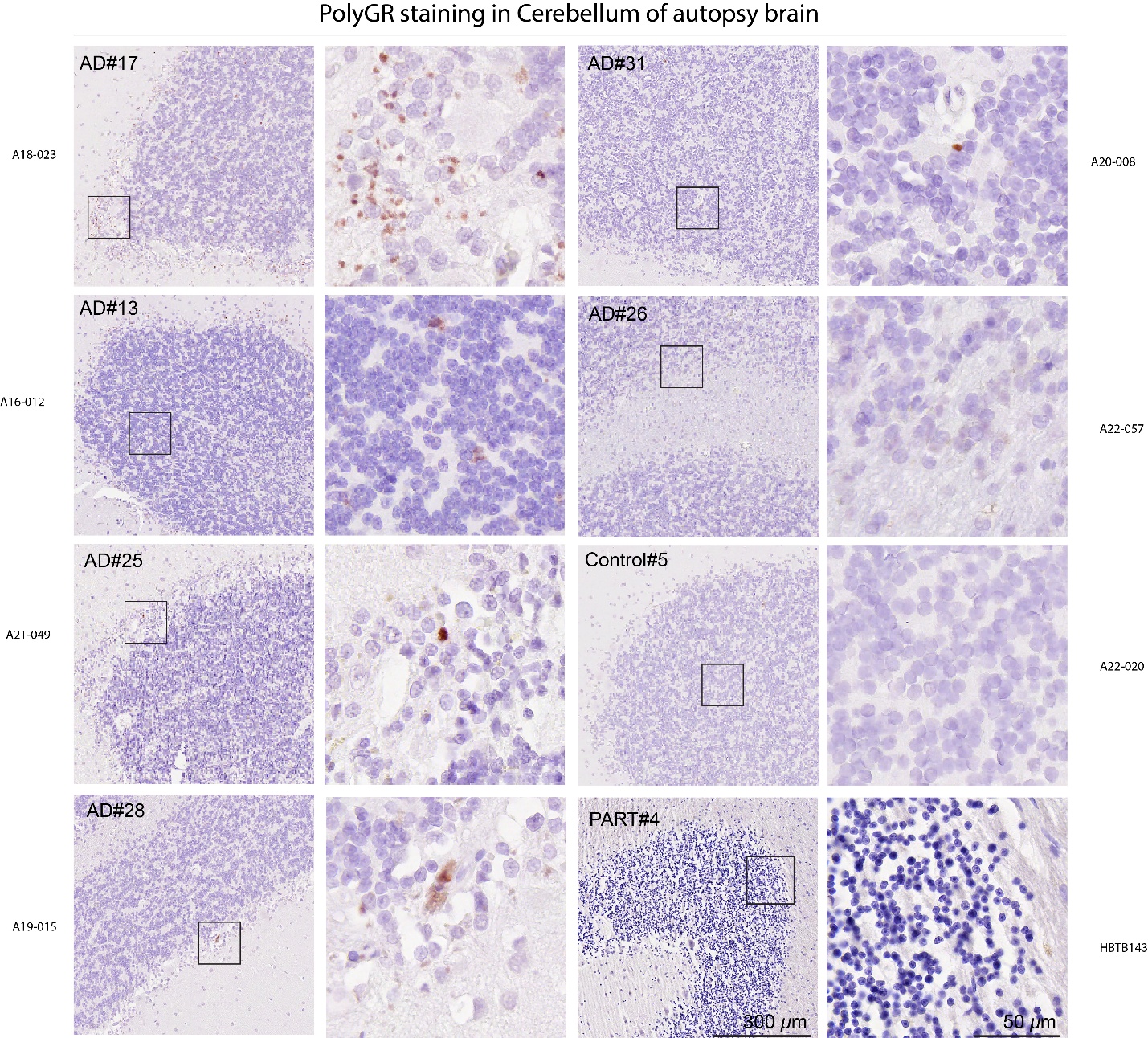


**Figure S5. Widefield and zoomed-in IHC images of polyGR+ staining in the Cerebellum from AD and control autopsy brains.** PolyGR+ staining (red) was detected by using the same IHC staining protocol in hippocampus. The right side of each case is zoomed out from squared area. PART: Primary Age-Related Tauopathy.

**α-polyGR+ staining in Frontal cortex**


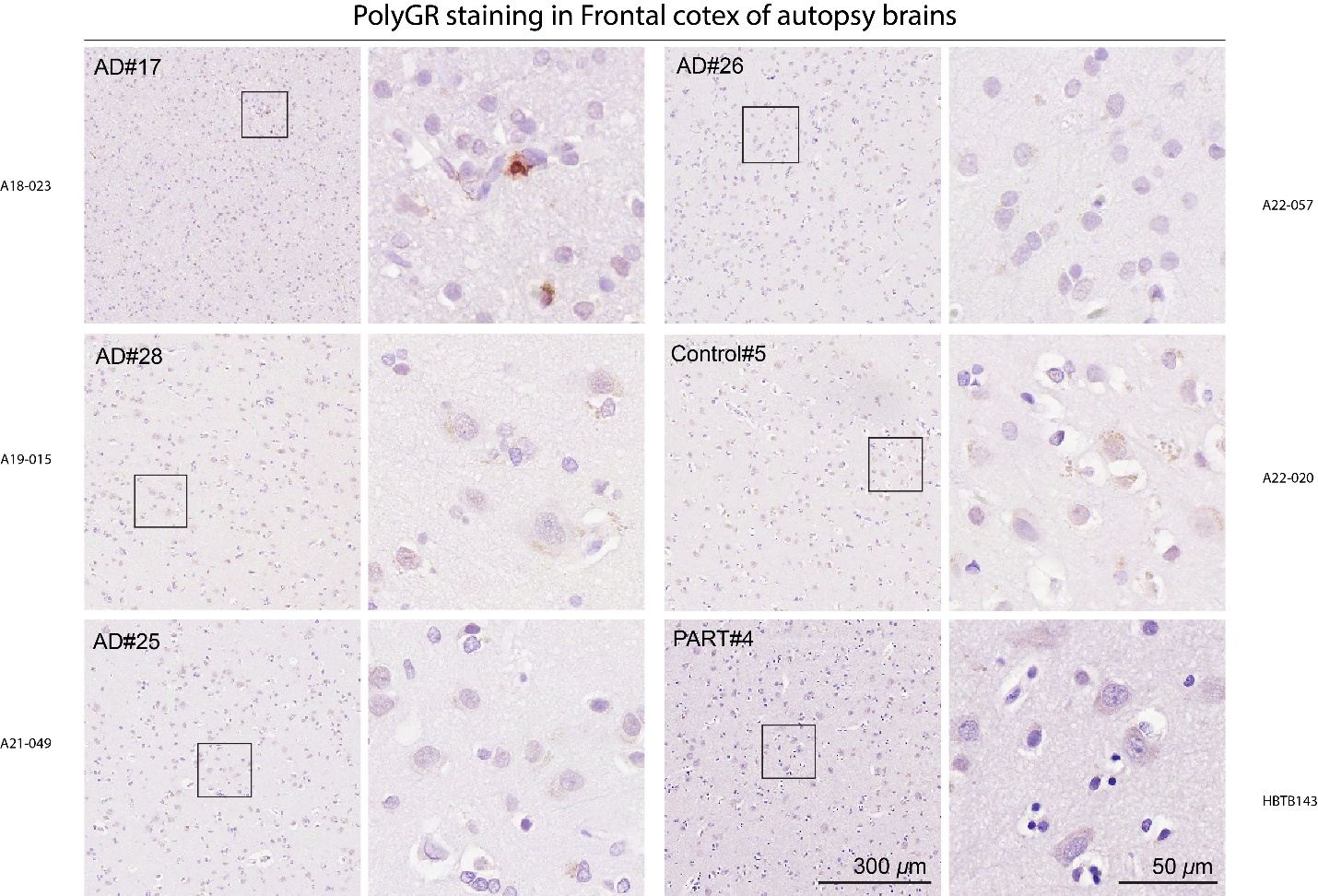


**Figure S6. Widefield and zoomed-in IHC images of polyGR+ staining in the Frontal Cortex from AD and control autopsy brains.** PolyGR+ staining (red) was detected by using the same IHC staining protocol in hippocampus. The right side of each case is zoomed out from squared area. PART: Primary Age-Related Tauopathy.


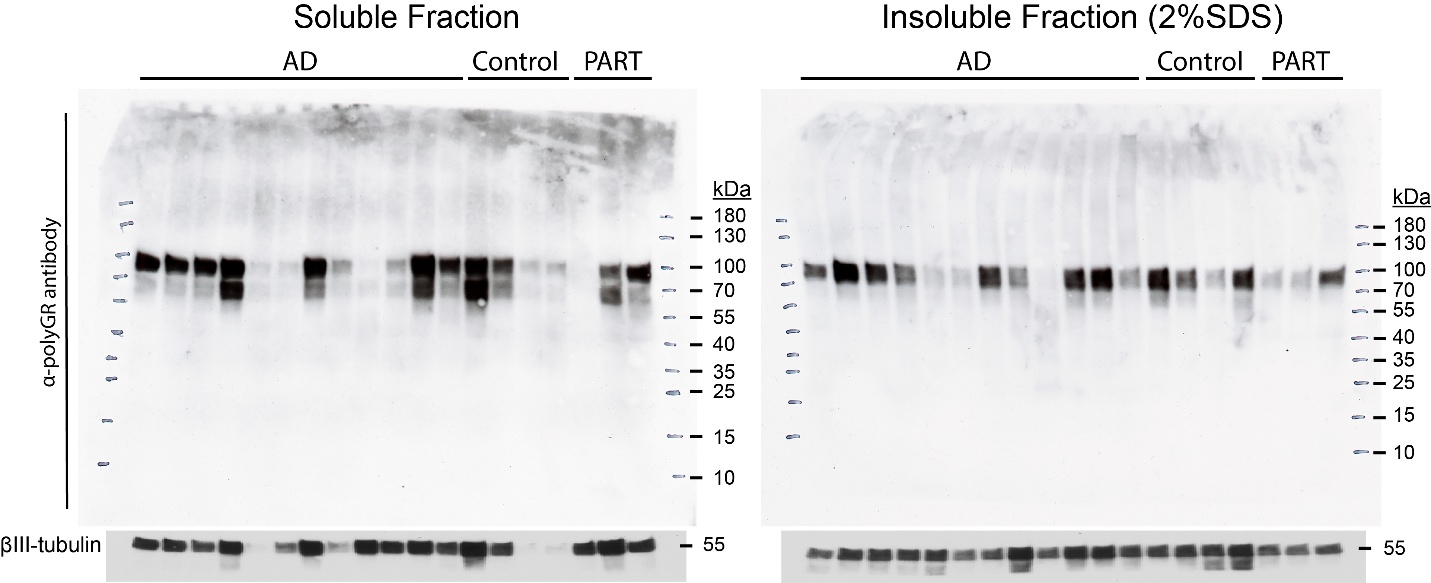


**Figure S7. Image of Western blot using α-polyGR antibody (ref. 120) and denatured proteins from soluble protein fractions extracted from frozen frontal cortex of AD (n=12), Control (n=4) and PART (n=3) cases.** PART: Primary Age-Related Tauopathy.

**
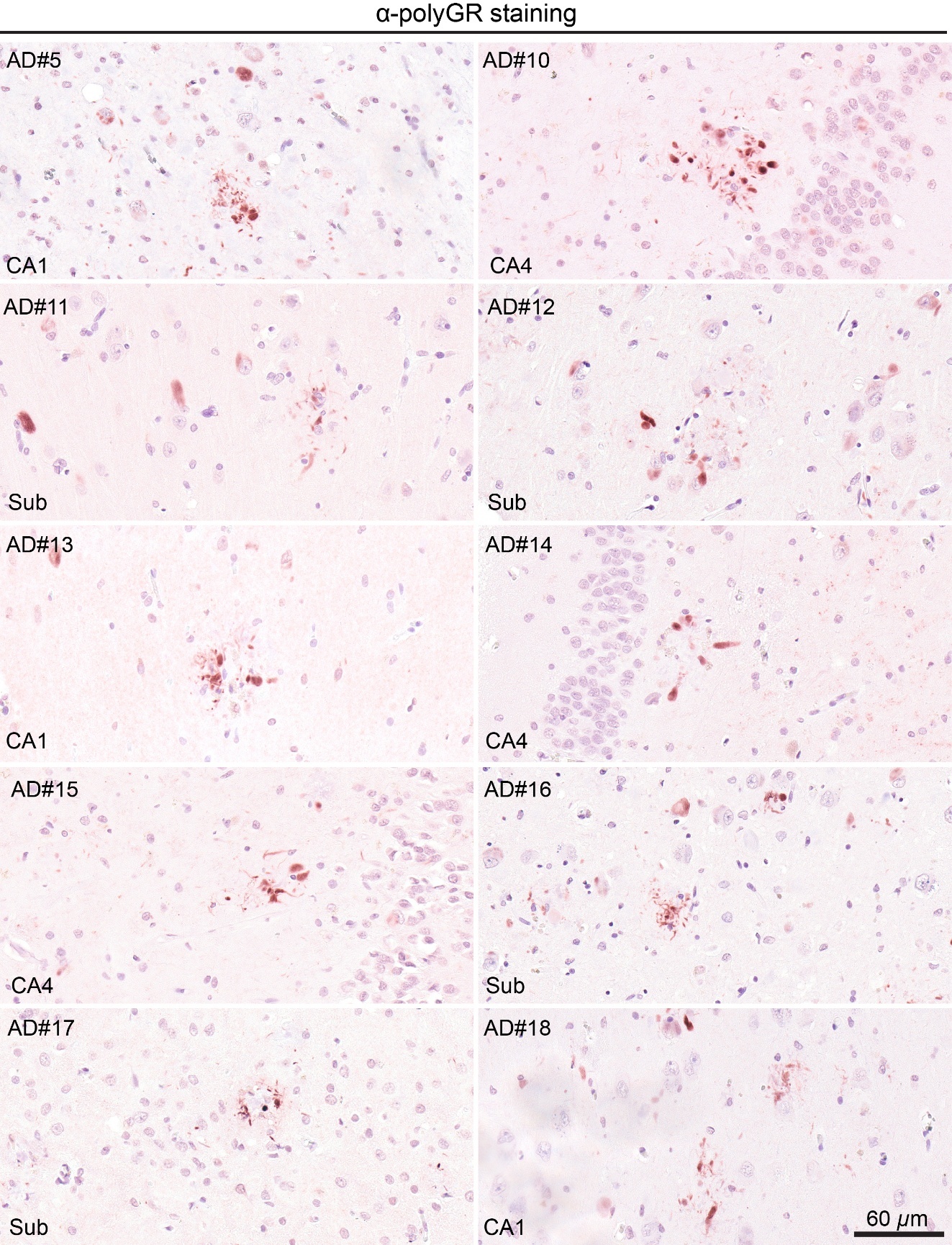
**

**Figure S8. Widefield images of IHC staining showing clustered-punctate polyGR+ staining detected in hippocampal regions from AD autopsy brains.** PolyGR+ staining is red. Sub: Subiculum and CA: Cornu Ammonis

**
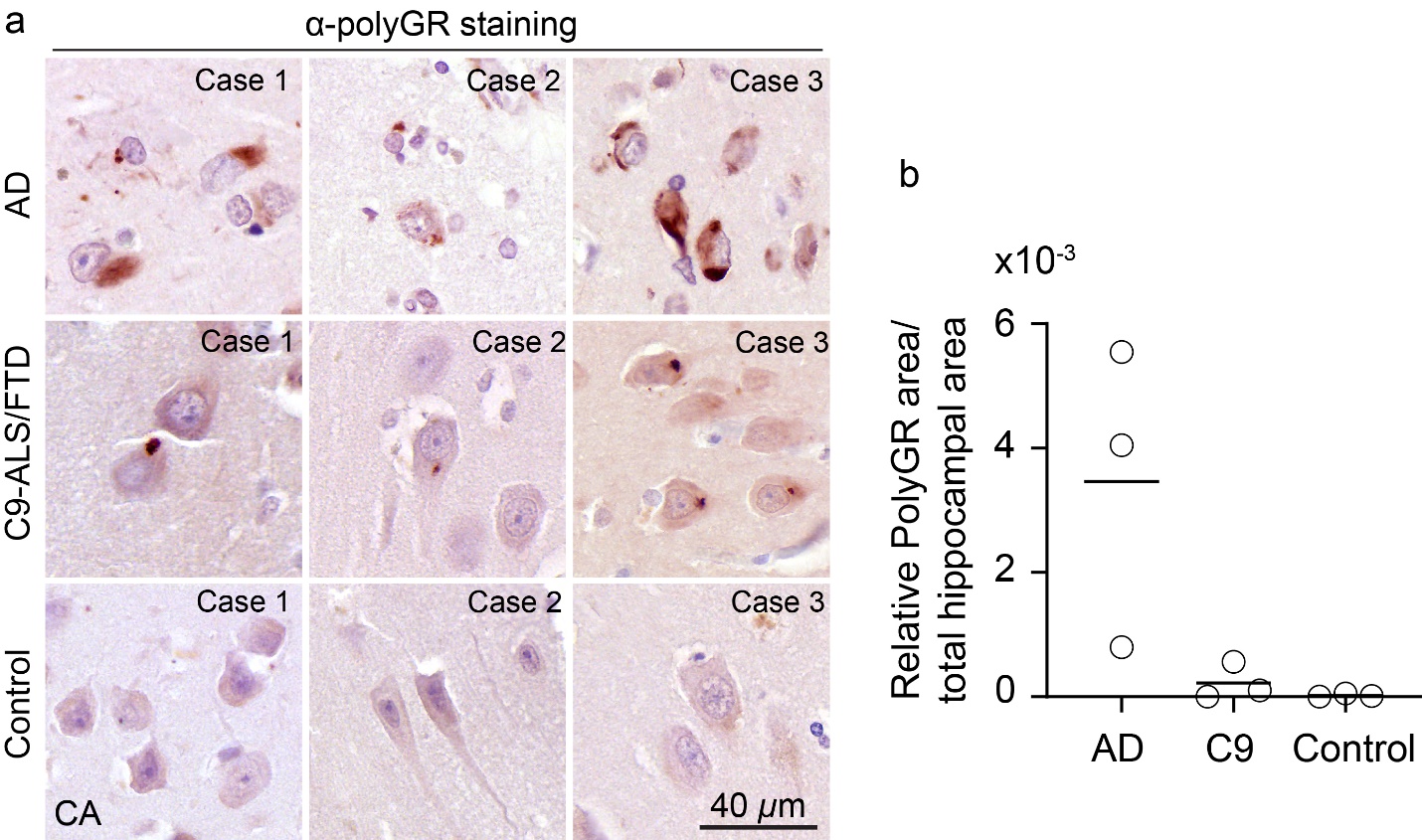
**

**Figure S9. PolyGR+ staining patterns in AD and C9-ALS/FTD autopsy brain tissue.** (a) Examples of immunohistochemical staining showing distinct accumulation patterns of polyGR+ staining (Red) in the Cornu Ammonis (CA) regions from AD and C9-ALS/FTD autopsy brains. PolyGR+ aggregates in AD autopsy brains present large puncta or fibrillary staining in cells with large or small nuclei. PolyGR aggregates in C9-ALS/FTD brains present as star-shape like puncta in hippocampal pyramidal cells, consistent to the polyGR staining described in the previously published studies. No similar polyGR+ staining was detected in the hippocampal regions from control autopsy brain tissue samples. Each image panel represents different AD, C9-ALS/FTD, or control cases. (b) Qualification of polyGR+ aggregates in hippocampus of AD, C9-ALS/FTD, and control autopsy brains. Data represents mean.


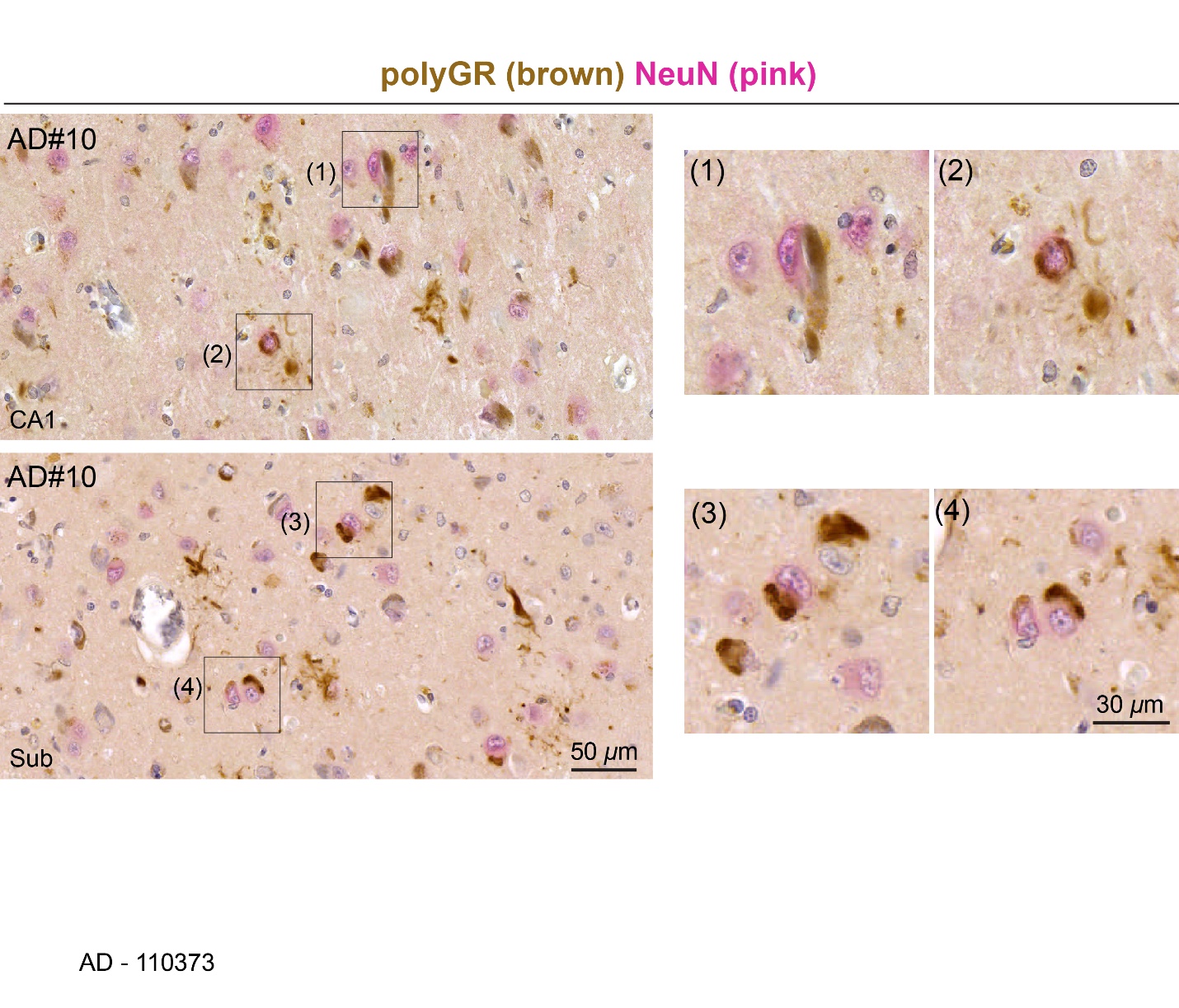


**Figure S10. Co-staining showing polyGR+ aggregates are present in NeuN+ cells in the hippocampus of an AD autopsy brains.** PolyGR+ staining is brown and NeuN staining is pink. CA: Cornu Ammonis

**
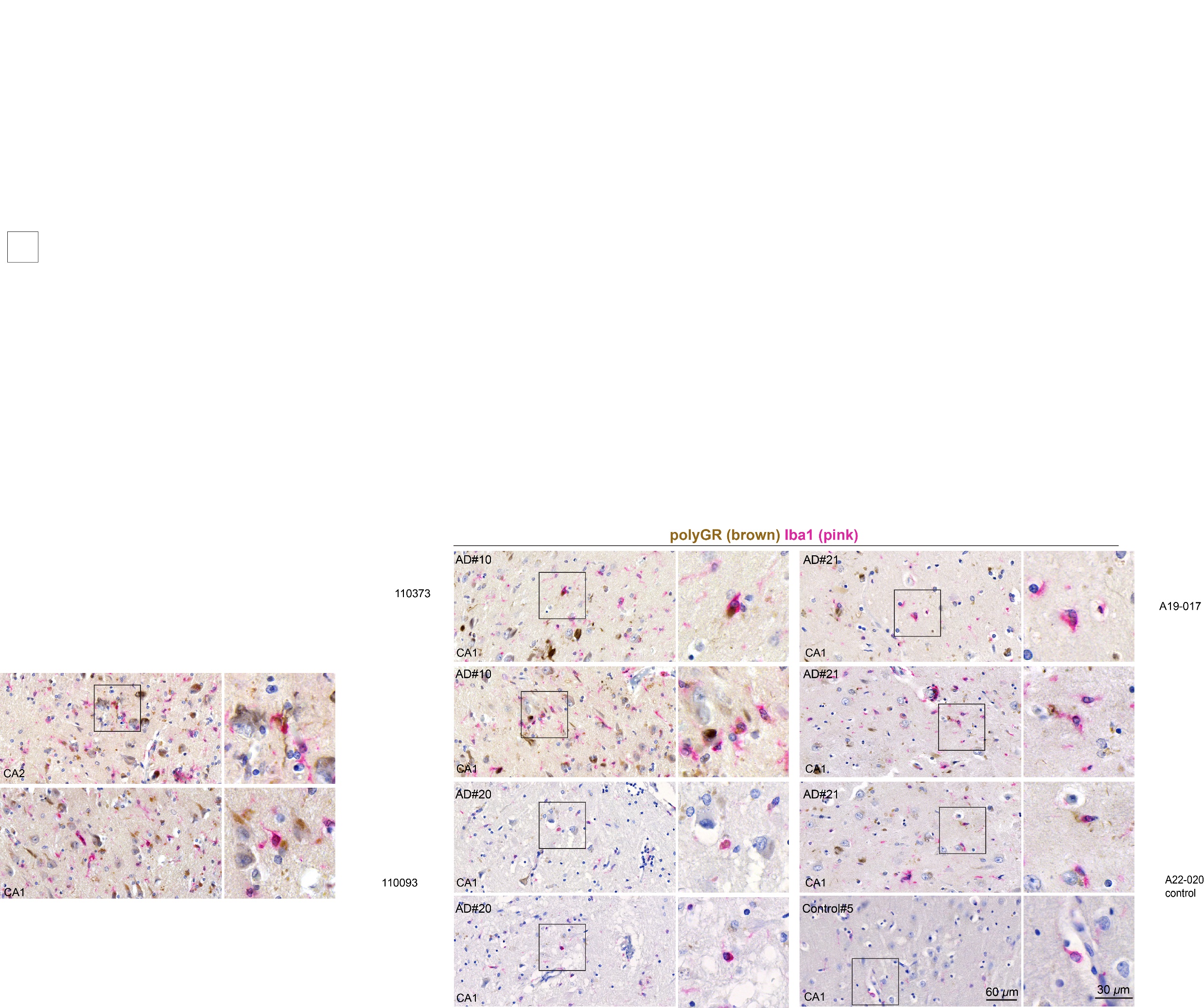
**

**Figure S11. Co-staining showing polyGR+ aggregates are detected Iba1+ cells in the hippocampus of AD autopsy brains.** PolyGR+ staining is brown and Iba1 staining is pink. Sub: Subiculum and CA: Cornu Ammonis

**
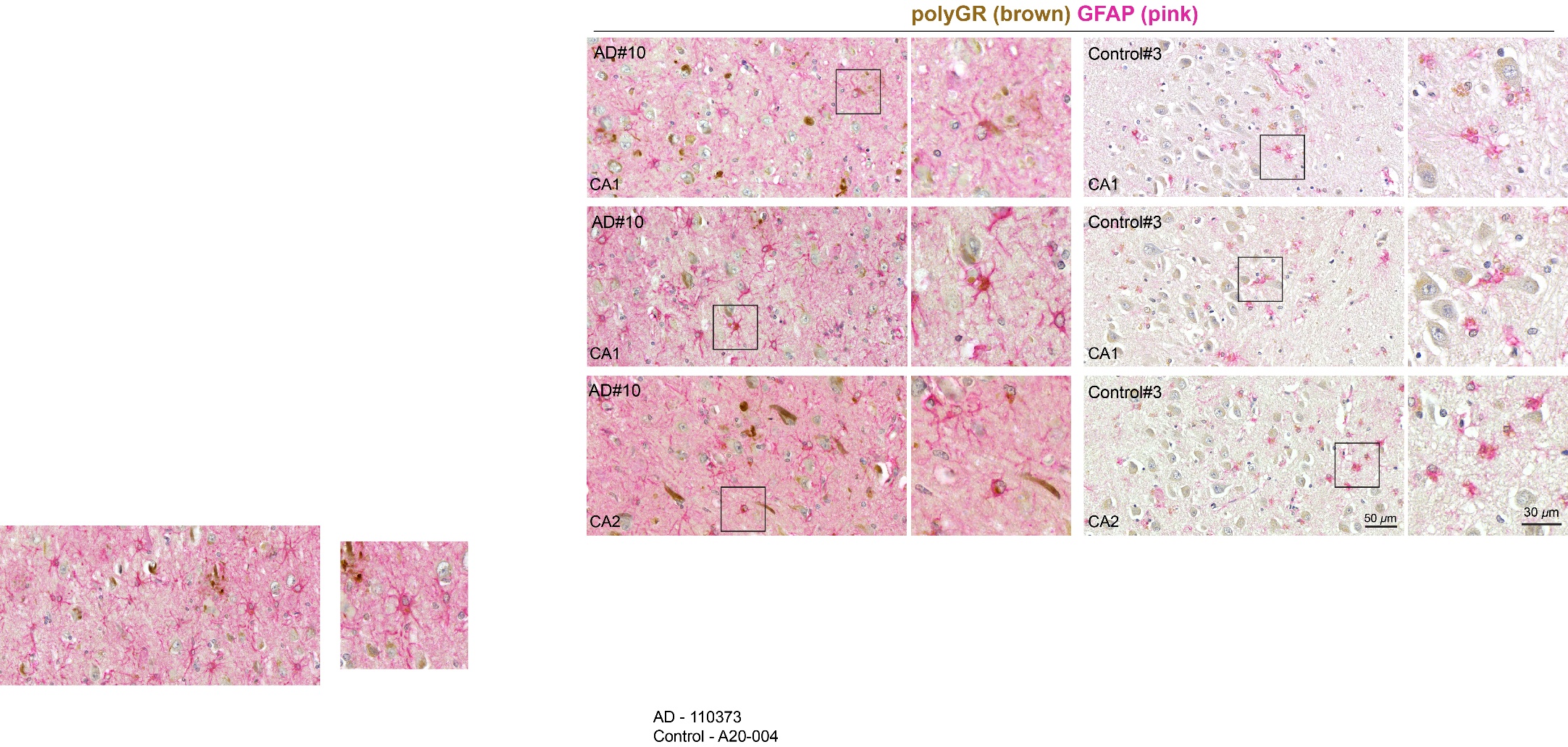
**

**Figure S12. Co-staining showing polyGR+ aggregates are detected GFAP+ cells in the hippocampus of AD autopsy brains**. PolyGR+ staining is brown and GFAP staining is pink. CA: Cornu Ammonis


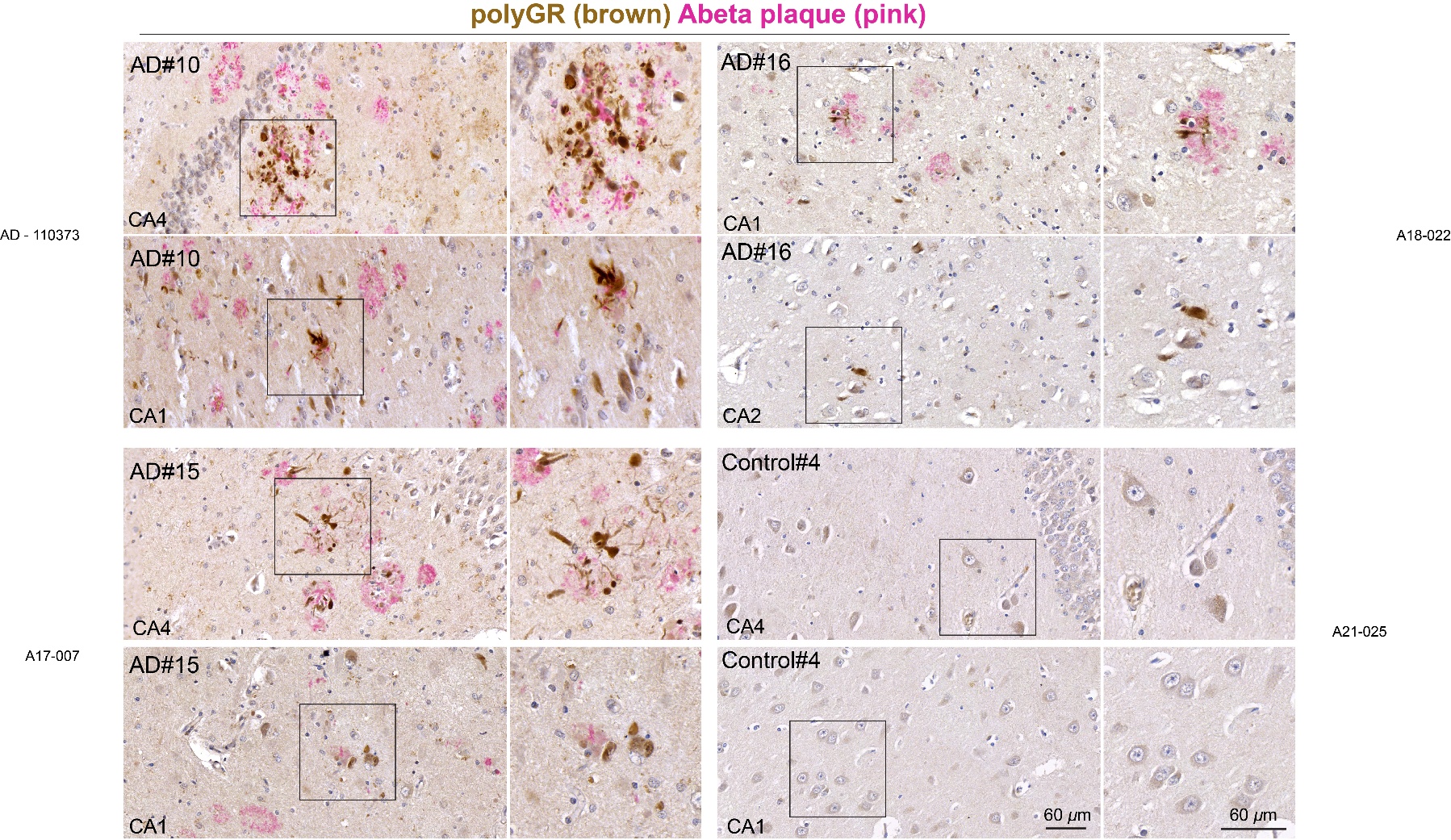


**Figure S13. Co-staining showing the close-proximity region of clustered-punctate polyGR+ aggregates is positive for Aβ plaques**. PolyGR+ staining is brown and abeta plagues staining is pink. CA: Cornu Ammonis


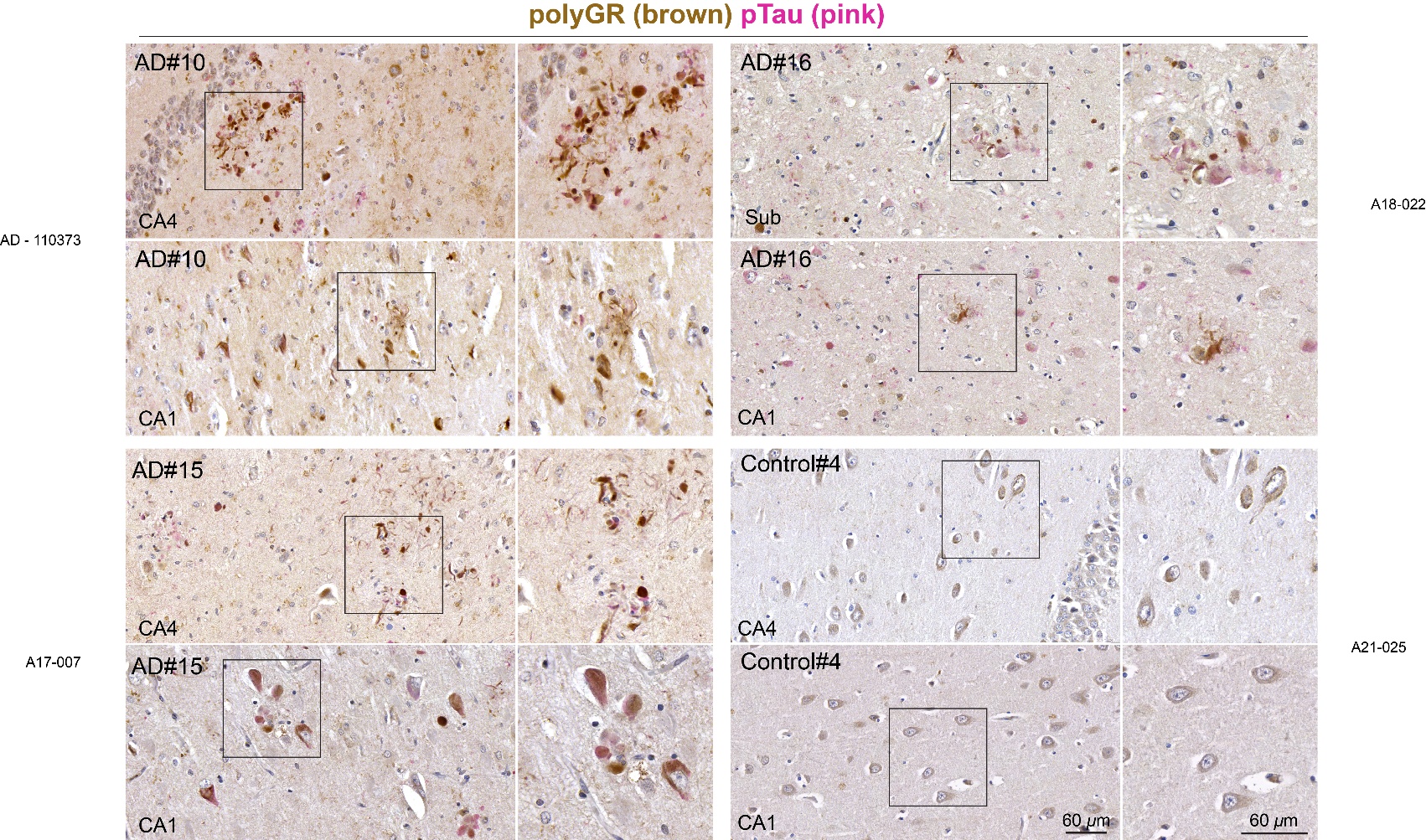


**Figure S14. Co-staining showing clustered-punctate polyGR+ aggregates co-exist with pTau staining.** PolyGR+ staining is brown and pTau staining is pink. CA: Cornu Ammonis

**
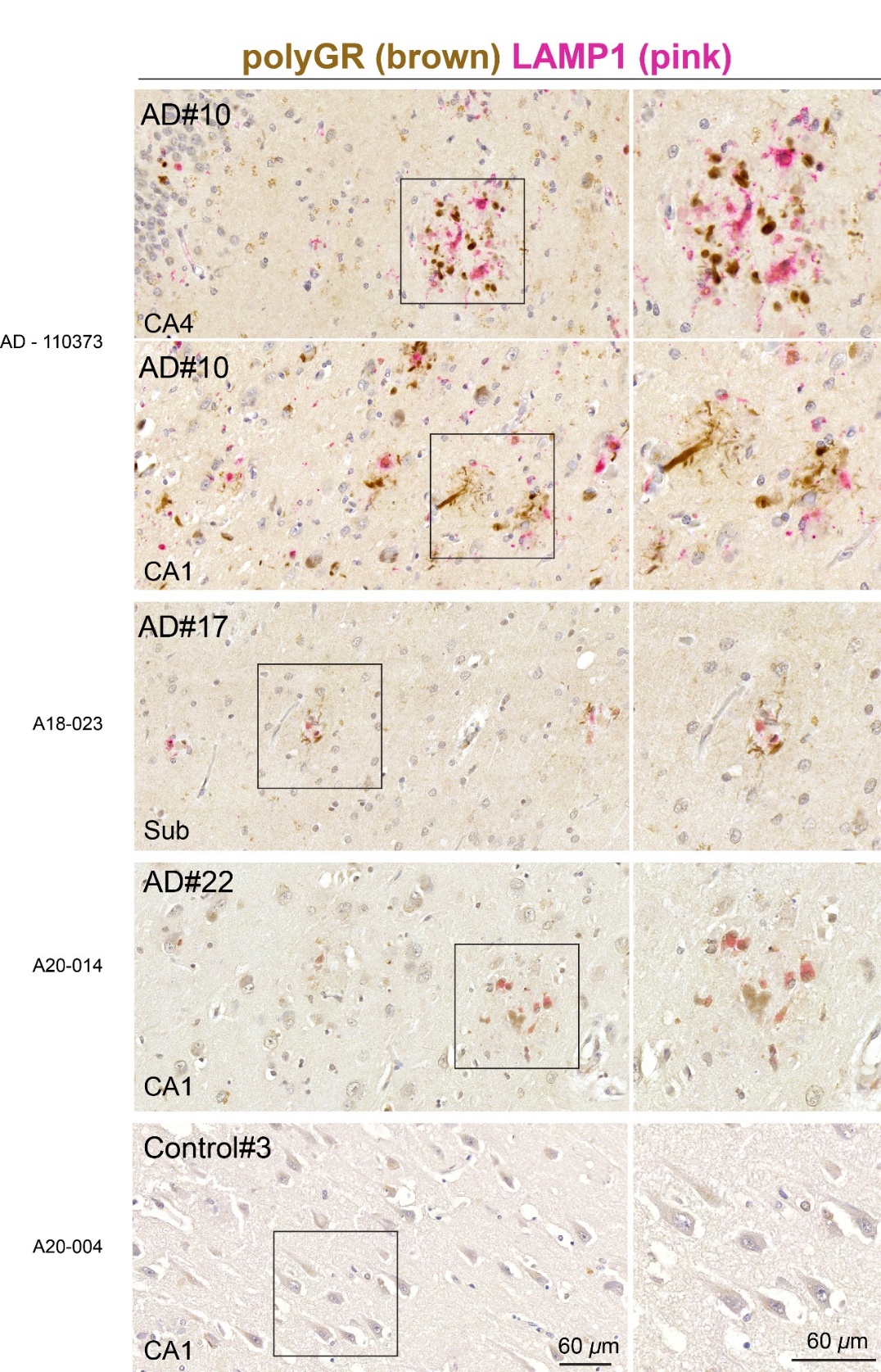
**

**Figure S15. Co-staining showing the close-proximity region of clustered-punctate polyGR+ aggregates is positive for LAMP1 inclusions.** PolyGR+ staining is brown and LAMP1 staining is pink. Sub: Subiculum, CA: Cornu Ammonis.


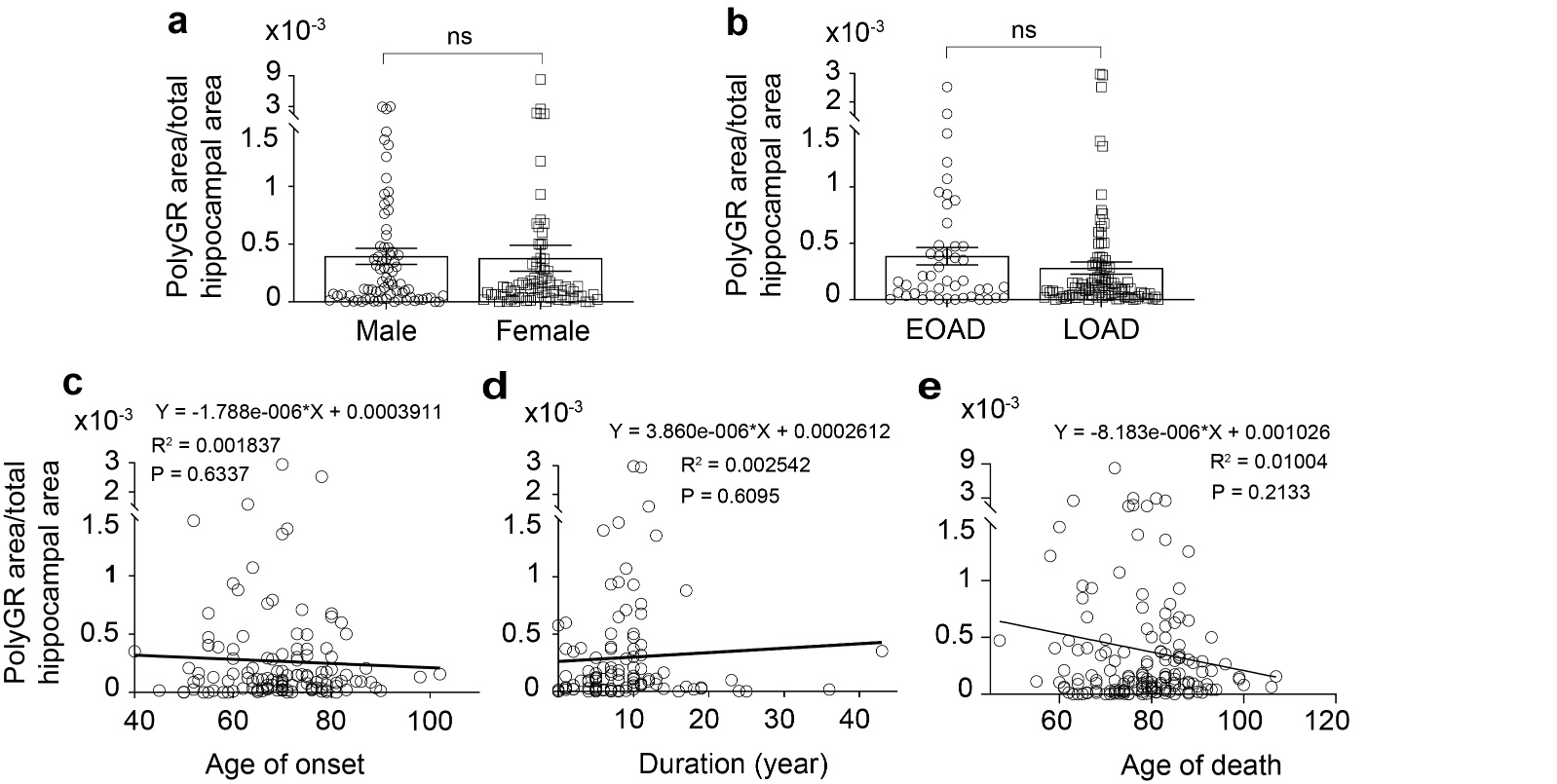


**Figure S16. Levels of polyGR+ aggregates in the hippocampal regions (HC) from AD autopsy brains are not associated with sex, age of onset, disease duration and age of death of AD patients.** (a) No difference was observed for hippocampal polyGR+ levels between male (n = 77) vs female AD cases (n = 79). (b) Graph showing no difference in polyGR+ aggregate levels in the hippocampal regions from autopsy brains of early onset AD (EOAD, n=46) and late onset AD (LOAD, n=97) AD cases. (c, d, e) Simple linear regression shows no correlation of total polyGR+ aggregate levels in the hippocampal regions from AD autopsy brains with age of onset (c) (n = 126), disease duration (d) (n = 105), and age at death (e) (n = 156). Data represents mean ± SEM (a, b) Statistical analyses were performed using unpaired two-tailed Welch’s t-test. ns p>0.05.


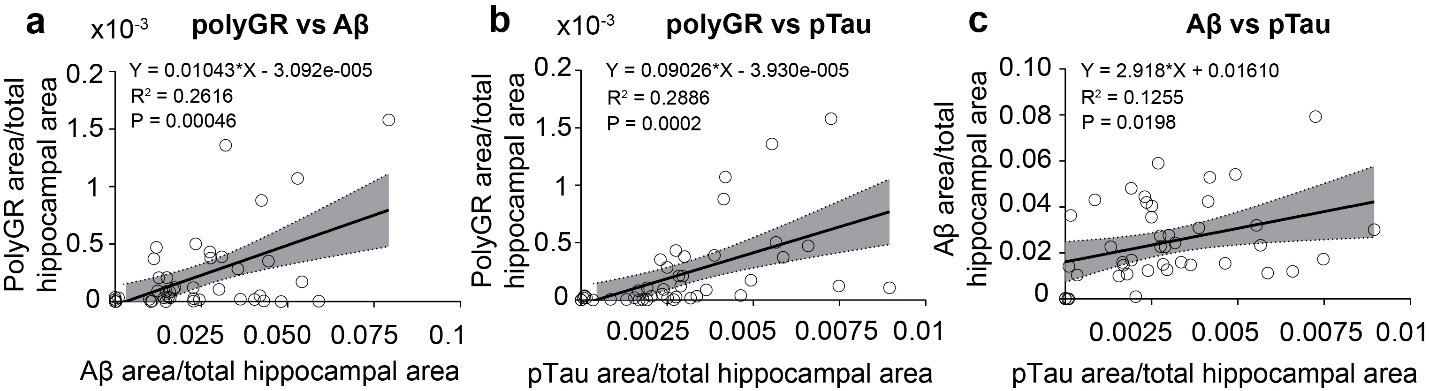


**Figure S17. Total PolyGR+ aggregate levels correlate with levels of Aβ plaques and pTau in the hippocampal regions from AD autopsy brains.** The data were plotted for a sub-cohort of 43 AD cases whose data of three pathological staining are available**.** (a, b) Simple linear regression plots showing a strong association of levels of polyGR+ aggregates with Aβ plaques (a) and pTau (AT8, S202/T205) (b). (c) Simple linear regression plot of levels of Aβ plaques and pTau (AT8, S202/T205).


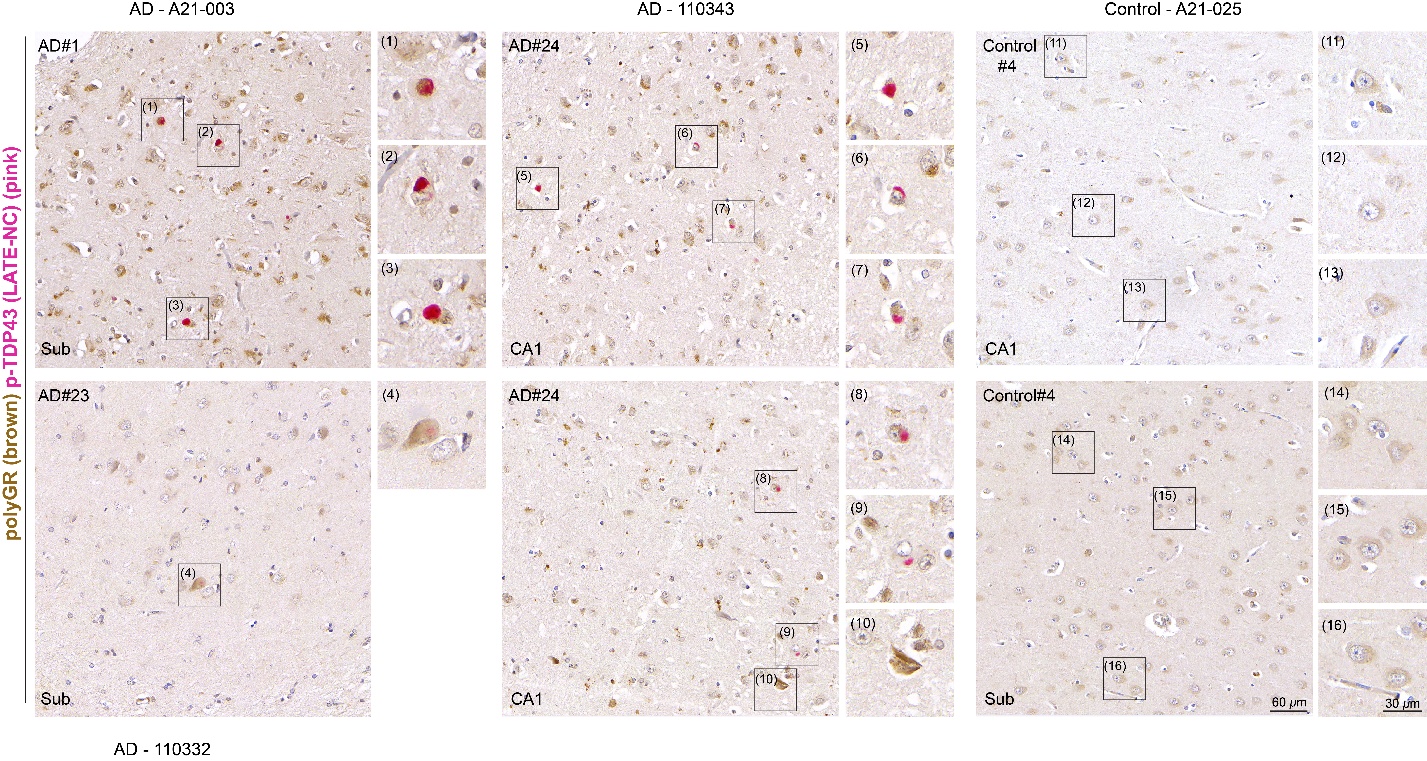


**Figure S18.** **Double IHC staining of polyGR+ aggregates (brown) and p-TDP43 (pink)** **in AD autopsy brains with limbic-predominant age-related TDP-43 encephalopathy neuropathological changes (LATE-NC).** The data shows that polyGR+ aggregates and p-TDP43 inclusions are present in the same sub-regions in the hippocampus of AD autopsy brains and are occasionally detected in the same cells (e.g. fields 1, 2, 3, 4, and 5). Sub: Subiculum, CA: Cornu Ammonis.


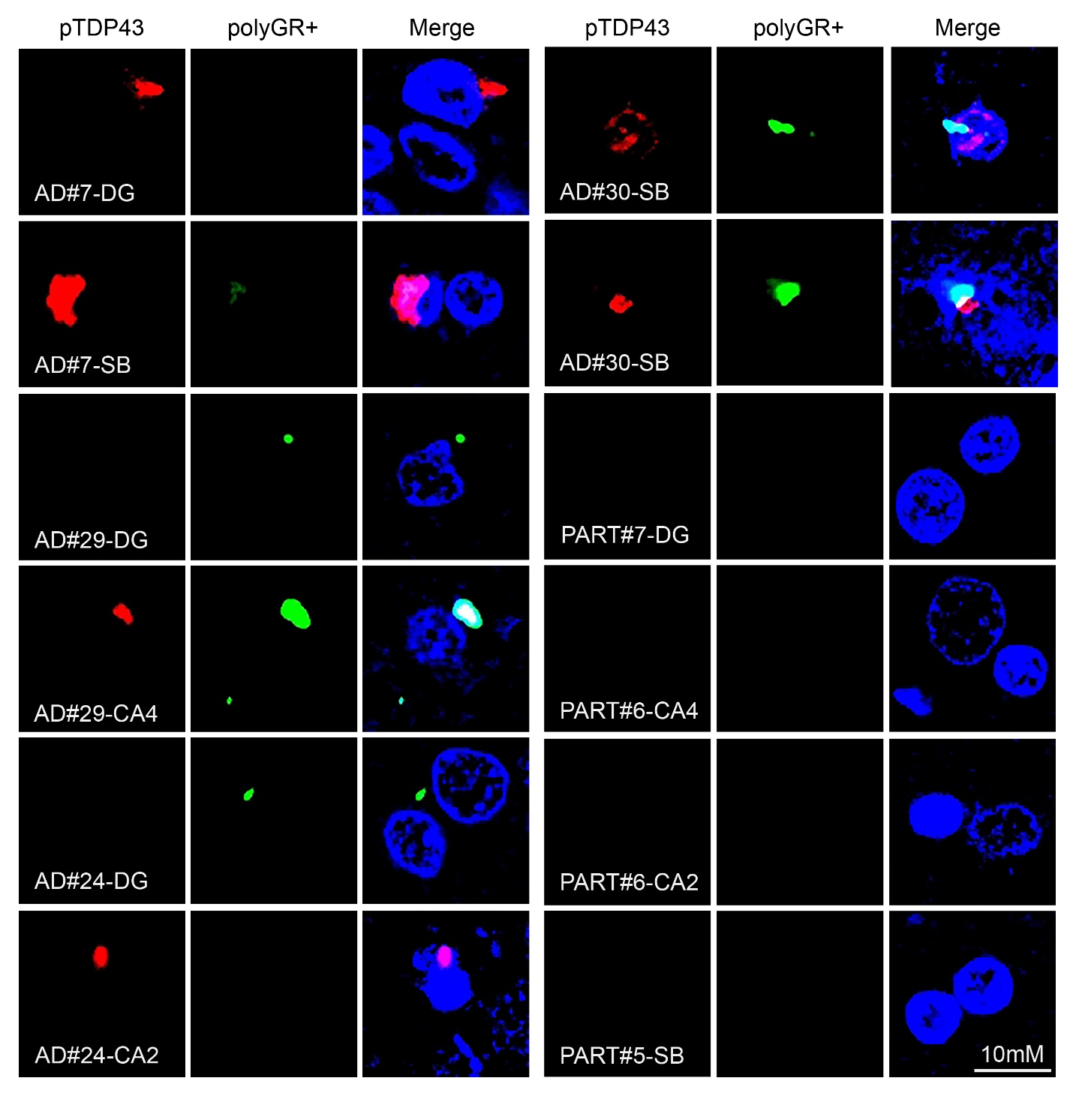
**Figure S19. Widefield IF double staining images of polyGR+ and pTDP-43 staining in the hippocampal regions of AD autopsy brains reported with limbic-predominant age-related TDP-43 encephalopathy neuropathological changes (LATE-NC) and PART autopsy brains.** PolyGR (green) and pTDP-43 (red) staining co-localized, wasfound in the same cell or, in different cells in the hippocampal regions (Sub: Subiculum, CA: Cornu Ammonis, DG: Dentate Gyrus) from AD autopsy brains. Each image panel represents individual AD or control cases. AD cases in this staining were reported with stage 2 of LATE-NC (TDP-43 pathology was present in the hippocampus and entorhinal cortex). PART: Primary Age-Related Tauopathy.


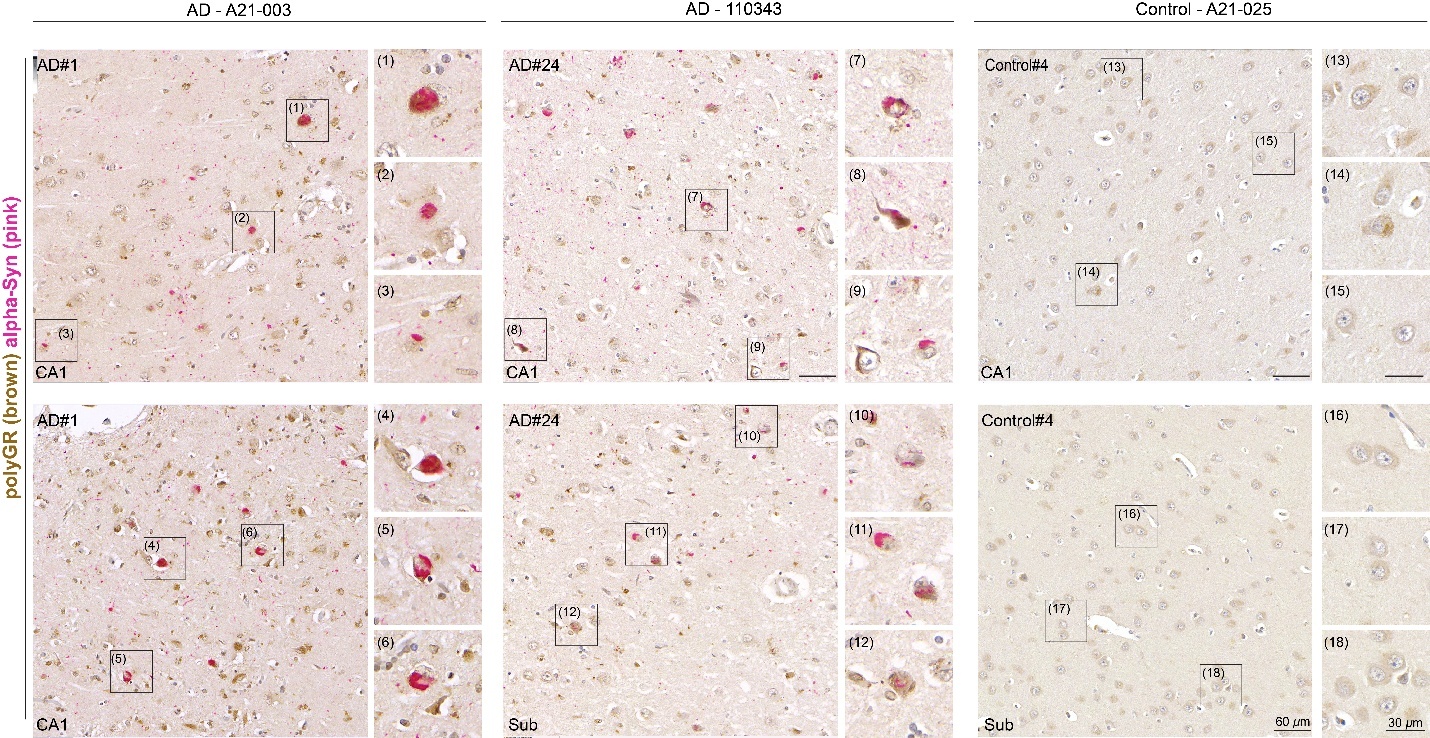


**Figure S20.** **Double IHC staining of polyGR+ aggregates (brown) and α-syn (pink)** **in the AD autopsy brains with Lewy body pathology (LBP).** Our data shows that polyGR+ aggregates and α-syn inclusions are present in the same sub-regions in the hippocampus of AD autopsy brains and are occasionally detected in the same cells (e.g. fields 1, 2, 4, 5, 6, 7, 8, and 11). Sub: Subiculum, CA: Cornu Ammonis.

**EOAD**


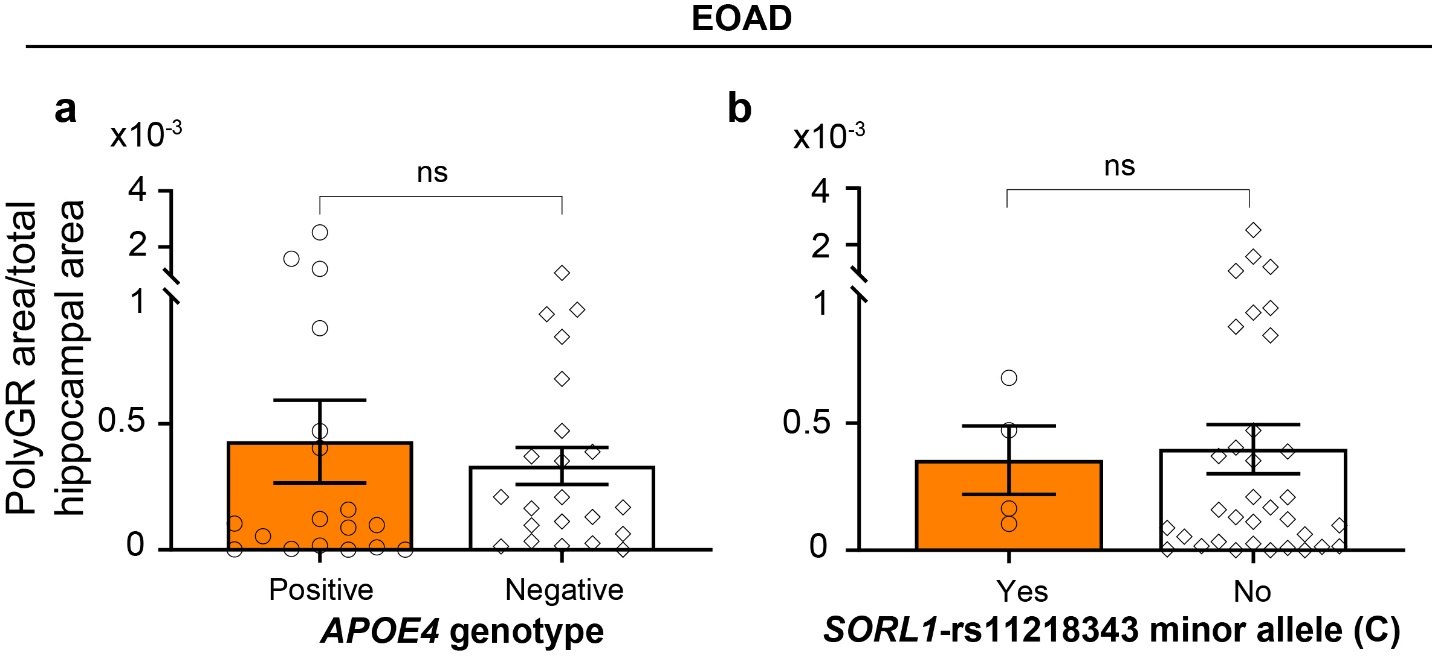


**Figure S21. PolyGR+ aggregate levels in the hippocampus of the autopsy brains from early onset AD cases (EOAD) with or without *APOE4* allele(s).** positive n = 18, negative n = 22. Statistical analysis was performed using unpaired two-tailed Welch’s t-test. Data represents mean ± SEM. ns p>0.05


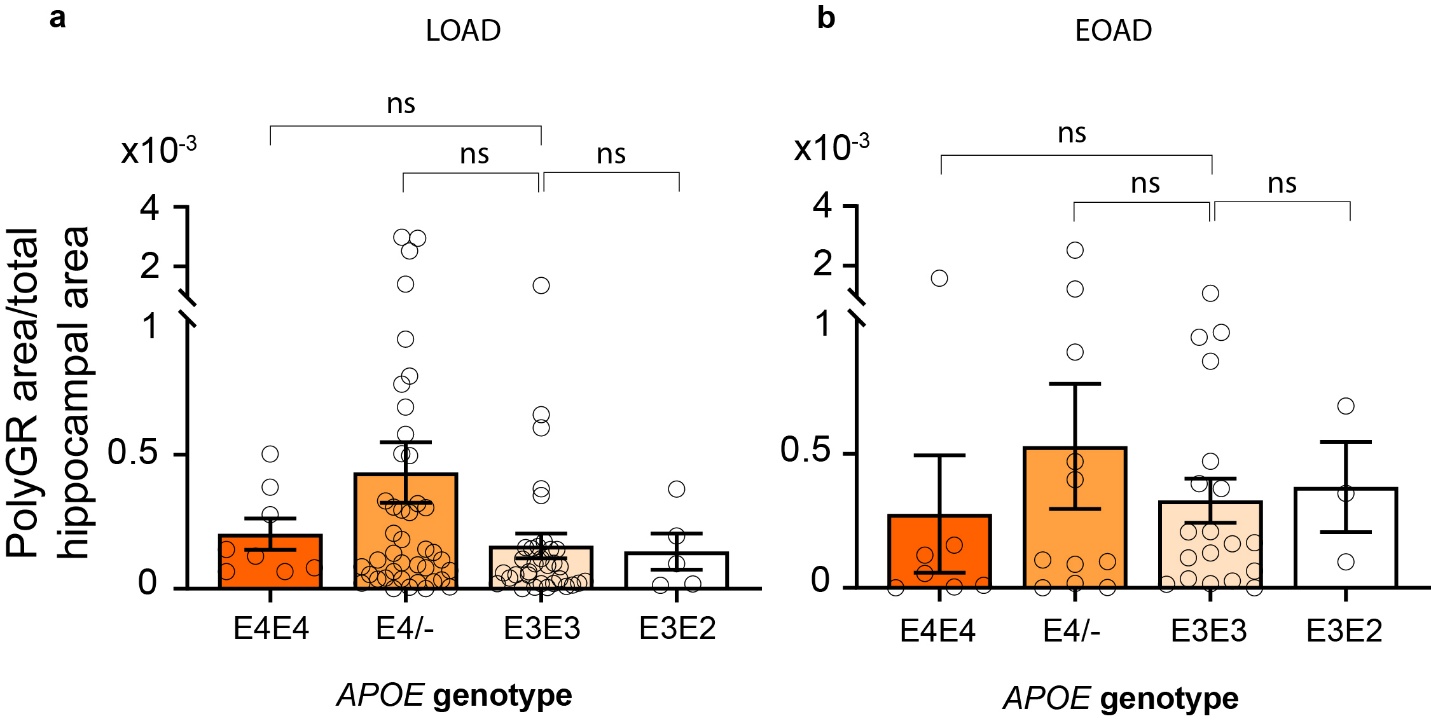


**Figure S22. PolyGR+ aggregates levels in the hippocampus of autopsy brains did not differ in *APOE* genotype groups.** (a) *APOE* genotype in LOAD: E4/E4 (n=8), E4/- (n=42), E3/E3 (n=33), E3/E2 (n=5). (b) *APOE* genotype in EOAD: E4/E4 (n=7), E4/- (n=11), E3/E3 (n=19), E3/E2 (n=3). Data represent mean ± SEM. Statistical analyses were performed using one-way ANOVA with Brown-Forsythe test**.** ns: p>0.05.


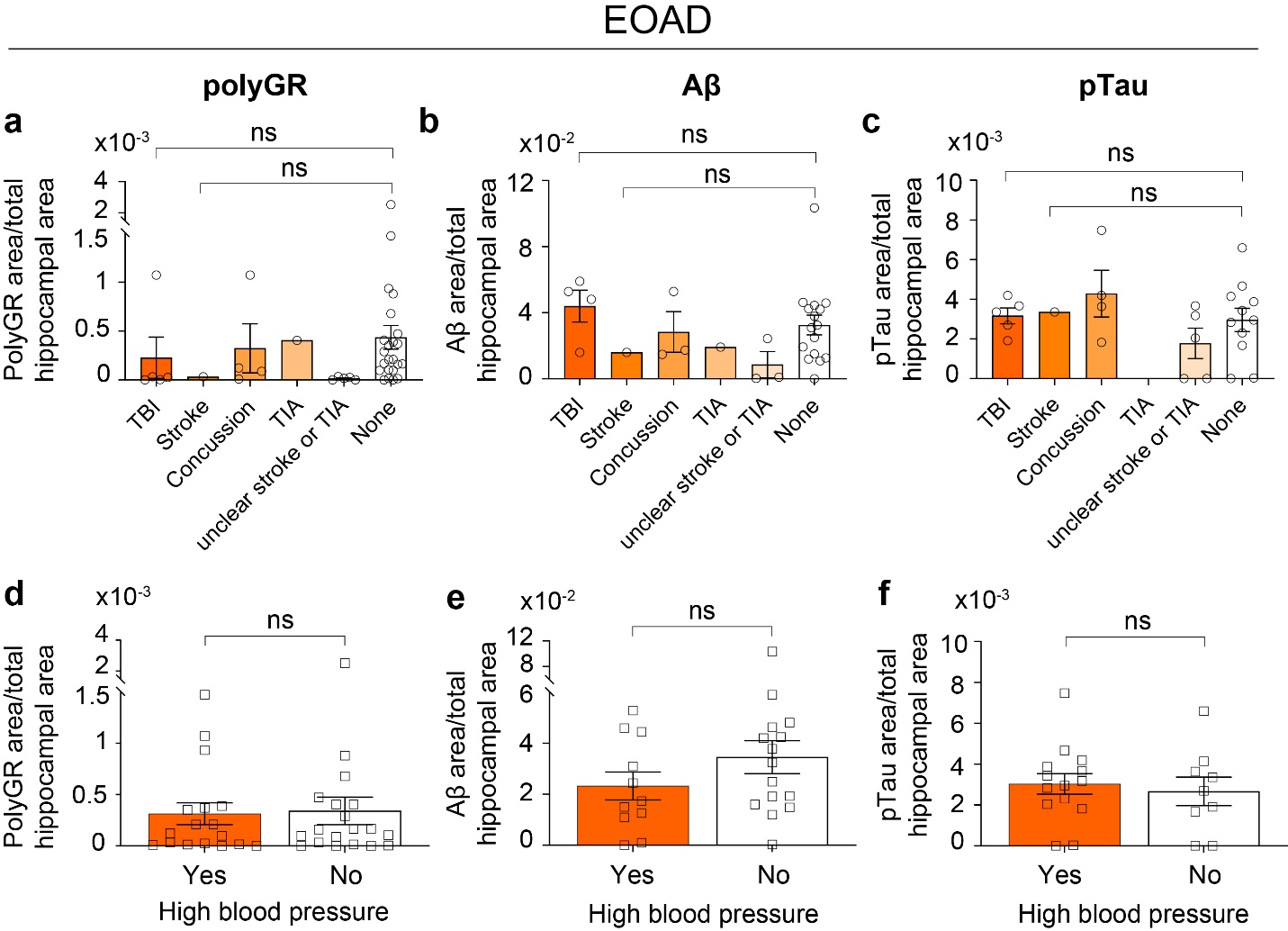


**Figure S23. PolyGR+ aggregate levels in early onset AD (EOAD) cases with or without TBI, Stroke, TIA or high blood pressure.** (a, b, c) Plots showing levels of polyGR+ aggregates (a) (n = 36), Aβ plaques (b) (n = 26), and pTau (c) (n = 23) EOAD cases with or without a history of brain injuries (TBI, concussion, stroke, and TIA). (d, e, f) Plots showing levels of polyGR+ aggregates (d) (n = 36), Aβ plaques (e) (n = 26), and pTau (f) (n = 23) in EOAD cases with or without a history of high blood pressure. Data represents mean ± SEM. Statistical analyses were performed using ordinary one-way ANOVA with Sidak test. Statistical analyses of high blood pressure were performed using unpaired two-tailed Welch’s t-test. ns p>0.05.

**
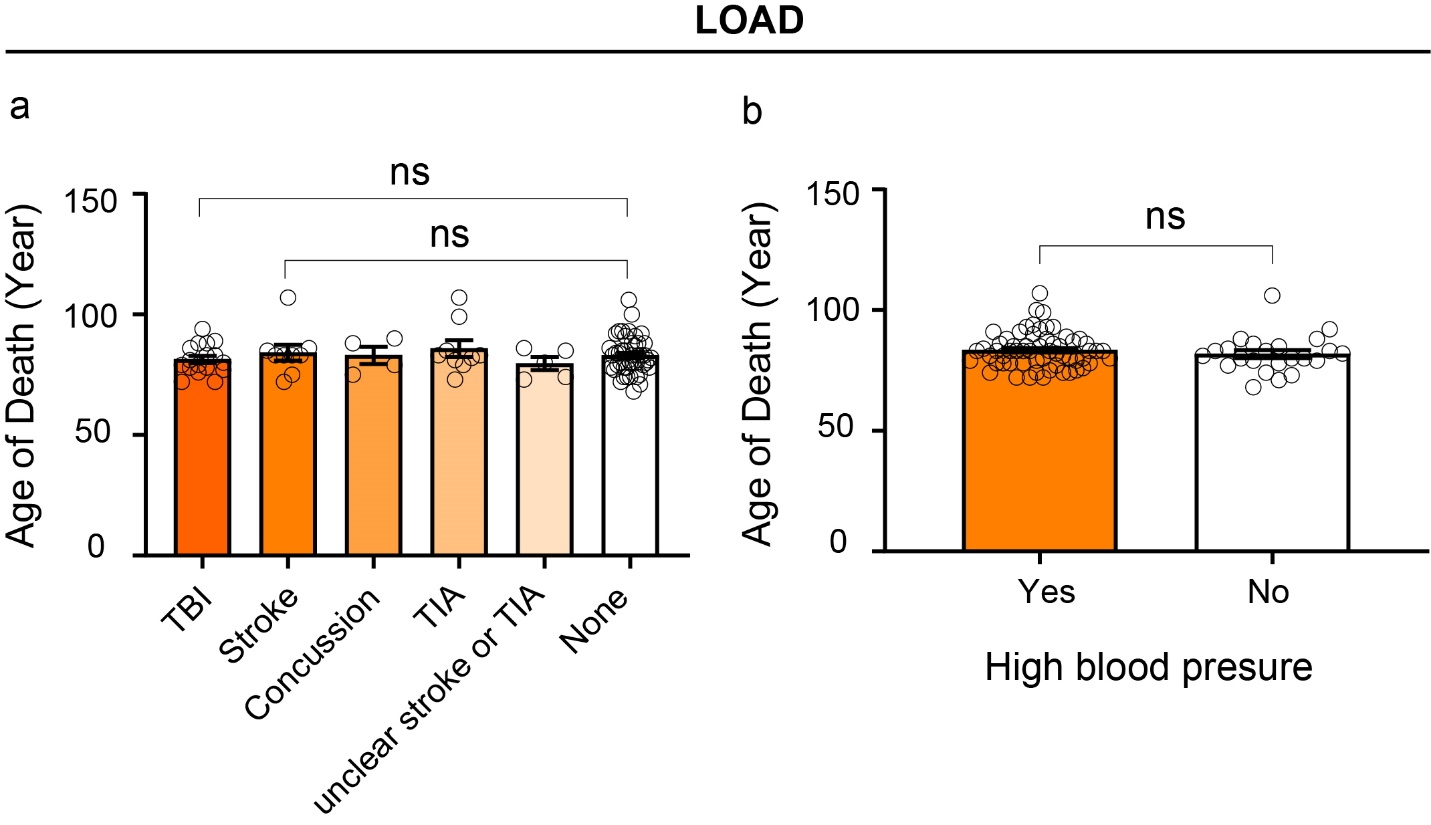
**

**Figure S24. Age of death in late onset AD (LOAD) cases with or without TBI, Stroke, concussion, TIA, or high blood pressure.** Plots showing age of death of LOAD cases with or without brain injuries or high blood pressure (shown in Figure 5). Statistical analyses were performed using unpaired two-tailed Welch’s t-test. Data represents mean ± SEM. ns p>0.05.


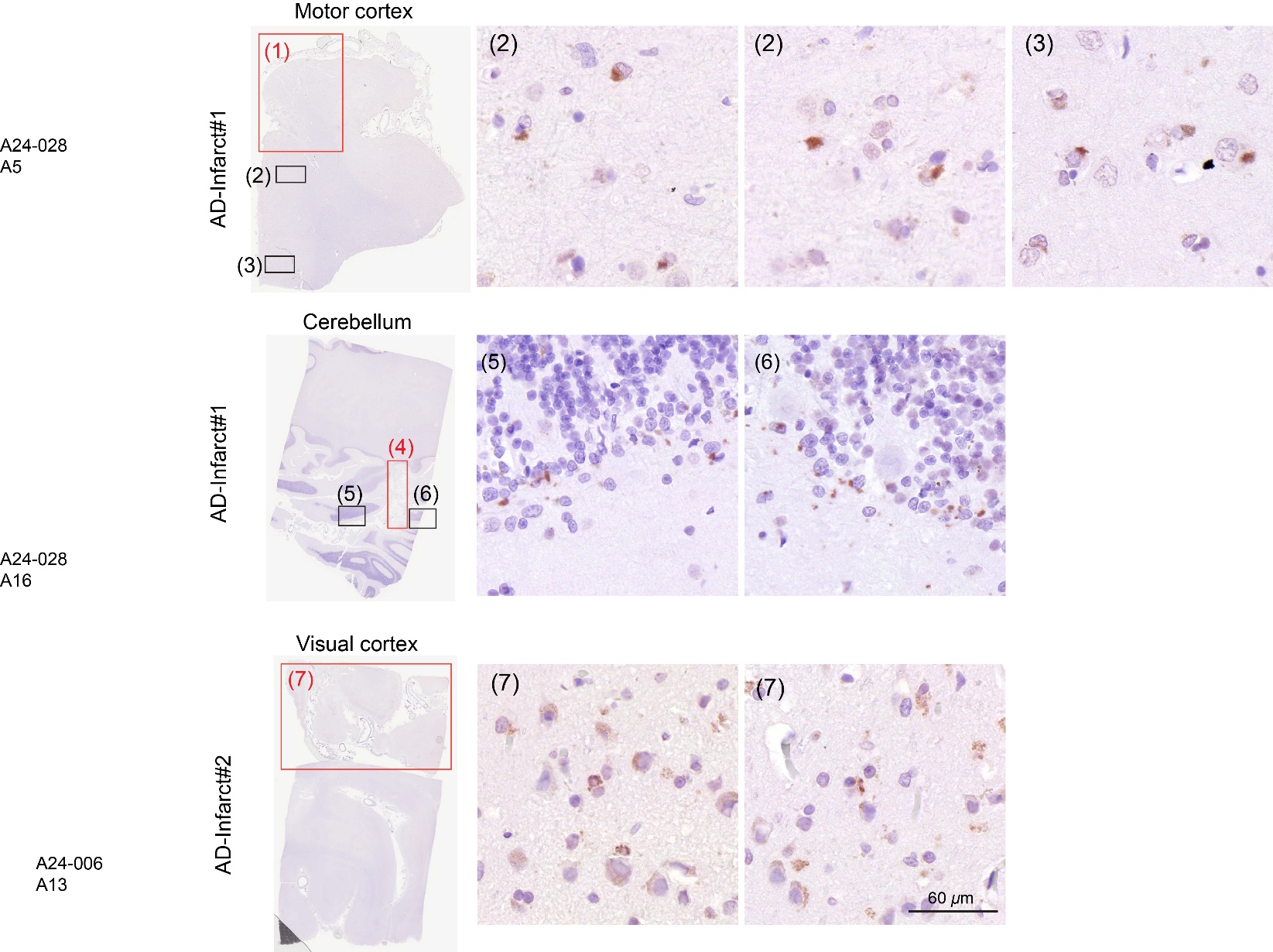


**Figure S25. PolyGR+ aggregate staining in AD brain sections with characterized infarcts.** PolyGR+ staining (red) was detected by using the same IHC staining protocol in hippocampus. Red box: areas with infarct.


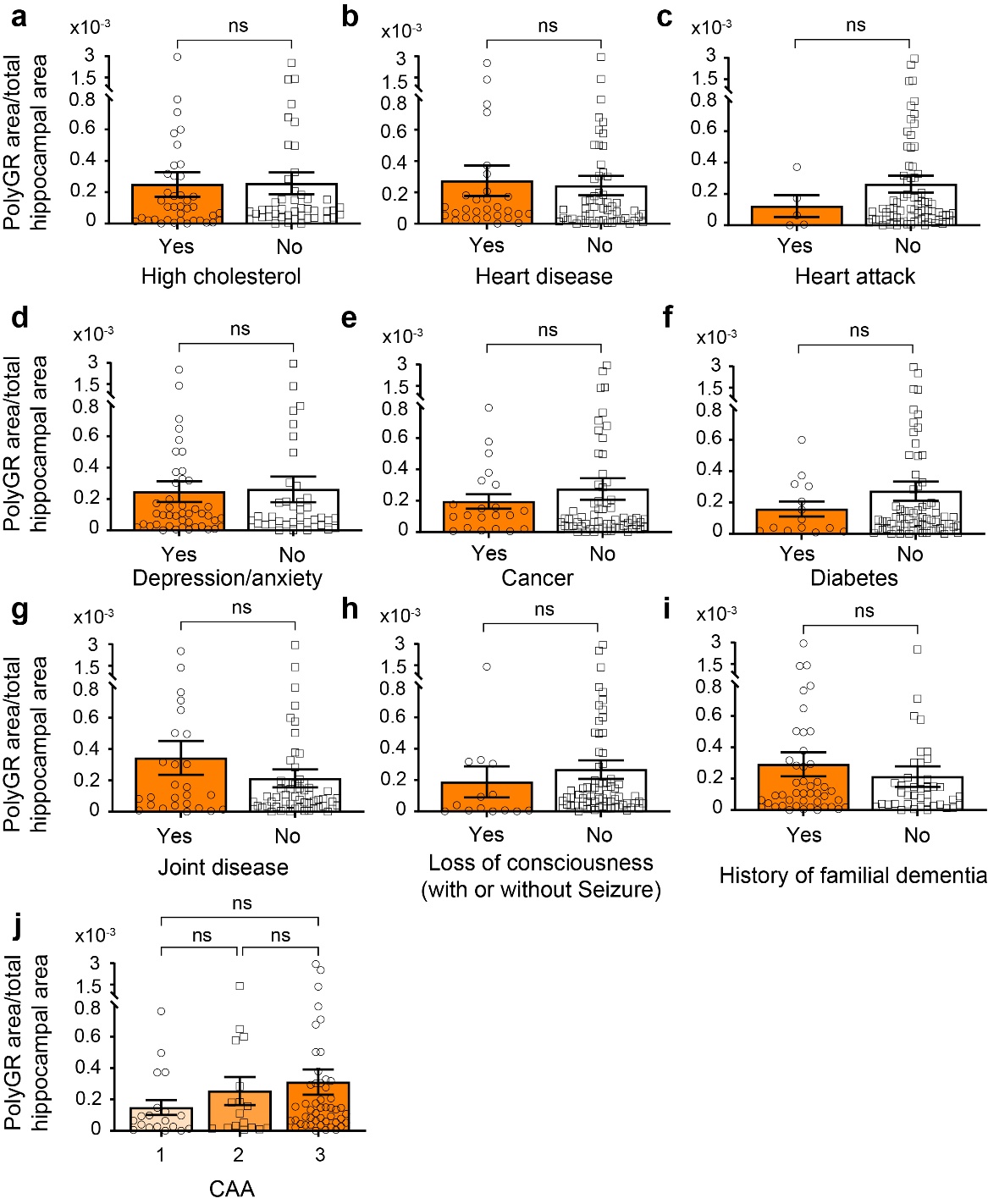


**Figure S26. PolyGR+ aggregate levels in the hippocampus of LOAD cases with additional comorbidities.** Levels of polyGR+ aggregates in the hippocampal regions from LOAD autopsy brains were compared between AD cases with and without (a) high cholesterol (n=84), (b) heart disease (n=84), (c) heart attacks (n=85), (d) depression/anxiety (n=84), (e) cancer (n=83), (f) diabetes (n=84), (g) joint disease (n=84), (h) loss of consciousness with or without seizure (n=84), (i) family history with dementia (n=85), and (j) cerebral amyloid angiopathy (CAA) (n=81 with three categories: 1-Focal, 2-Widespread mild to moderate, 3-Widespread moderate to severe. (j). Data represent mean ± SEM. Statistical analyses were performed using unpaired two-tailed t-test (a, b, c, d, e, f, g, i, j, k) or One-way ANOVA with Brown-Forsythe test (h). ns: p>0.05.

**
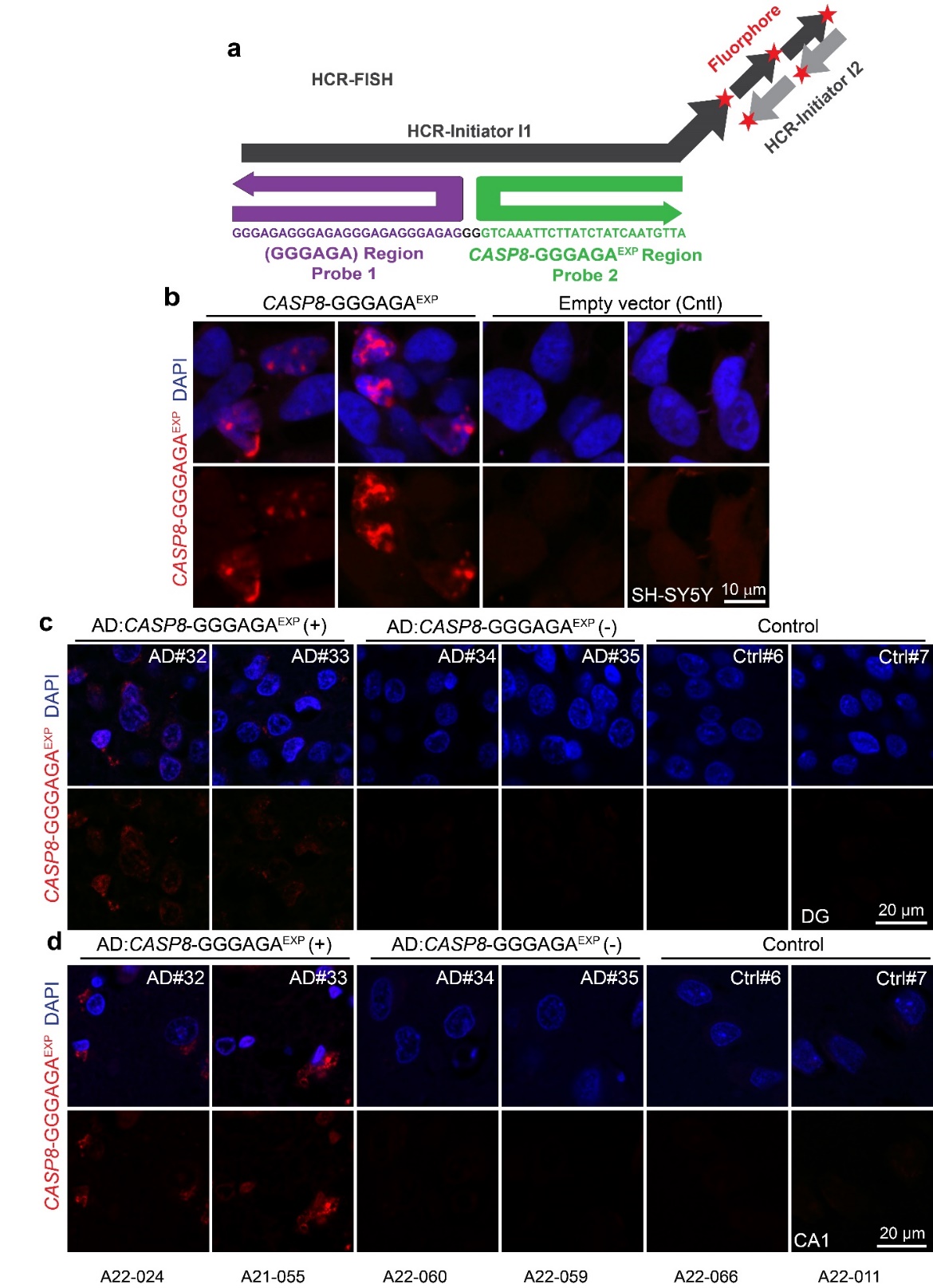
**

**Figure S27: Hybridization Chain Reaction Fluorescence In Situ Hybridization (HCR-FISH) detects** **rGGGAGA RNA transcripts expressed from the *CASP8* repeat expansion in AD autopsy brains that carry this repeat expansion.** (a) GGGAGA-Probe1 and *CASP8*-GGGAGA^EXP^-Probe2 bind to the GGGAGA repeat region and the *CASP8* unique flanking sequence downstream of the repeat, respectively. Successful hybridization of both Probe1 and Probe2 allows HCR initiator taged with a fluorophore (I1) to bind to the nucleic acid complex. Next, HCR-I1 probe binds to HCR-I2 taged with a fluorophore making a self-assembly of many fluorophore-labeled hairpin DNA structures into large fluorescent polymers, amplyfying the signal. (b) Representative images of HCR-FISH staining of rGGGAGA repeat transcripts in SH-SY5Y cells transfected with *CASP8*-GGGAGA^EXP^ plasmids (p-AD-R1 or p-C-Var plasmids shown in Fig. 6A in Nguyen et al. [58].(c, d) Representative images of HCR-FISH staining of *CASP8* rGGGAGA repeat transcripts in the DG (c) and CA regions (d) of AD cases positive and negative for the *CASP8*-GGGAGA^EXP^ and control cases. DG: Dentate Gyrus CA: Cornu Ammonis.

**
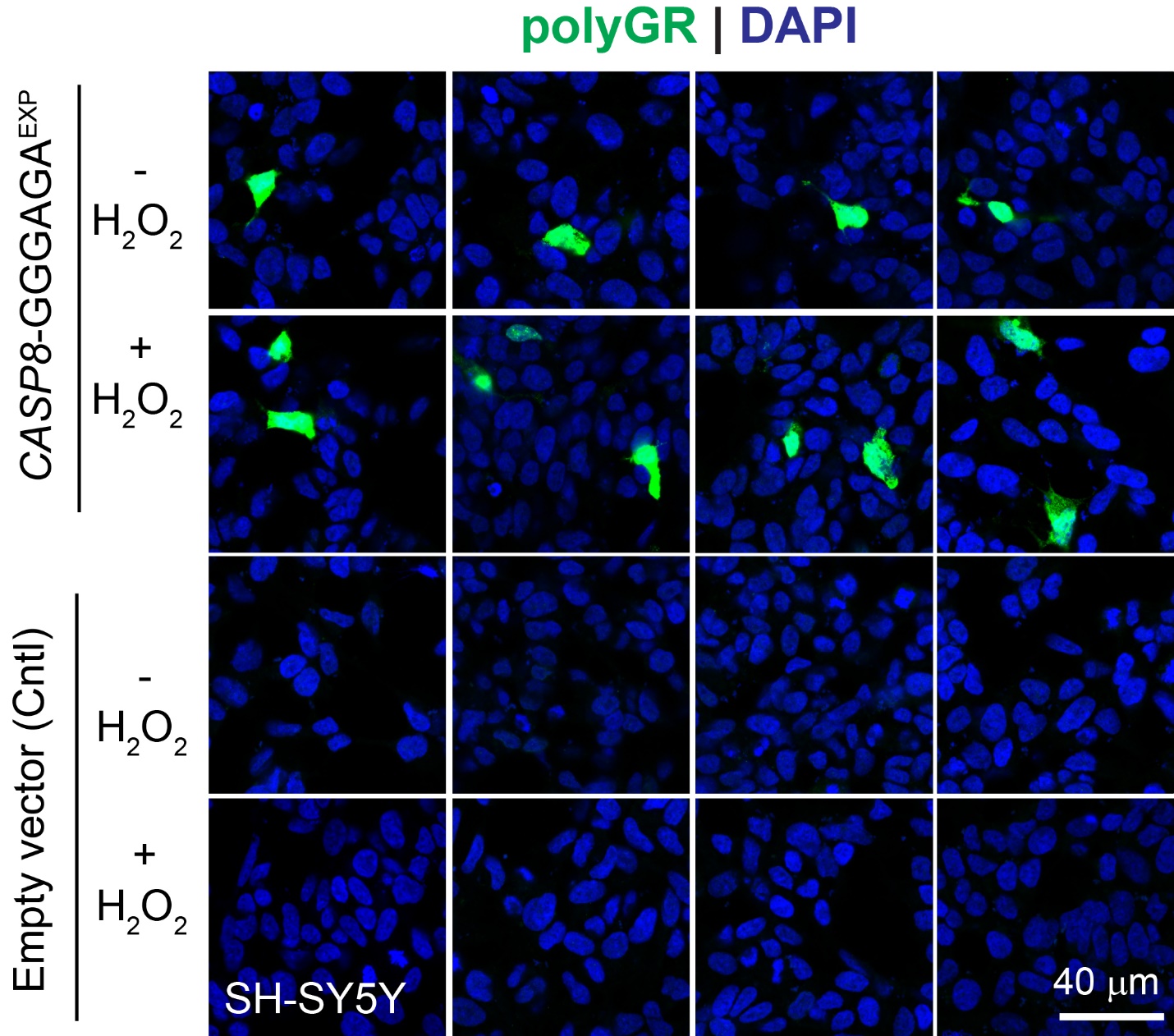
**

**Figure S28. Widefield images of polyGR+ staining in SH-SY5Y cells transfected with *CASP8*-GGGAGA^EXP^ or empty vector control plasmids, with or without hydrogen peroxide (H_2_O_2_, 50 μM) treatment**. *CASP8*-GGGAGA^EXP^ plasmids are p-AD-R1 or p-C-Var plasmids shown in Fig. 6A in Nguyen et al. [58].


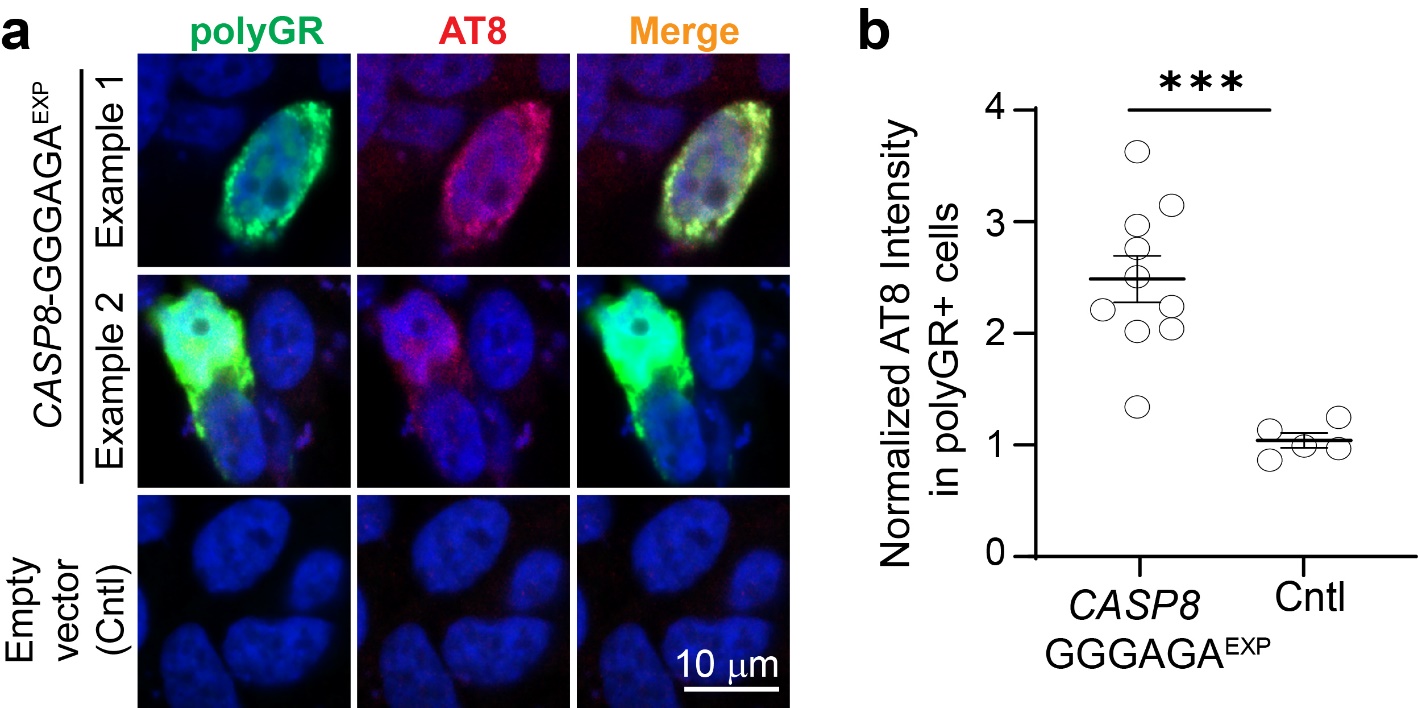


**Figure S29. Levels of** **pTau are increased in SH-SH5Y cells transfected with *CASP8*-GGGAGA^EXP^ plasmids.** (a) Representative images of polyGR+ protein and pTau (AT8, S202/T205) staining in SH-SY5Y cells transfected with *CASP8*-GGGAGA^EXP^ plasmids (p-AD-R1 or p-C-Var plasmids shown in Fig. 6A in Nguyen et al. [58]). (b) Quantification of pTau signal in SH-SY5Y cell transfected with *CASP8*-GGGAGA^EXP^ (n = 10) and empty vector control (Cntl) plasmids (n = 5), pTau signal was normalized to the number of polyGR-positive cells. Data represents mean ± SEM. Statistical analysis was performed using unpaired two-tailed Welch’s t-test. **** p<0.0001

**
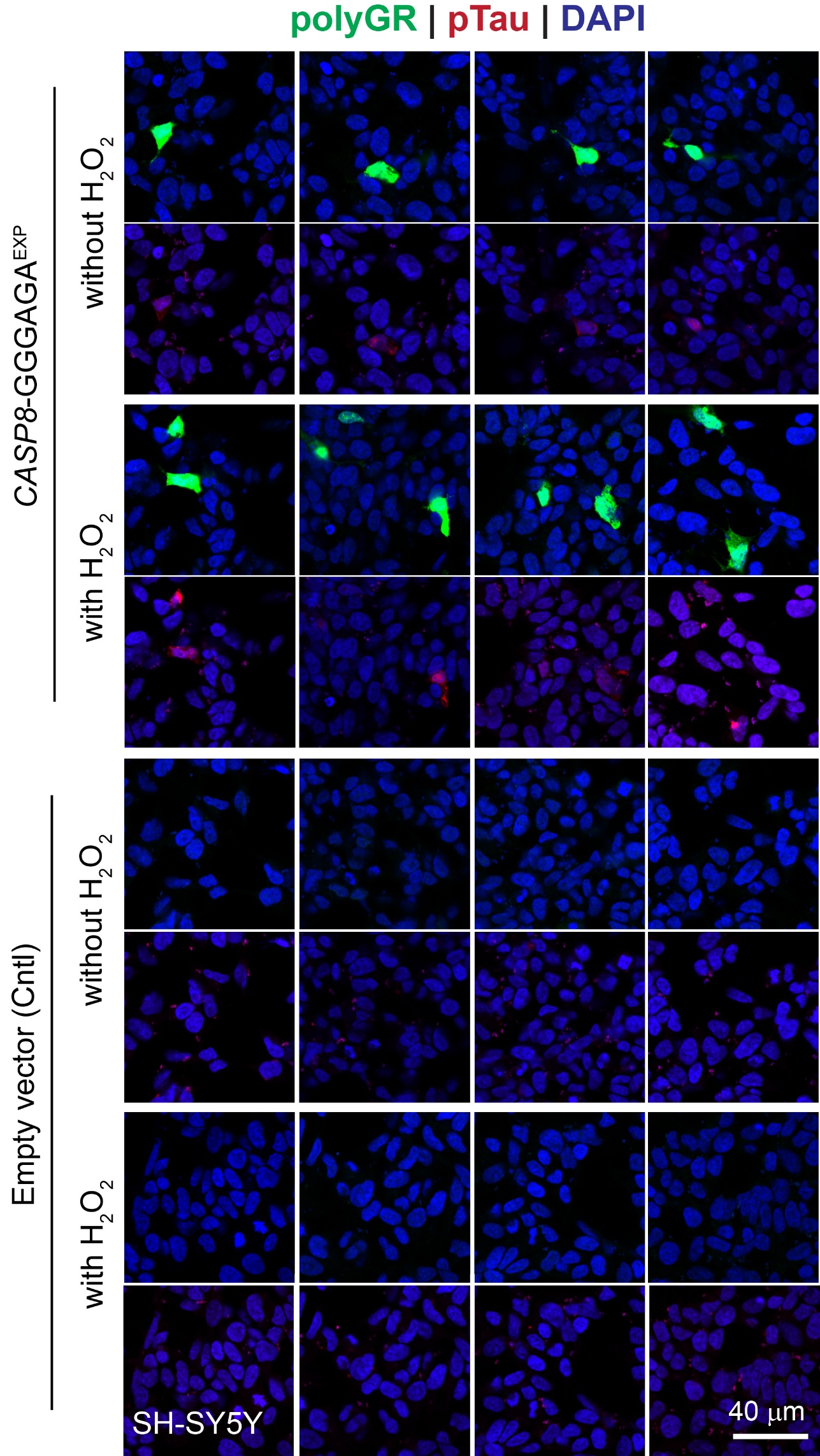
**

**Figure S30a. Widefield images of double IF staining for polyGR and pTau showing effects of hydrogen peroxide (H_2_O_2_) treatment in transfected SH-SY5Y cells.** Representative images showing staining of *CASP8* polyGR+ proteins and pTau in SH-SY5Y cells transfected with *CASP8*-GGGAGA^EXP^ plasmids (p-AD-R1 or p-C-Var plasmids shown in Fig. 6A in Nguyen et al. [58]) with or without H_2_O_2_ treatment (50 μM).


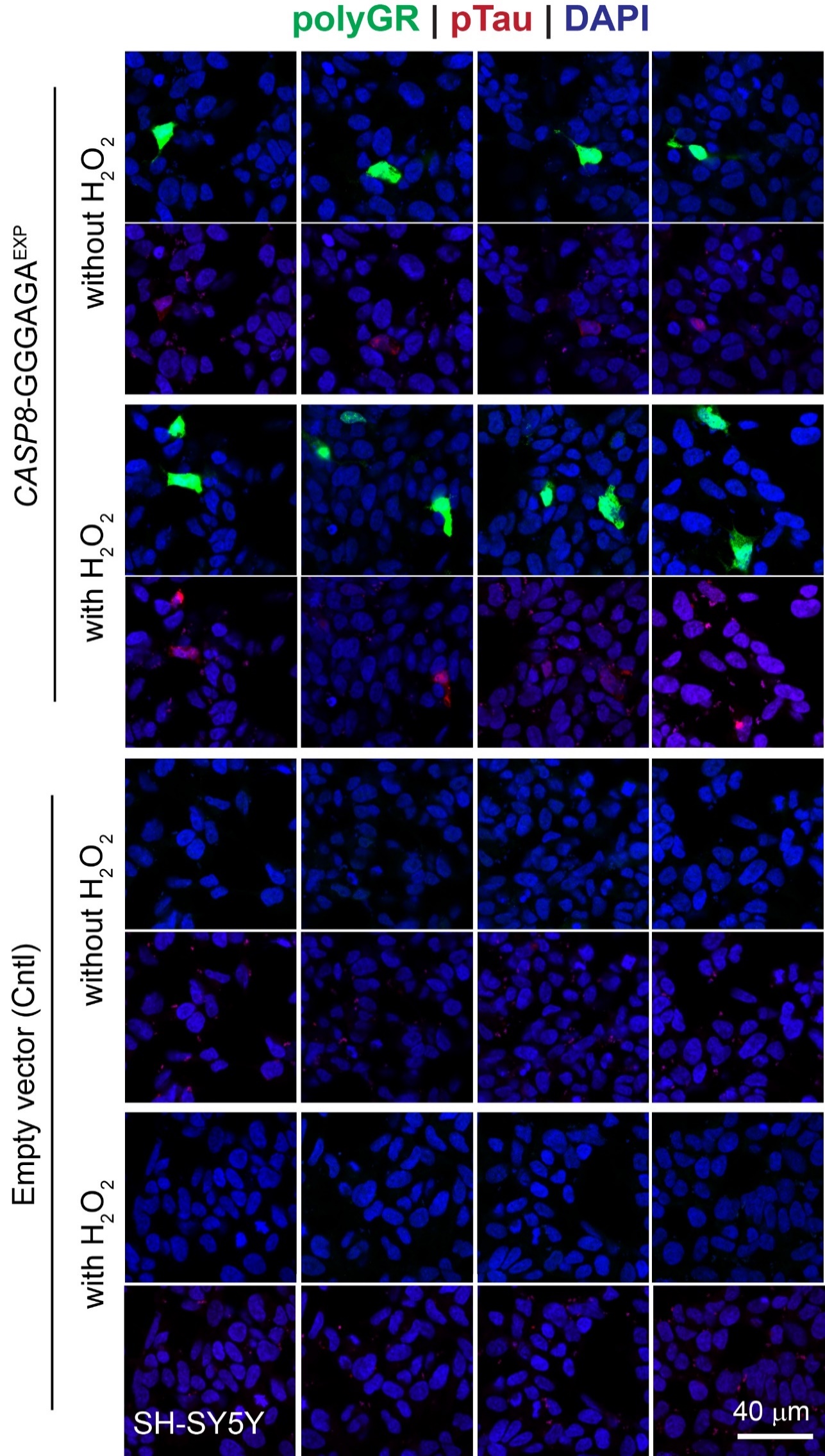


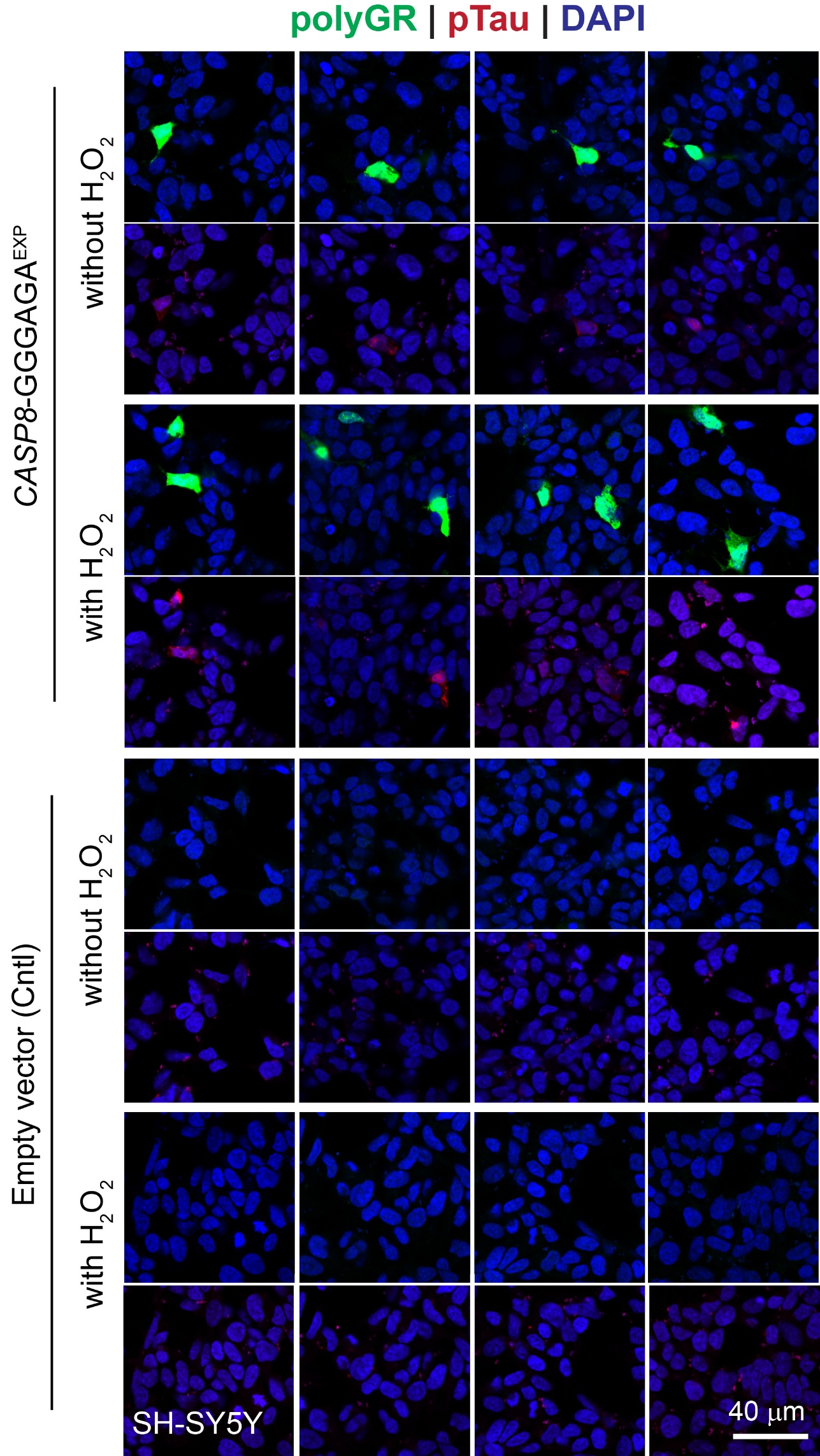


**Figure S30b. Widefield images of double IF staining for polyGR and pTau showing effects of hydrogen peroxide (H_2_O_2_) treatment in transfected SH-SY5Y cells.** Representative images showing staining of *CASP8* polyGR+ proteins and pTau in SH-SY5Y cells transfected with empty vector control (Cntl) plasmids, with or without H_2_O_2_ treatment (50 μM).


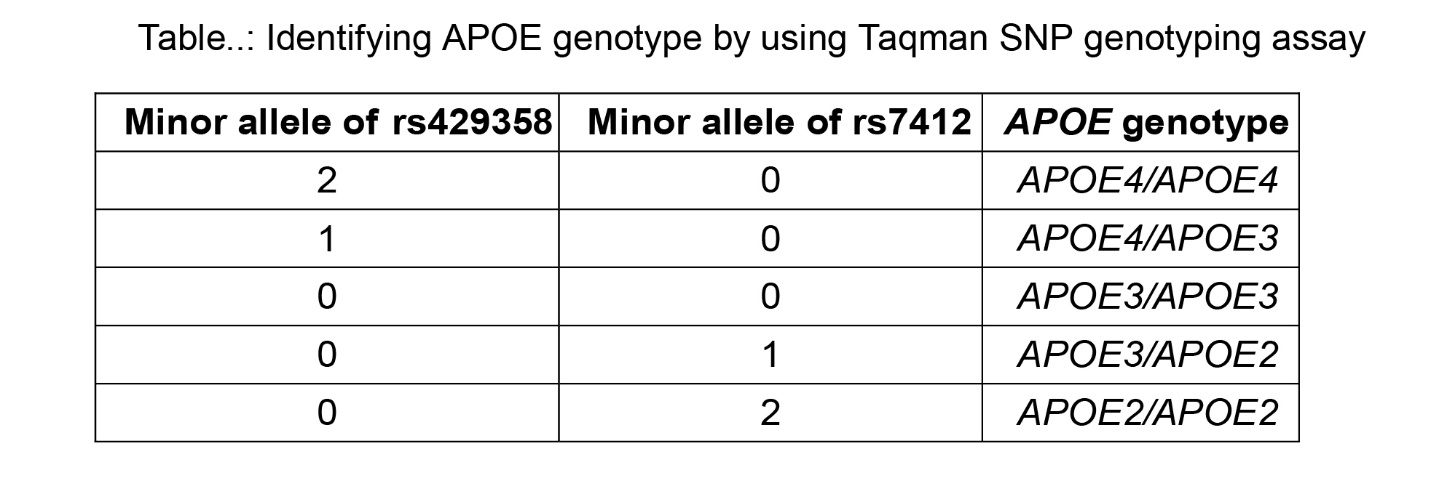


**Table S1. Calling *APOE* genotypes using TaqMan SNP assay.**

|  | **High Cholesterol** | **Heart**  **Disease** | **Heart Attack** | **Depression/**  **Anxiety** | **Cancer** | **Diabetes** | **Joint Disease** | **Loss of Consciousness** | **cerebral amyloid angiopathy** | | |
| --- | --- | --- | --- | --- | --- | --- | --- | --- | --- | --- | --- |
| **Yes** | **68** | **35** | **8** | **67** | **33** | **20** | **37** | **25** | **1** | **2** | **3** |
| **No** | **56** | **89** | **117** | **57** | **90** | **104** | **87** | **100** | **32** | **27** | **67** |

**Table S2. Additional information of comorbidities in LOAD cases.** cerebral amyloid angiopathy (CAA), 1-Focal neurological deficit, 2-Widespread mild to moderate, 3-Widespread moderate to severe. Comorbidity information was collected based on clinical assessment and medical record.

| F-3’UTR-pcDNA3.1 | CGATTACGCATAGCGAGCAG |
| --- | --- |
| R-3’UTR-pcDNA3.1 | CTAGAAGGCACAGTCGAGGC |
| GAPDH-F2 | ACCACAGTCCATGCCATCAC |
| GAPDH-R2 | TCCACCACCCTGTTGCTGTA |
| HCR-GGGAGA-Probe1 | GGGAGAGGGAGAGGGAGAGGGAGAG |
| HCR-*CASP8*-GGGAGA^EXP^-Probe2 | GTCAAATTCTTATCTATCAATGTTA |

**Table S3: List of primers and HCR-FISH probes**
