# Supplementary material for: PolyGR-containing aggregates link with pathology and clinical features of Alzheimer’s disease
Source: Acta Neuropathol. 2025 Nov 8;150(1):49. doi: 10.1007/s00401-025-02954-8 (PMC12598690; doi:10.1007/s00401-025-02954-8)
Supplement: Supplementary file 3 — Supplementary file3 (DOCX 5198 KB) [file 401_2025_2954_MOESM3_ESM.docx]

**polyGR-containing aggregates link with pathology and clinical features of Alzheimer’s disease**

Huong T. Phuong^1, 2 #^, Rodrigo F. Tomas^1, 2 #^, Cemal Akmese^1^, Ana Mijares^1, 2^, Shu Guo^1, 2^, Logan R. Bell^1, 2^, Svitlana Yegorova^1, 2^, Isabella M. Grestin^1,2^, Stefani K. Ng^1^, Grace Massey^1^ Jennifer Phillips^3, 4^, Alexandra Melloni^5^, Olga Pletnikova^6^, H. Brent Clark^7^, Juan C. Troncoso^6^, Bradley Hyman^5^, Stefan Prokop ^3, 4, 8, 9,10^, Laura P. W. Ranum^1, 2, 8, 9, 10, 11^, Lien Nguyen^1, 2, 8, 10, 11^*

^1^Center for Neuro*G*enetics, College of Medicine, University of Florida; ^2^Department of Molecular Genetics & Microbiology, College of Medicine; University of Florida;^3^Department of Pathology, Immunology and Laboratory Medicine, College of Medicine, University of Florida; ^4^Center for Translation Research in Neurodegenerative Disease, College of Medicine University of Florida; ^5^MassGeneral Institute for Neurodegenerative Disease, Massachusetts General Hospital; ^6^Department of Pathology, The Johns Hopkins University School of Medicine; ^7^Department of Laboratory Medicine and Pathology, University of Minnesota; ^8^McKnight Brain Institute, University of Florida; ^9^Department of Neurology, College of Medicine, University of Florida; ^10^Norman Fixel Institute for Neurological Disease, University of Florida; ^11^Genetics Institute, University of Florida.

^#^ Authors contribute equally to this work

* Corresponding author: Lien Nguyen (lien.nguyen@ufl.edu)

**Supplemental figures and tables**


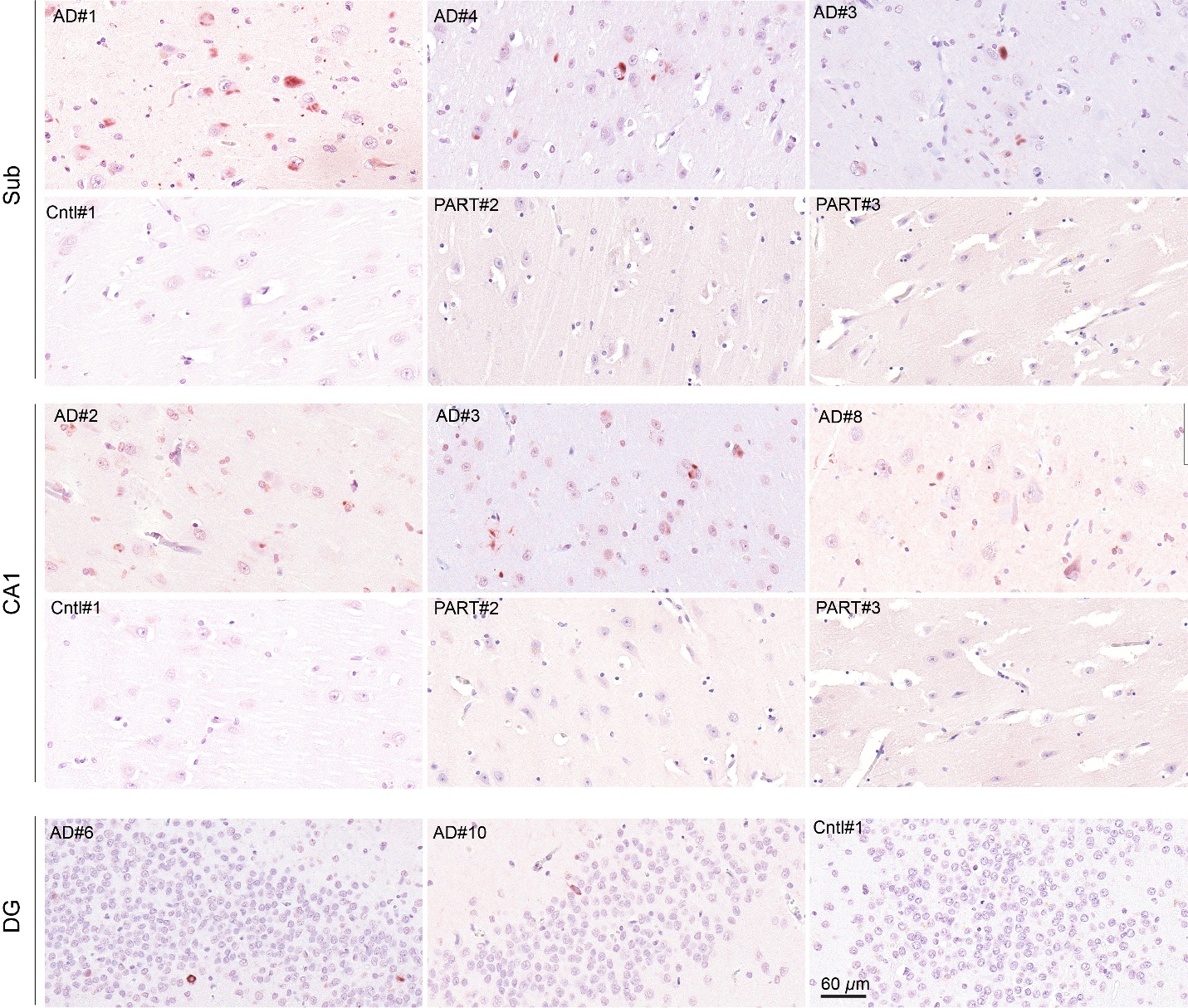


**Figure S1. Widefield images of polyGR+ staining in hippocampal regions from AD and control autopsy brains.** PART: Primary Age-Related Tauopathy, Sub: Subiculum, CA: Cornu Ammonis and DG: Dentate Gyrus

**
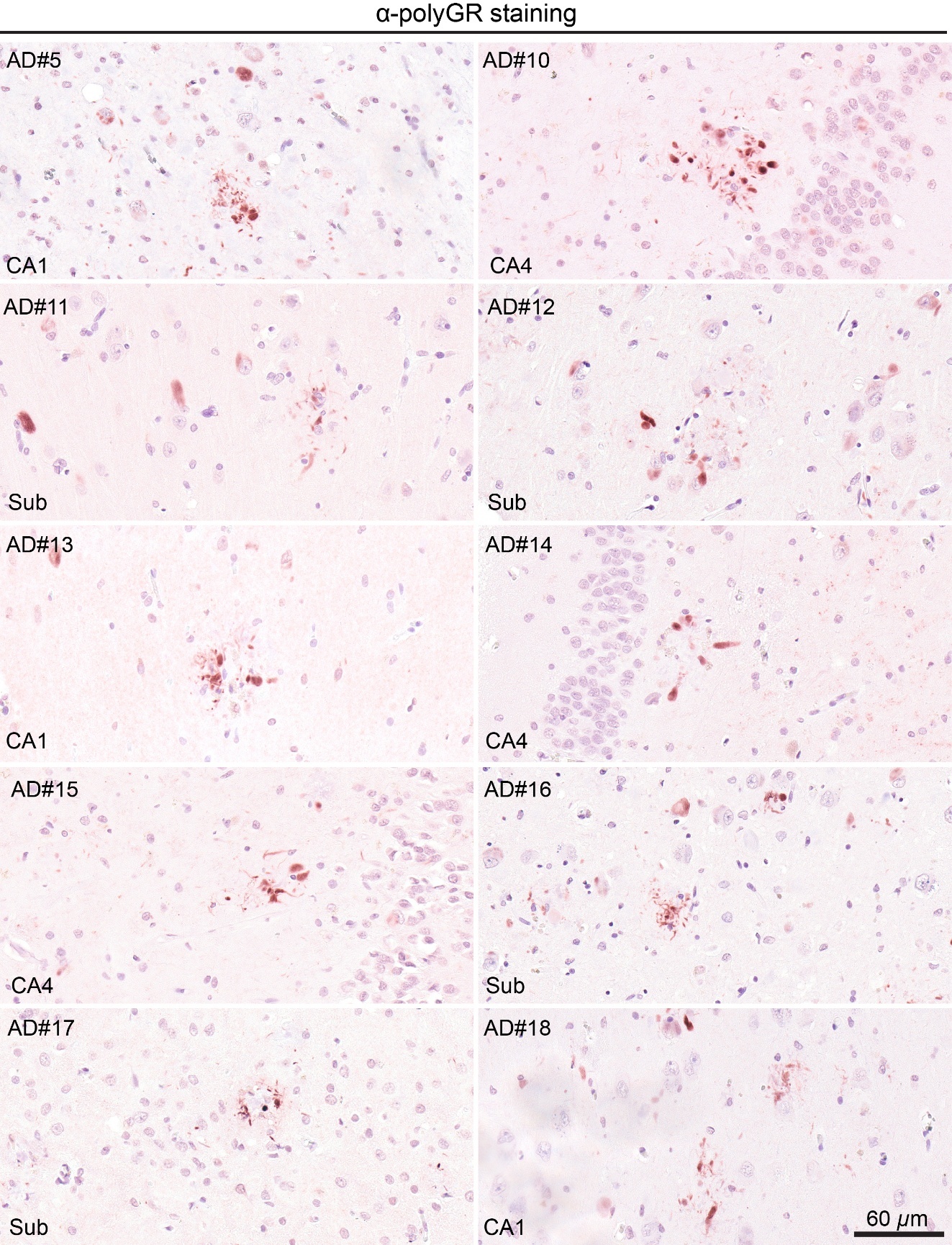
**

**Figure S2. Widefield images of immunohistochemical staining showing clustered-punctate polyGR+ staining detected in hippocampal regions from AD autopsy brains.** Sub: Subiculum, CA: Cornu Ammonis and DG: Dentate Gyrus

**
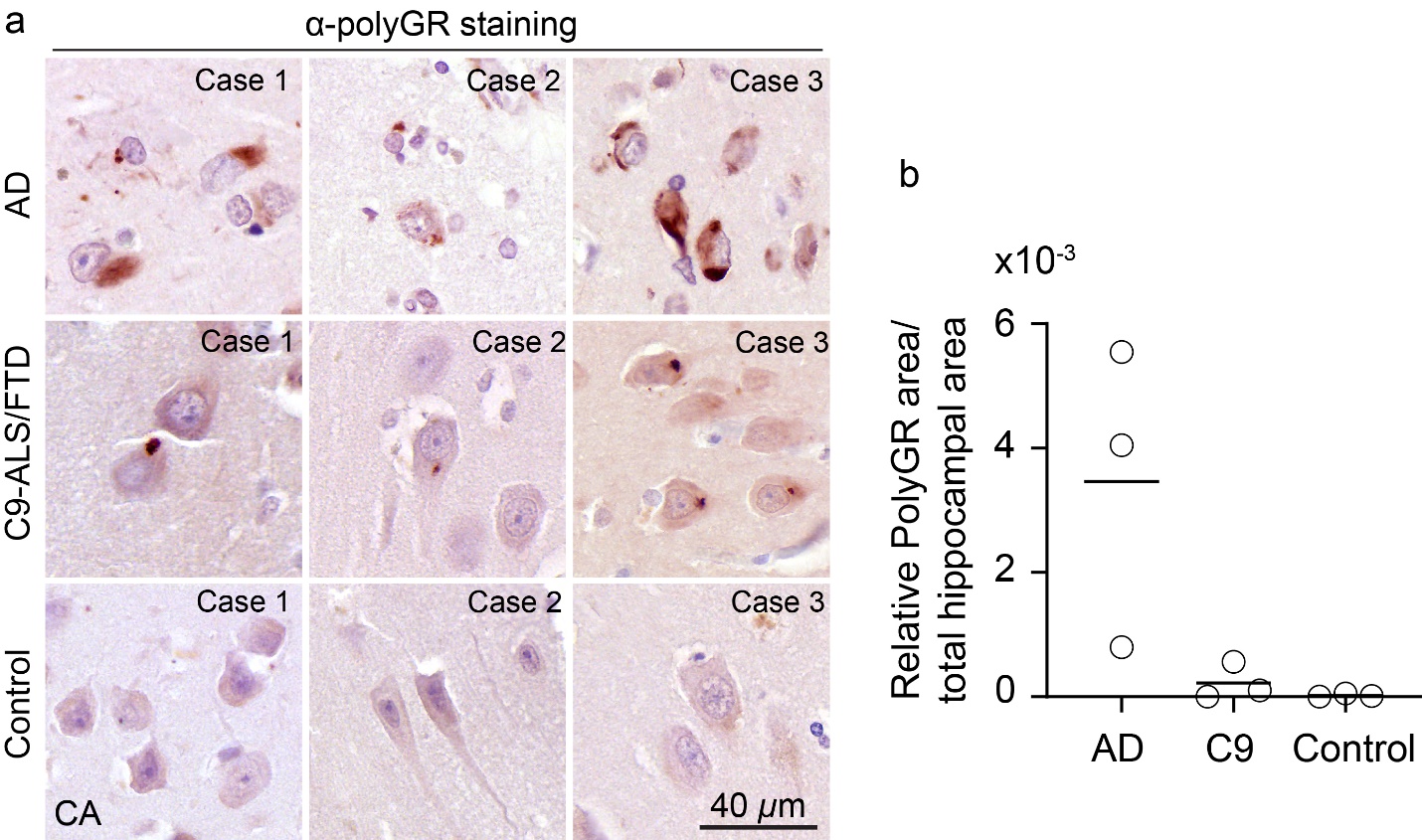
**

**Figure S3. PolyGR+ staining patterns in AD and C9-ALS/FTD autopsy brain tissue.** (a) Examples of immunohistochemical staining showing distinct accumulation patterns of polyGR+ staining in the Cornu Ammonis (CA) regions from AD and C9-ALS/FTD autopsy brains. PolyGR+ aggregates in AD autopsy brains present large puncta or fibrillary staining in cells with large or small nuclei. PolyGR aggregates in C9-ALS/FTD brains present as star-shape like puncta in hippocampal pyramidal cells, consistent to the polyGR staining described in the previously published studies. No similar polyGR+ staining was detected in the hippocampal regions from control autopsy brain tissue samples. Each image panel represents different AD, C9-ALS/FTD, or control cases. (b) Qualification of polyGR+ aggregates in hippocampus of AD, C9-ALS/FTD, and control autopsy brains. Data represents mean.


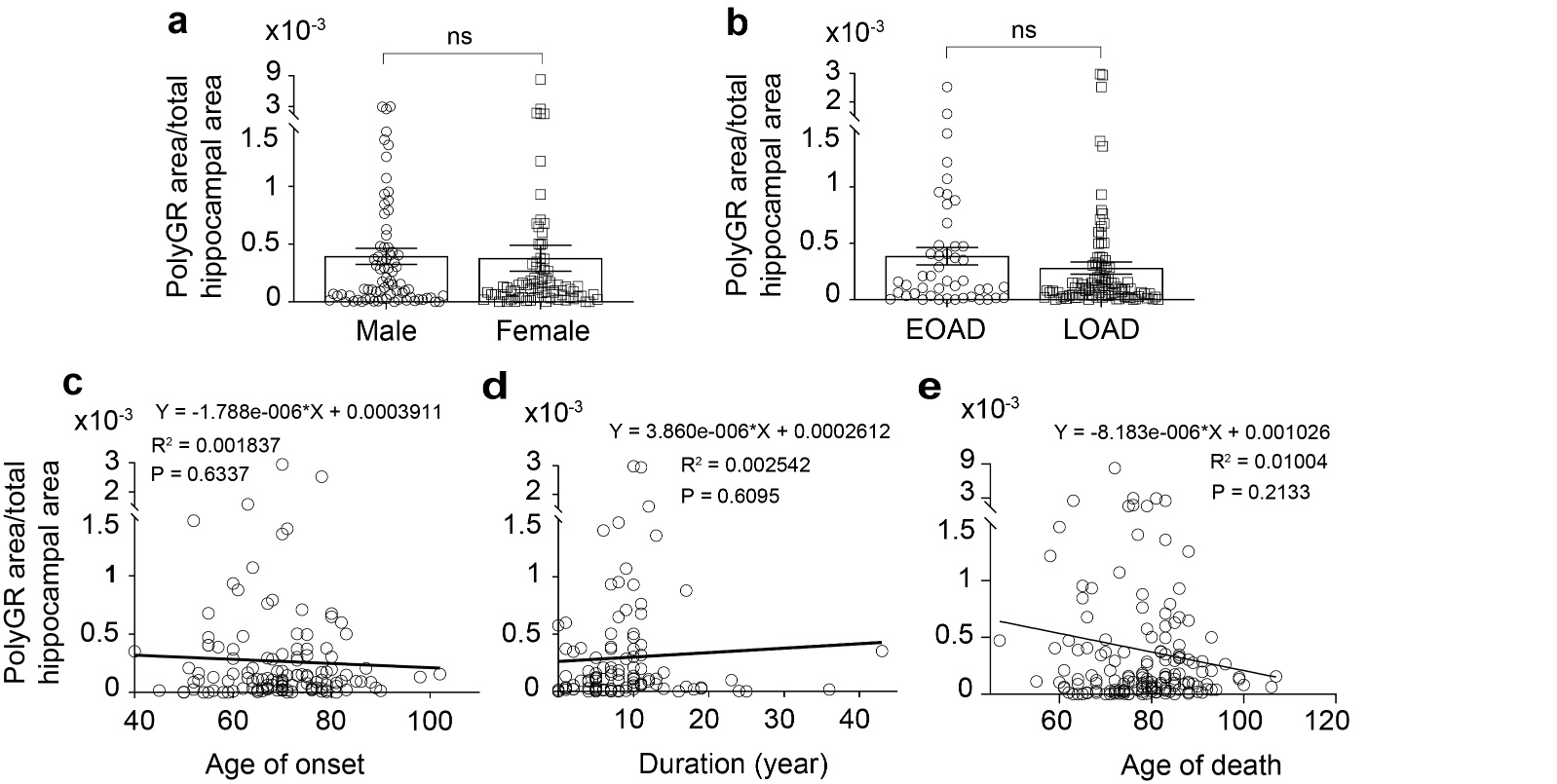


**Figure S4. Levels of polyGR+ aggregates in the hippocampal regions (HC) from AD autopsy brains are not associated with age of onset, sex, disease duration and age of death of AD patients.** (a) No difference was observed for hippocampal polyGR+ levels between male (n = 77) vs female AD cases (n = 79). (b) Graph showing no difference in polyGR+ aggregate levels in the hippocampal regions from autopsy brains of early onset AD (EOAD, n=46) and late onset AD (LOAD, n=97) AD cases. (c, d, e) Simple linear regression shows no correlation of total polyGR+ aggregate levels in the hippocampal regions from AD autopsy brains with age of onset (c) (n = 126), disease duration (d) (n = 105), and age at death (e) (n = 156). Data represents mean ± SEM (a, b) Statistical analyses were performed using unpaired two-tailed Welch’s t-test. ns p>0.05.


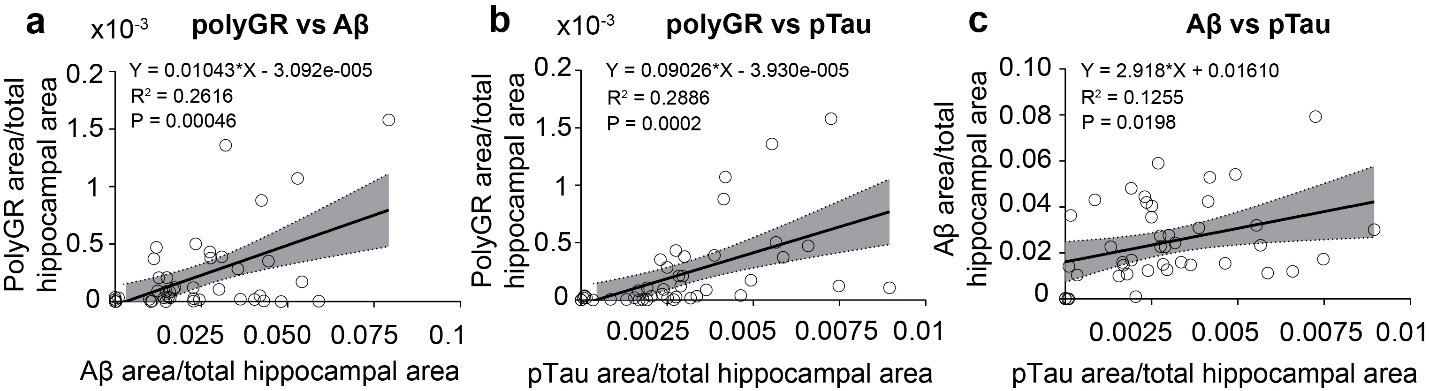


**Figure S5. Total PolyGR+ aggregate levels correlate with levels of Aβ plaques and pTau in the hippocampal regions from AD autopsy brians.** The data were plotted for a sub-cohort of 43 AD cases whose data of three pathological staining are available**.** (a, b) Simple linear regression plots showing a strong association of levels of polyGR+ aggregates with Aβ plaques (a) and pTau (AT8, S202/T205) (b). (c) Simple linear regression plot of levels of Aβ plaques and pTau (AT8, S202/T205).


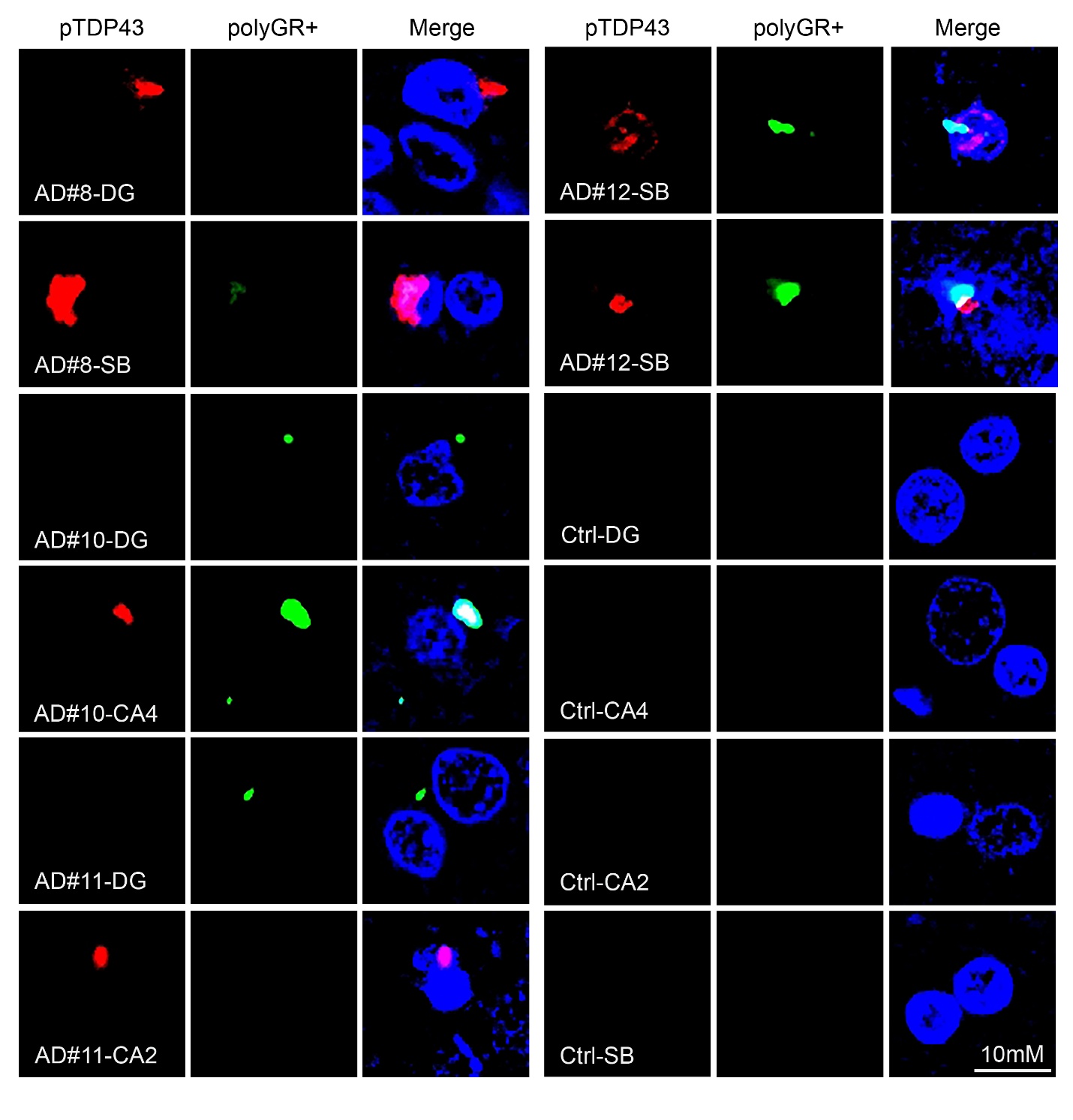


**Figure S6. Widefield double staining images of polyGR+ staining and pTDP-43 in hippocampal regions from AD and control autopsy brains.** PolyGR (green) and pTDP-43 (red) staining was co-localized or found in the same cell as well as in different cells in the hippocampal regions (Sub: Subiculum, CA: Cornu Ammonis, DG: Dentate Gyrus) from AD autopsy brains. Each image panel represents individual AD or control cases. AD cases in this staining were reported with limbic-predominant age-related TDP-43 encephalopathy (LATE) stage 2 (TDP-43 pathology is present in the hippocampus and entorhinal cortex).


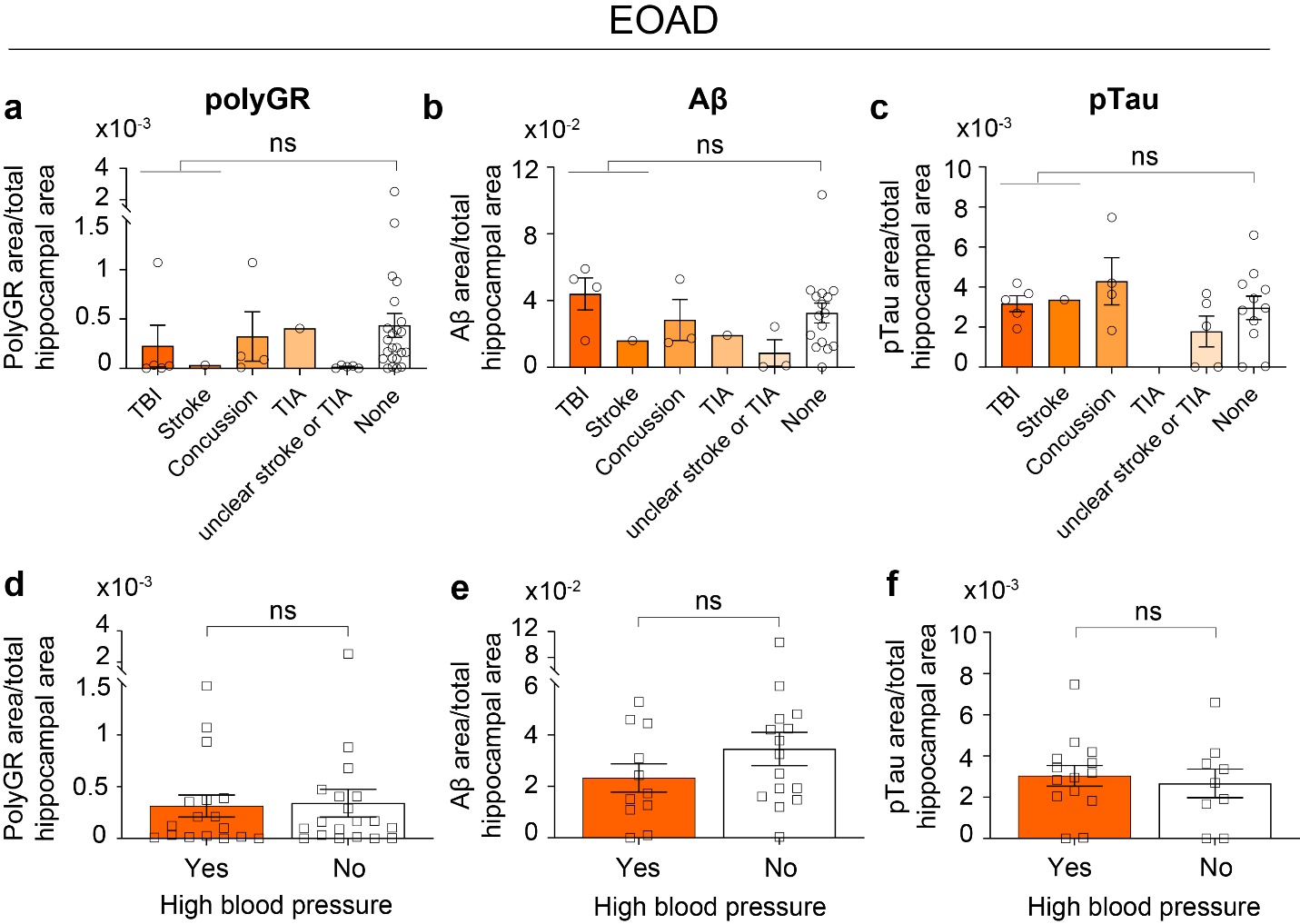


**Figure S7. PolyGR+ aggregate levels in early onset AD (EOAD) cases with or without TBI, Stroke, TIA or high blood pressure.** (a, b, c) Plots showing levels of polyGR+ aggregates (a) (n = 36), Aβ plaques (b) (n = 26), and pTau (c) (n = 23) EOAD cases with or without a history of brain injuries (TBI, concussion, stroke, and TIA). (d, e, f) Plots showing levels of polyGR+ aggregates (d) (n = 36), Aβ plaques (e) (n = 26), and pTau (f) (n = 23) in EOAD cases with or without a history of high blood pressure. Data represents mean ± SEM. Statistical analyses of TBI & Stroke with none TBI/Stroke/TIA were performed using unpaired two-tailed T-test. Statistical analyses of high blood pressure were performed using unpaired two-tailed Welch’s t-test. ns p>0.05.


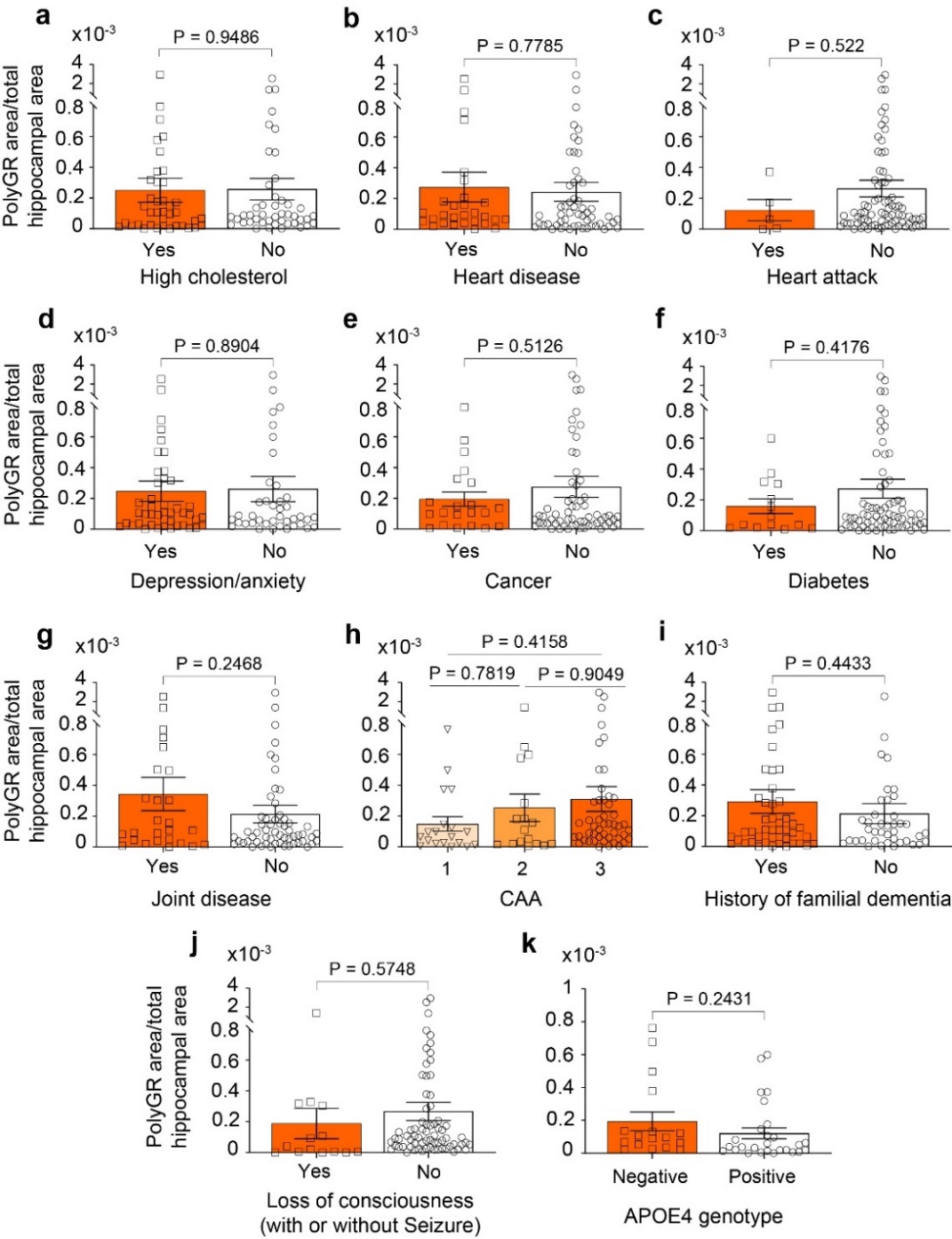


**Figure S8. Total hippocampal polyGR+ aggregate levels in LOAD cases with additional comorbidities.** Levels of polyGR+ aggregates in the hippocampal regions from LOAD autopsy brains were compared between AD cases with and without (a) high cholesterol (n=84), (b) heart disease (n=84), (c) heart attacks (n=85), (d) depression/anxiety (n=84), (e) cancer (n=83), (f) diabetes (n=84), (g) joint disease (n=84), (h) cerebral amyloid angiopathy (CAA) (n=81 with three categories: 1-Focal, 2-Widespread mild to moderate, 3-Widespread moderate to severe, (i) Family history with dementia (n=85), (j) loss of consciousness with or without seizure (n=84), and (k) carrying an APOE4 allele (n=44). Data represent mean ± SEM. Statistical analyses were performed using unpaired two-tailed t-test (a, b, c, d, e, f, g, i, j, k) or One-way ANOVA with Brown-Forsythe test (h). ns: p>0.05.

**
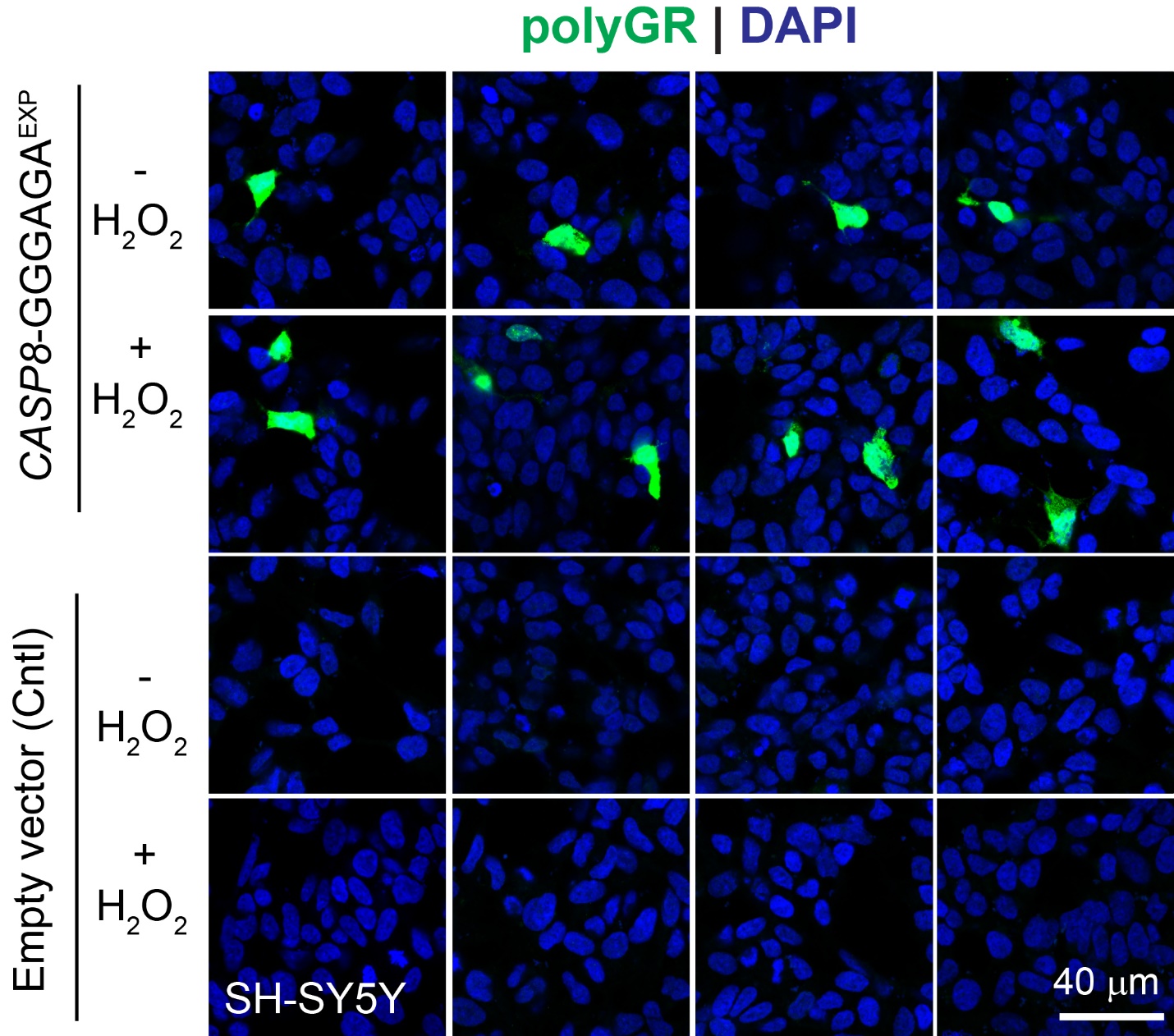
**

**Figure S9. Widefield images of polyGR+ staining in SH-SY5Y cells transfected with *CASP8*-GGGAGA^EXP^ or empty vector control plasmids, with or without hydrogen perioxide (H_2_O_2_, 50 μM) treatment**. *CASP8*-GGGAGA^EXP^ plasmids are p-AD-R1 or p-C-Var plasmids shown in Fig. 6A in Nguyen et al. [58].


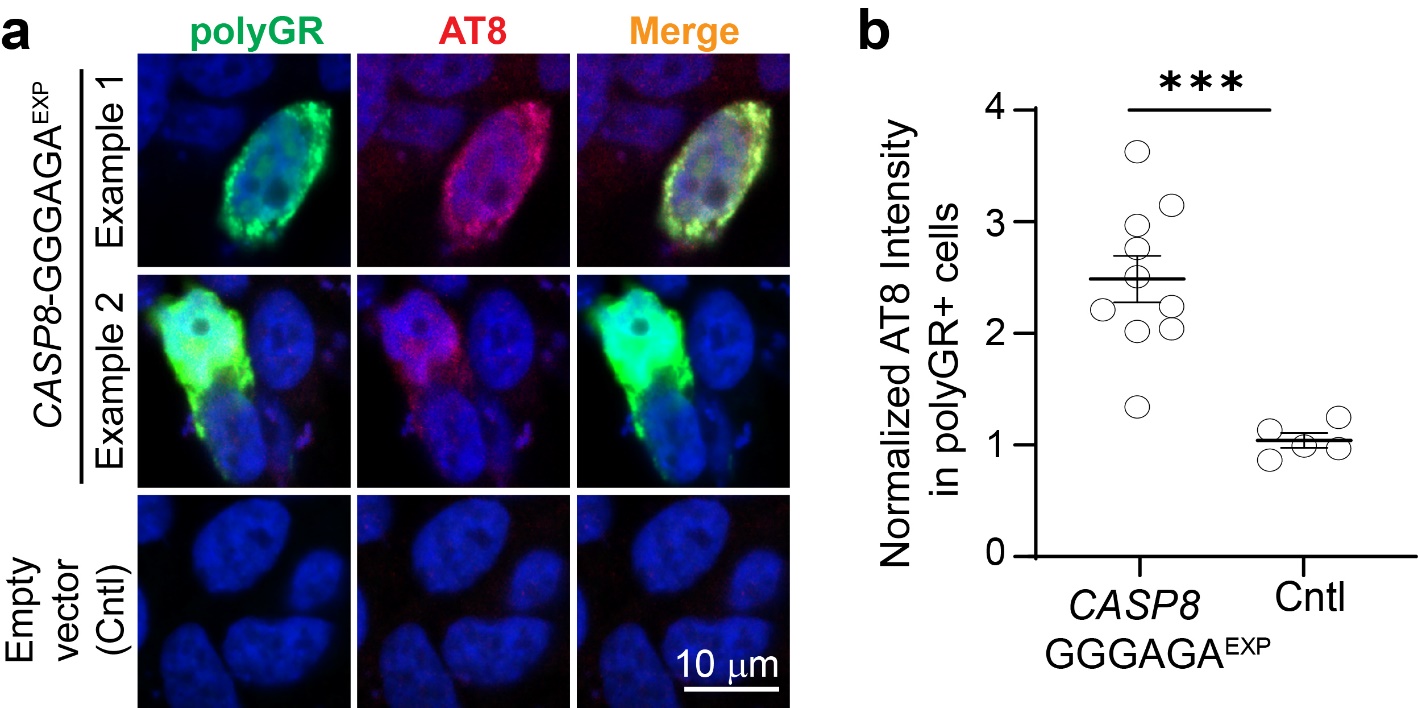


**Figure S10. Levels of** **pTau are increased in SH-SH5Y cells transfected with *CASP8*-GGGAGA^EXP^ plasmids.** (a) Representative images of polyGR+ protein and pTau (AT8, S202/T205) staining in SH-SY5Y cells transfected with *CASP8*-GGGAGA^EXP^ plasmids (p-AD-R1 or p-C-Var plasmids shown in Fig. 6A in Nguyen et al. [58]). (b) Quantification of pTau signal in SH-SY5Y cell transfected with *CASP8*-GGGAGA^EXP^ (n = 10) and empty vector control (Cntl) plasmids (n = 5), pTau signal was normalized to the number of polyGR-positive cells. Data represents mean ± SEM. Statistical analysis was performed using unpaired two-tailed Welch’s t-test.

**
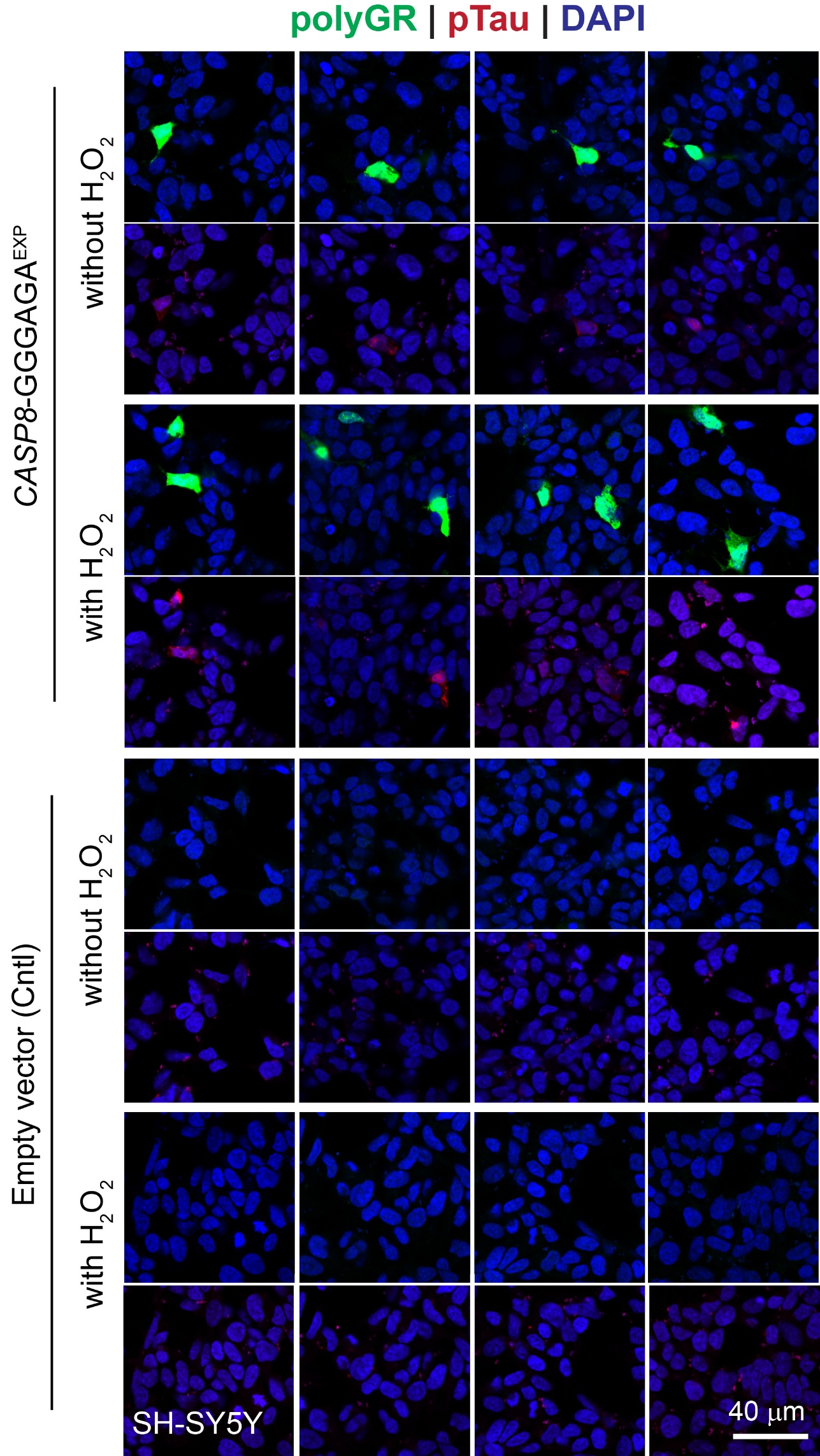
**

**Figure S11a. Widefield images of double IF staining for polyGR and pTau showing effects of hydrogen peroxide (H_2_O_2_) treatment in transfected SH-SY5Y cells.** Representative images showing staining of *CASP8* polyGR+ proteins and pTau in SH-SY5Y cells transfected with *CASP8*-GGGAGA^EXP^ plasmids (p-AD-R1 or p-C-Var plasmids shown in Fig. 6A in Nguyen et al. [58]) with or without H_2_O_2_ treatment (50 μM).


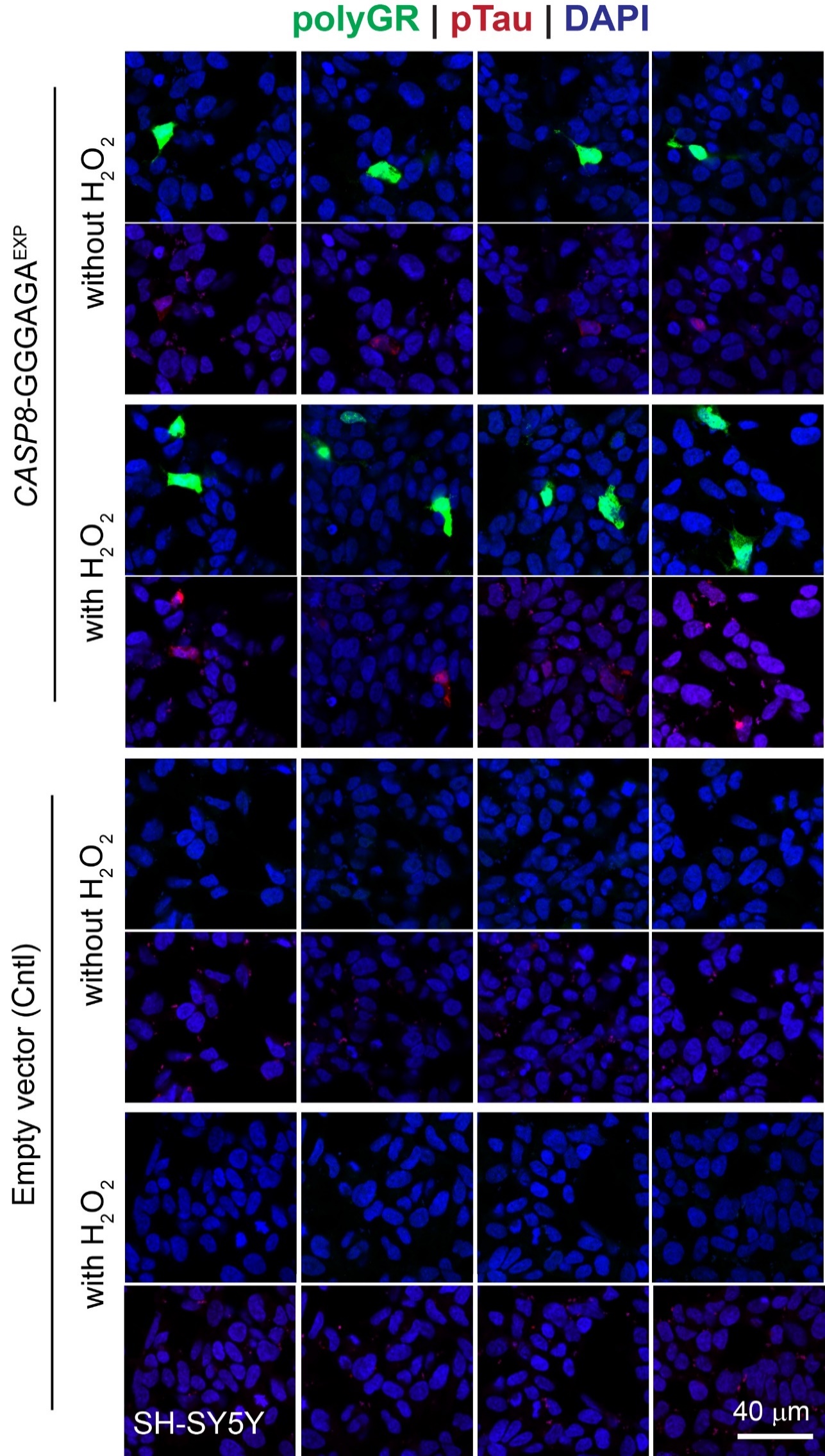


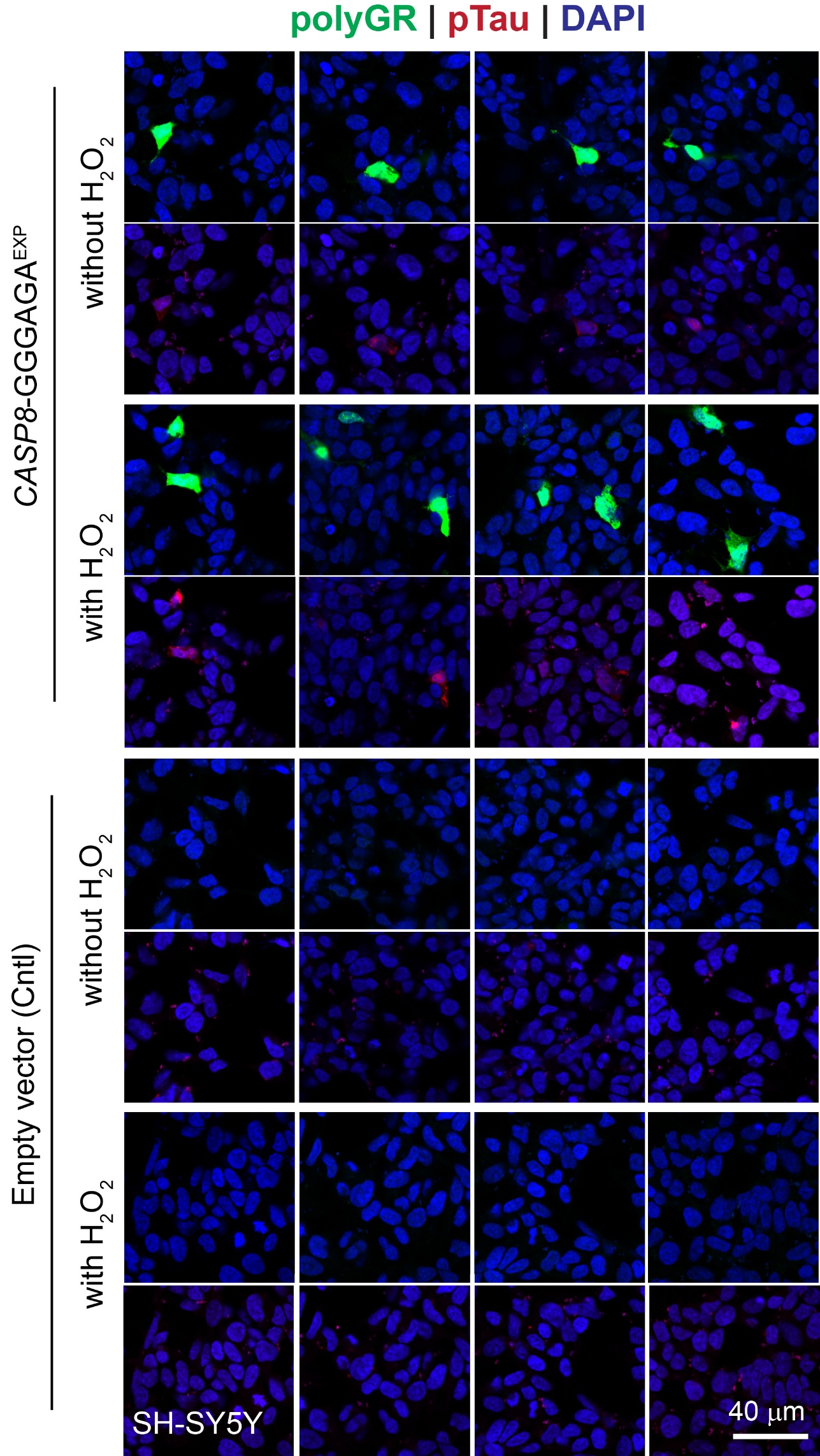


**Figure S11b. Widefield images of double IF staining for polyGR and pTau showing effects of hydrogen peroxide (H_2_O_2_) treatment in transfected SH-SY5Y cells.** Representative images showing staining of *CASP8* polyGR+ proteins and pTau in SH-SY5Y cells transfected with empty vector control (Cntl) plasmids, with or without H_2_O_2_ treatment (50 μM).

|  | **High Cholesterol** | **Heart**  **Disease** | **Heart Attack** | **Depression/**  **Anxiety** | **Cancer** | **Diabetes** | **Joint Disease** | **Loss of Consciousness** | **cerebral amyloid angiopathy** | | |
| --- | --- | --- | --- | --- | --- | --- | --- | --- | --- | --- | --- |
| **Yes** | **68** | **35** | **8** | **67** | **33** | **20** | **37** | **25** | **1** | **2** | **3** |
| **No** | **56** | **89** | **117** | **57** | **90** | **104** | **87** | **100** | **32** | **27** | **67** |

**Table S1. Additional information of comorbidities in LOAD cases.** cerebral amyloid angiopathy (CAA), 1-Focal neurological deficit, 2-Widespread mild to moderate, 3-Widespread moderate to severe. Comorbidity information was collected based on clinical assessment and medical record.

| F-3’UTR-pcDNA3.1 | CGATTACGCATAGCGAGCAG |
| --- | --- |
| R-3’UTR-pcDNA3.1 | CTAGAAGGCACAGTCGAGGC |
| GAPDH-F2 | ACCACAGTCCATGCCATCAC |
| GAPDH-R2 | TCCACCACCCTGTTGCTGTA |

**Table S2: List of primers**
